# Independent Origins of Cultivated Coconut (*Cocos nucifera* L.) in the Old World Tropics

**DOI:** 10.1371/journal.pone.0021143

**Published:** 2011-06-22

**Authors:** Bee F. Gunn, Luc Baudouin, Kenneth M. Olsen

**Affiliations:** 1 Division of Evolution, Ecology and Genetics, Research School of Biology, The Australian National University, Canberra, Australia; 2 Centre International de Recherches en Agronomie pour le Développement (CIRAD), Montpellier, France; 3 Biology Department, Washington University, St. Louis, Missouri, United States of America; University of Umeå, Sweden

## Abstract

As a portable source of food, water, fuel, and construction materials, the coconut (*Cocos nucifera* L.) played a fundamental role in human migrations and the development of civilization across the humid tropics. Here we investigated the coconut's domestication history and its population genetic structure as it relates to human dispersal patterns. A sample of 1,322 coconut accessions, representing the geographical and phenotypic diversity of the species, was examined using ten microsatellite loci. Bayesian analyses reveal two highly genetically differentiated subpopulations that correspond to the Pacific and Indo-Atlantic oceanic basins. This pattern suggests independent origins of coconut cultivation in these two world regions, with persistent population structure on a global scale despite long-term human cultivation and dispersal. Pacific coconuts show additional genetic substructure corresponding to phenotypic and geographical subgroups; moreover, the traits that are most clearly associated with selection under human cultivation (dwarf habit, self-pollination, and “*niu vai*” fruit morphology) arose only in the Pacific. Coconuts that show evidence of genetic admixture between the Pacific and Indo-Atlantic groups occur primarily in the southwestern Indian Ocean. This pattern is consistent with human introductions of Pacific coconuts along the ancient Austronesian trade route connecting Madagascar to Southeast Asia. Admixture in coastal east Africa may also reflect later historic Arab trading along the Indian Ocean coastline. We propose two geographical origins of coconut cultivation: island Southeast Asia and southern margins of the Indian subcontinent.

## Introduction

The impact of the coconut palm (*Cocos nucifera* L.) on the history of human dispersal in the humid tropics is unparalleled in the plant kingdom. As a portable source of both food and water, the coconut played a critical role in the ability of humans to voyage, establish trade routes, and colonize lands in the Pacific Rim and regions throughout the Old World tropics [1], [2]. This species continues to have hundreds of uses as a source of food, drink, fiber, construction material, charcoal, and oil (used in cooking, pharmaceuticals, industrial applications, and biofuels); over 12 million hectares of coconut are currently planted across 89 tropical countries [3]. The history of dispersal and cultivation of this species is thus fundamentally intertwined with human history in the tropics.

The long-term interaction between humans and coconuts has shaped both the geographical distribution of *C. nucifera* and its phenotypic diversity. While the coconut fruit is naturally adapted for dispersal by sea currents [4], its pantropical dissemination was achieved with the help of humans [5], [6]. A native of the Old World tropics, the species was spread to eastern Polynesia and subsequently introduced to the Pacific coasts of Latin America, most likely by pre-Columbian Austronesian seafarers from the Philippines [7]. In the Indian Ocean, the composition of coconut populations was likely influenced by Austronesian expansions westward to Madagascar. Later, coconuts were introduced by Europeans from India to the Atlantic coasts of Africa and South America and to the Caribbean [8]. The species is typically found in areas of present or past human activity, and all or nearly all coconut populations worldwide have likely been influenced by human cultivation and dispersal.

Phenotypically, coconuts vary widely in the degree to which they show evidence of selection under human cultivation. Classic analyses of coconut fruit morphology revealed two predominant fruit types, named after traditional Polynesian varieties: the ‘*niu kafa*’ form, characterized by oblong, triangular fruits with a large proportion of fibrous husk; and the ‘*niu vai*’ form, whose fruits are rounded and often brightly colored, with a large proportion of liquid endosperm [9], [10]. The ‘*niu kafa*’ form has been interpreted as the more ancestral morphology, reflecting natural selection for ocean dispersal, and the ‘*niu vai*’ form as reflecting selection under human cultivation [1]. Coconuts have also been traditionally classified into ‘Dwarf’ and ‘Tall’ varieties based on tree habit. ‘Dwarfs’ represent about 5% of coconut palms and are cultivated worldwide; they are typically found near human habitation and show traits closely associated with human selection: slow trunk growth, self-pollination, and the production of *niu vai* fruits [11]. The more common ‘Tall’ coconuts are outcrossing and grow faster than ‘Dwarfs,’ resulting in greater height at reproductive maturity. Many ‘Talls’ are grown for the production of copra for oil extraction and coir for fiber; while actively cultivated, these varieties lack the obvious domestication traits of the self-pollinating Dwarfs.

The lack of universal domestication traits among coconut varieties, combined with the long history of human interaction with this species, have made it difficult to trace the coconut's cultivation origins. However, applications of molecular markers for purposes of crop germplasm characterization have provided some insights into the coconut's evolutionary history, genetic diversity and population structure (e.g., [12], [13]). Analyses using RFLPs (e.g., [14]), microsatellites [15], [16] and AFLP markers [17] have suggested the presence of two genetically distinct groups, corresponding broadly to the Pacific Ocean basin on one side and the Indian and Atlantic Oceans on the other (see also [18], [19]).

In the last decade, a worldwide coconut germplasm collection, coordinated through the International Coconut Genetic Resources Network (COGENT) and the French Agricultural Research Centre for International Development (CIRAD), with further support through the Generation Challenge Programme (GCP: http://gcpcr.grinfo.net/index.php), has served as the primary source of materials for genetic characterizations. Together with a polymorphic microsatellite marker kit [20], the GCP/CIRAD coconut collection has been used to characterize genetic diversity in regional coconut collections (e.g., [21], [22]), infer origins of specific cultivars [7], and assess planting material for trueness to type [23]. Importantly, this worldwide collection has not been used previously to examine the coconut's cultivation history. Moreover, while global in scope, the GCP/CIRAD collection has left some geographical regions under-represented. Most notably, it contains few coconuts from the western Indian Ocean, which would be key to elucidating any influence of ancient Austronesian expansions in this region.

In the present study, we have employed ten polymorphic loci from the GCP/CIRAD microsatellite kit to examine genetic variation in a worldwide collection of >1300 coconuts, representing GCP/CIRAD germplasm plus collections from key under-sampled regions of the western Indian Ocean: Madagascar, Comoros, and Seychelles islands. We use population structure analyses, together with ethnographic and archaeobotanical evidence, to examine the impacts of human-mediated dispersal and domestication on this important tree crop. Our analyses suggest the following: 1) Despite the widespread movement of coconuts by humans, both historically and today, the species has retained clear population structure on a global scale; 2) Present-day cultivated coconuts arose through independent domestications in the Indian and Pacific Ocean basins; however, the definitive domestication traits — dwarf habit, self-pollination, and *niu vai* fruits — arose only with the Pacific domestication event; and 3) Geographical locations of genetically admixed populations are consistent with human introductions of Pacific germplasm along the ancient trading routes connecting Asia to Africa.

## Results

With new sample collections that fill an important gap in an already extensive worldwide data set, we have examined variation at ten microsatellite loci in a global collection of coconut germplasm. Genotypes were successfully obtained for 1322 samples, representing 1210 individuals from the GCP/CIRAD collection and 112 samples from the western Indian Ocean (Table S1). For germplasm characterization purposes, the GCP/CIRAD collection has previously been categorized into a hierarchical classification scheme based on a combination of criteria, including phenotypes, molecular markers, geographic distribution, and known introduction history [7]. Compositions of the 16 GCP/CIRAD groups and three additionally sampled Indian Ocean regions are shown in Table 1. The highest level in the GCP/CIRAD classification divides coconuts into two groups, A and B. Group A coconuts occur primarily in the region spanning Southeast Asia to the Pacific coast of America. Group B coconuts occur across coastal S. Asia, W. Africa, the New World Atlantic, and the Caribbean [2], [14]. Subgroups correspond to geographical and/or phenotypic subsets within each group (Table 1); the greater number of subgroups for Group A coconuts reflects this group's higher phenotypic diversity.

**Table 1:** Genetic diversity and population structure in a worldwide sample of coconuts^a^.

Population (Group)	N (cvs)	Growth Form	Primary Region	H_e_	H_o_	Q_1_	Q_2_
**1** (A1a)	16 (9)	Dwarf	worldwide	0.270	0.081	**0.966**	0.034
**2** (A1b)	32 (7)	Dwarf	SE Asia	0.239	0.099	**0.994**	0.006
**3** (A2)	6 (4)	Dwarf	worldwide	0.303	0.000	**0.985**	0.015
**4** (A3a)	66 (9)	Tall	SE Asia	0.612	0.532	**0.927**	0.073
**5** (A3b)	25 (5)	Tall	SE Asia	0.556	0.428	**0.976**	0.024
**6** (A3c)	89 (10)	Tall	SE Asia	0.583	0.447	**0.988**	0.012
**7** (A4a)	38 (8)	Tall	PNG^c^	0.607	0.499	**0.990**	0.010
**8** (A4b)	34 (8)	Tall	PNG	0.596	0.522	**0.990**	0.010
**9** (A4c)	48 (10)	Tall	PNG	0.564	0.484	**0.986**	0.014
**10** (A4d)	21 (3)	Tall	PNG	0.610	0.586	**0.991**	0.009
**11** (A4e)	360 (10)	Tall	Melanesia	0.624	0.547	**0.980**	0.020
**12** (A5)	43 (11)	Tall	Micronesia	0.644	0.508	**0.881**	0.119
**13** (A6)	30 (6)	Tall^b^	Polynesia	0.644	0.529	**0.944**	0.056
**14** (A7)	105 (5)	Tall	Panama	0.324	0.230	**0.950**	0.050
**15** (B1)	150 (18)	Tall	S. Asia+Atlantic	0.483	0.364	0.030	**0.970**
**16** (B2)	147 (14)	Tall	E. Africa	0.640	0.570	0.150	**0.850**
**17 —**	13 (—)	Tall	Comoros	0.672	0.544	0.426	0.574
**18 —**	44 (—)	Tall	Madagascar	0.691	0.546	0.333	0.667
**19 —**	55 (—)	Tall	Seychelles	0.413	0.351	0.018	**0.982**

a Group labels correspond to GCP/CIRAD designations. N = sample sizes, cvs = number of named cultivars. H_e_ = mean unbiased gene diversity, H_o_ = mean observed heterozygosity, and Q_1_ and Q_2_ indicate subpopulation membership coefficients in *Structure* analyses at K = 2 subpopulations. Bold font indicates membership coefficients of Q≥80%.

b includes ‘*Niu Leka*,’ an outcrossing compact-growth variety that is phenotypically distinct from other ‘Dwarfs.’

c Papua New Guinea.

### Within-group genetic diversity

Genetic diversity for Dwarf coconut varieties (populations 1–3; Table 1) is on average less than half that of Talls, with mean unbiased gene diversity values of H_e_ = 0.271 and 0.579 for the two growth forms, respectively. Dwarfs also show greater evidence of inbreeding (mean observed heterozygosity, H_o_ = 0.060 and 0.480 for Dwarfs and Talls, respectively), consistent with the low within-cultivar genetic heterogeneity characterizing these self-pollinating varieties, most of which are pure-breeding lines. This overall pattern of reduced genetic variability in Dwarfs has been reported previously (e.g., [18]) and is consistent with domestication bottlenecks during the evolution of these highly selected cultivars. Among Talls, genetic diversity is lowest for the Pacific coast Latin American collections (‘Panama Talls’) (population 14; He = 0.324; Table 1), concordant with a founder event in their prehistoric introduction from Southeast Asia [7].

### Global genetic differentiation and independent origins of domestication

**Figure 1:**
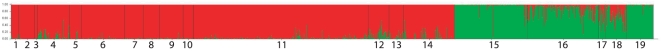
Results of *Structure* analysis for a worldwide sample of 1322 coconuts. Population assignments for each accession are shown at K = 2 subpopulations. Numbers along the x-axis correspond to group designations in Table 1. Vertical black lines distinguish the population groups.

Consistent with earlier molecular marker studies (e.g., [14]–[18]), our population structure analysis using a worldwide sample set indicates that coconuts are differentiated into two major subpopulations. We performed Bayesian analyses using *Structure* 2.3 [24], with K (the number of putative genetic subpopulations) ranging from 1 to 10, and assessed rates of change in log likelihood values. The optimal value, as determined by the *ad hoc* criterion ΔK [25], was K = 2 (Fig. 1; see also Fig. S1). A secondary ΔK peak at K = 5 suggests further substructure within the major subpopulations (discussed below). An analysis of molecular variance (AMOVA) indicates that 33% of the total genetic variation is partitioned between the two genetic subpopulations (Table S2). This very high level of differentiation suggests long-term evolutionary divergence between the two subpopulations, with independent origins of cultivated coconuts from within each lineage. Moreover, the two genetic subpopulations are structured geographically and are broadly concordant with the ‘A’ and ‘B’ groups in the GCP/CIRAD classification scheme (Table 1; Fig. 1). Nearly identical patterns to those observed in the *Structure* analysis are found using *InStruct* [26], a similar Bayesian analysis that relaxes assumptions of random mating within subpopulations (Fig. S2). Taken together, these patterns strongly suggest independent domestication events in the Pacific and Indian Ocean basins.

#### Human migration and coconut admixture in the Indian Ocean

Historical records suggest that 14–16 centuries ago, Austronesians and Arabs were trading along the oceanic route connecting Southeast Asia to southern coastal east Africa [27]. This route spanned both Pacific and Indian Ocean coconut subpopulations and therefore could have served as an avenue of introgression of Pacific coconuts into the Indian Ocean. The trade route included Comoros and Madagascar, but not the Seychelles, which were among the last islands in the Indian Ocean to be inhabited [8]. Population membership coefficients in our *Structure* analysis support the hypothesis of Pacific coconut introgression specifically along the ancient trade route. For coconuts outside of this region (populations 1–15, 19; Table 1), evidence of admixture between the two subpopulations is minimal; >96% of accessions can be assigned unambiguously to either the Pacific or Indian Ocean subpopulation with membership coefficient values of Q≥80% (Fig. 1; Table S1). In contrast, for coconuts from the Comoros and Madagascar (populations 17–18), fewer than one-third of accessions are assigned to the Pacific or Indian Ocean subpopulation at Q≥80%. Similarly, in nearby East Africa (population 16), 23% of accessions show ambiguous assignment (Q<80%). Membership coefficient values assigned at the level of population groupings are also consistent with these patterns of admixture (Table 1).

Introgression from Pacific coconuts into the western Indian Ocean is further reflected in the distributions of individual microsatellite alleles whose frequencies differ between the two major subpopulations and which can therefore serve as subpopulation-diagnostic markers. We identified six such alleles using Shannon's mutual information index (see Methods). Their distributions are very similar across the Indian Ocean, with high coefficients of determination that corroborate the scenario of Pacific coconut admixture (mean R^2^ = 0.866). To explicitly evaluate the relative contributions of the two subpopulations to the genomes of the putative admixed populations, we calculated a composite introgression index (*T_i_*; Table 2; see Methods). This measure suggests that for Madagascar and Comoros, Southeast Asian admixture accounts for approximately one-half of the genetic variation present in these regions (*T_i_* = 0.407 and 0.509 for Madagascar and Comoros, respectively; Table 2). For East African collections, the level of inferred introgression falls to approximately one-quarter of the total genetic variation (*T_i_* = 0.254). In the Seychelles, outside the Austronesian trade route, no evidence of introgression is observed (*T_i_* = −0.065≈0).

**Table 2:** Assessments of introgression from Southeast Asian coconuts into western Indian Ocean populations^a^.

		Allele frequency						
Allele	Sh	A3	B1	B2	COM	MAD	SEY	R^2^
CnCirA3_228_	0.715	0.072	0.97	0.68	0.35	0.424	0.75	0.848
CnCirC12_167_	0.631	0.006	0.834	0.614	0.375	0.465	0.771	0.971
CnCirE12_174_	0.604	0.023	0.85	0.541	0.545	0.394	0.856	0.741
CnCirF2_193_	0.390	0.025	0.67	0.674	0.654	0.625	0.95	0.863
CnCirE10_244_	0.389	0.081	0.767	0.514	0.375	0.512	0.922	0.934
CnCirC7_157_	0.378	0.662	0.027	0.155	0.563	0.279	0	0.839
**Mean introgression index (** ***T_i_*** **)**		**1.000**	**0.000**	**0.254**	**0.509**	**0.407**	**−0.065**	**0.866**

a Shannon's mutual information index (Sh), frequencies of six subpopulation-diagnostic microsatellite alleles by population grouping, coefficients of determination (R^2^), and mean introgression index values (*T_i_*). Population groups correspond to Table 1. The introgression model assumes admixture between group A3 (Southeast Asia, populations 4–6) and group B1 (Indo-Atlantic, population 15).

### Regional population structure

**Figure 2:**
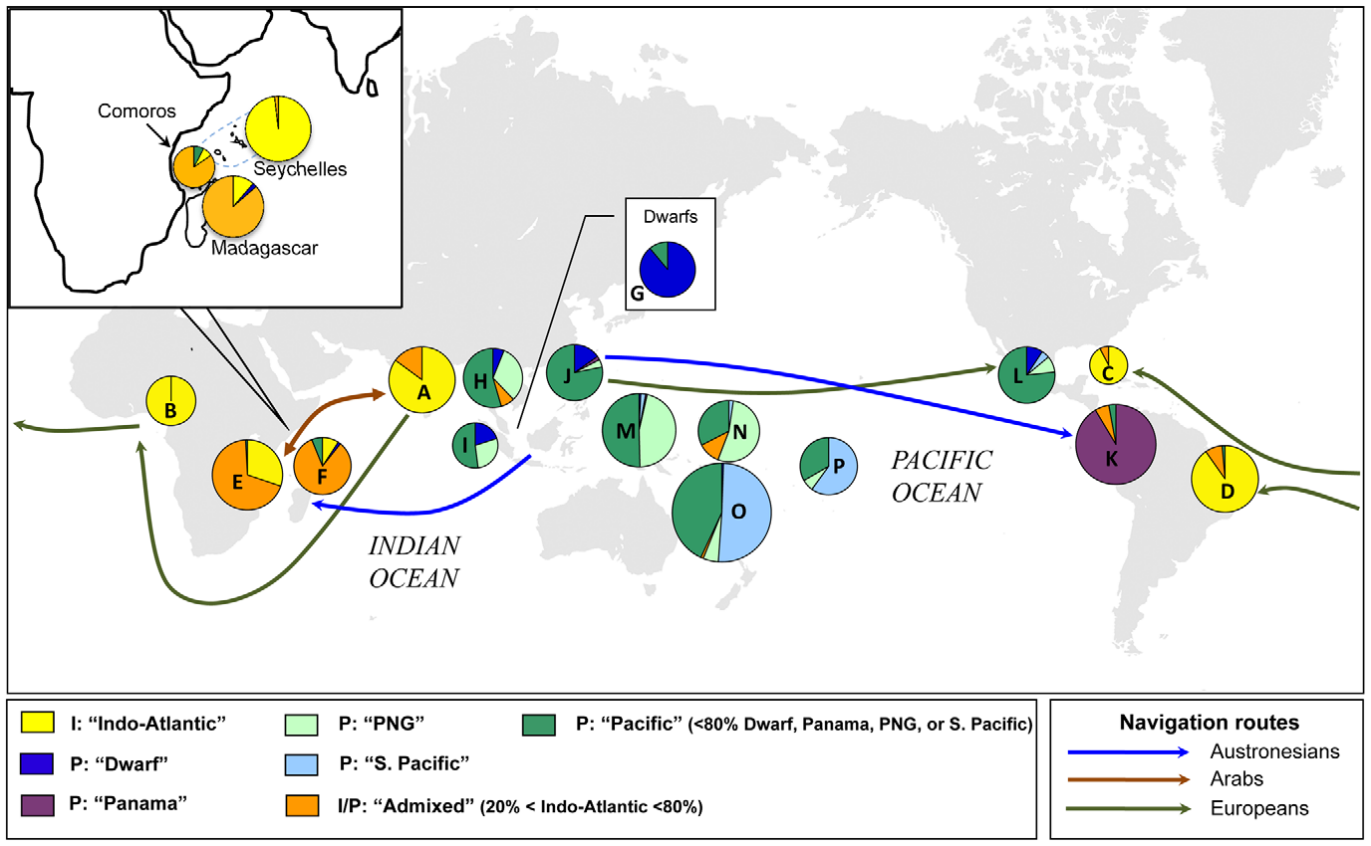
Geographical distributions of Indo-Atlantic and Pacific coconut subpopulations. Subpopulation designations correspond to assignments at Q≥80% membership in *Structure* analyses at K = 5. ‘I’ and ‘P’ prefixes in the legend indicate ‘Indo-Atlantic’ and ‘Pacific’ population assignments at K = 2 assumed populations (≥80% membership; see Fig. 1). Lines indicate proposed coconut dispersal routes by humans. Pie chart labels correspond to the following countries (ISO abbreviations) and sample sizes: A = IND, LKA, SEY (114); B = BEN, CIV, CMR, GHA (29); C = JAM, MEX (Atlantic) (13); D = BRA (72); E = KEN, MOZ, TZA (116); F = MAD, COM (65); G = Dwarf (54); H = CHN, KHM, MYS, THD, VNM (66); I = IDN (25); J = PHL (46); K = PAN (105); L = MEX (Pacific) (43); M = PNG (141); N = KIT, MHL, TUV (43); O = NCL, SLB, VUT (360); P = COK, FJI, PYF (30). Inset: subpopulation compositions for Madagascar, Comoros, and Seychelles. Pie chart composition is selected to reflect geographical population structure and does not correspond directly to GPC/CIRAD designations in Table 1.

The presence of a secondary peak of the ΔK *ad hoc* statistic (Fig. S1) prompted us to perform an analysis with K = 5. It revealed substructure that preserves the integrity of the Indo-Atlantic lineage but divides the Pacific group into four components, referred to here as Panama, Dwarf, Papua New Guinea (PNG) and South Pacific (Fig. 2). These names refer to the region (or coconut type) where they predominate, although most components span multiple regions, as described below.

Table 3a presents pairwise distances calculated in *Structure* (above diagonal) and Jost's [28] relative differentiation (*D*) (below diagonal) for these five subpopulations. Both measures highlight the genetic isolation of the Indian Ocean from the Pacific populations, consistent with long-term evolutionary divergence between the two lineages. The main interest of Jost's measure is that differentiation and diversity represent structurally independent between- and within-population diversity components. As a result, the range of variation of *D* between the Indian and Pacific populations (0.800–0890) is much narrower than in the distances (0.365–0.566), which are, by construction, correlated with heterozygosity (see Table 3b). Jost's *D* is also related to Nei's distance measure (*D_Nei_* = −ln(1−*D*) [29]), which yields values ranging from 1.60 to 2.21 between Indo-Atlantic and Pacific populations. These values are 3.2–4.4 times greater than the largest value between Pacific components (0.504 between Panama and South Pacific), further illustrating that Indo-Atlantic and Pacific coconuts diverged from each other long before any divergence within the Pacific.

**Table 3:** Distances (D*_A,B_*), differentiation (*D*) and diversity parameters for populations identified by *Structure* a ^,^ b.

	Indo-Atlantic (IA)	Panama(PAN)	Dwarf(DW)	PNG	S. Pacific(SP)
**a)**					
**IA**	—	0.566	0.469	0.365	0.377
**PAN**	0.890	—	0.221	0.202	0.221
**DW**	0.878	0.348	—	0.101	0.129
**PNG**	0.800	0.363	0.221	—	0.032
**SP**	0.824	0.396	0.283	0.085	—
**b)**					
***H***	0.464	0.264	0.468	0.623	0.620
***J***	0.536	0.736	0.532	0.377	0.380
**Δ**	1.866	1.358	1.878	2.655	2.635

a) pairwise distances (above diagonal) and differentiation measures (*D*, below diagonal) between populations;

b) expected proportions of homozygotes (*J*), heterozygotes (*H*), and diversity (Δ).

To assess the geographical distribution of the five population components, we assigned accessions to one of seven categories based on population membership coefficients at K = 5: accessions with membership coefficients of Q>80% were assigned to each of the five subpopulations (Indo-Atlantic, Dwarfs, Panama, Papua New Guinea, South Pacific); those with 20–80% Indo-Atlantic membership were defined as ‘admixed’; and remaining accessions (i.e., those with <20% Indo-Atlantic membership and with <80% membership in any single Pacific subpopulation) were assigned to a generic ‘Pacific’ class. Figure 2 shows the worldwide geographical distributions of these seven categories. In the descriptions below, letters in parentheses correspond to pie chart labels in Figure 2.

#### South Asia, Africa and the Caribbean

As is observed at K = 2, the Indian Ocean component predominates in South Asia and the Seychelles (A), as well as in West Africa (B), the Caribbean (C) and Brazil (D) (Fig. 2). Historical records indicate that coconut was unknown in the Caribbean and Atlantic basins until after European colonization [8]; the low level of Pacific admixture in these regions shows that these introductions did not involve admixed populations such as those found today in East Africa (E) or in the western Indian Ocean (F) (Figs. 1, 2). In the admixed populations (E, F), approximately 75% of the Pacific contribution can be assigned to the ‘Dwarf’ and ‘Pacific’ population components, consistent with Austronesian introductions from island Southeast Asia (see above; Table S1).

#### Southeast Asia and Pacific Neotropics

Admixture from the Indo-Atlantic subpopulation is evident at a low frequency in the Pacific coconuts of continental Southeast Asia (H), especially in Thailand, Malaysia, and Cambodia (Fig. 2; Table S1). This pattern may reflect the geographical proximity of these regions to eastern Indian Ocean populations (e.g., Andamans), or longer-distance trading with South Asia (see, e.g., [30]). Interestingly, the ‘Dwarf’ population component, characteristic of self-pollinating Dwarf cultivars (G), is shared with Talls of Southeast Asia (H, I and J). Previous analyses have suggested that the Dwarf varieties originated the Pacific (e.g., [5]). The present data strongly suggest an origin for these varieties specifically in Southeast Asia.

Pacific coast ‘Panama Tall’ coconuts (K) are characterized predominantly by the ‘Panama’ population component. This component is absent elsewhere, except in the Philippines (J) where it occurs at a low frequency (Fig. 2; Table S1). This pattern is consistent with the previously proposed origin of these varieties through a prehistoric introduction from the Philippines [7]. In contrast, the Pacific coast of Mexico (L), which was also populated largely by Philippine coconuts — but in post-colonial times and through multiple introductions [2] — shows a genetic composition that more closely reflects the genetic heterogeneity of the Philippines (Fig. 2). The small contribution of the ‘South Pacific’ component in Mexico may reflect early Spanish importations from the Solomon Islands [2].

#### South Pacific

In Papua New Guinea (M) and in Micronesia (N), the ‘PNG’ population component predominates. The apparent presence of Indo-Atlantic admixture in Micronesia (N, Fig. 2) may reflect European introductions from South Asia during the period when both regions were under British administration; the shared occurrence of similar green-fruited Dwarf varieties in Sri Lanka and Micronesia (Table S1) is consistent with this hypothesis. To the south and east of Micronesia, the proportion of the ‘South Pacific’ population component increases. Coconuts in Melanesia (O) are of similar genetic composition to those from Polynesia (P). More than 50% of the individuals in these regions are predominantly of the ‘South Pacific’ component (Table S1). This includes an outcrossing, compact-growth variety, *‘niu leka’* (‘Fiji Dwarf’), which represents an independent origin of the dwarf habit, distinct from the widely-cultivated self-pollinating Dwarfs of Southeast Asian origin (Tables 1, S1).

## Discussion

### Independent domestications of Pacific and Indo-Atlantic coconuts

A striking observation from our worldwide analysis of coconuts is the high level of genetic differentiation between Pacific and Indian Ocean samples (Table 1, Fig. 1; Fig. S2); 33% of the total observed variation is partitioned between the two genetic subpopulations corresponding to the two ocean basins. This finding has several important implications for coconut domestication. First, it makes it clear that *Cocos nucifera* is a native species of both the Indian and Pacific Oceans, with a long-standing evolutionary presence in both ocean basins. Fossil data from the Palaeocene also support the long-term presence of coconuts (or coconut-like species) in both the Indian and Pacific basins [31], [32].

In addition, the clear genetic differentiation between the Pacific and Indian Ocean lineages allows us to conclude definitively that coconuts were brought into cultivation independently in each of these regions. In the Pacific, the phenotypic diversity and population heterogeneity associated with a region extending from the Malay peninsula to New Guinea (Table 1, Fig. 2) point to that area as a likely center of domestication. This region (‘Malesia’) was earlier claimed as the center of domestication for coconut [33]. Island Southeast Asia has also recently been identified as one of several centers of domestication for swine [34], an indication that this was likely an active area of agricultural development. For Indian Ocean coconuts, archaeological and archaeobotanical findings (coconut shells and sennit rope) from Arikamedu (near Pondicherry) [35], together with Proto-South Dravidian linguistic evidence [36] and ancient Ayurvedic texts [37] suggest that coconuts were already in cultivation in the southern Indian subcontinent around 2,500–3,000 years ago. Our genetic data, when taken together with these other lines of evidence (see also Supporting Information, Text S1; Table S4), suggest that the region encompassing the southern periphery of India, including Sri Lanka, Maldives, and Laccadives, represents a likely center of coconut domestication. These two proposed centers of origin are consistent with those proposed in the 1930s by Vavilov, who also envisioned two centers of origin, one in India and one in the region spanning Indo-China and the Malay archipelago [38].

Interestingly, these two domestication events are associated with markedly different patterns of phenotypic diversification and population substructure. The Indo-Atlantic group shows only moderate gene diversity (Table 1), it is adequately represented by a single genetic subpopulation (Fig. 2), it comprises only the Tall growth form, and its fruit is almost exclusively the elongated (and presumed ancestral) ‘*niu kafa*’ type. This group also remained confined within the Indian Ocean basin until the European colonial era. In contrast, the Pacific group has higher levels of gene diversity (Table 1), it shows evidence of genetic heterogeneity and population substructure that are correlated with its wide geographical distribution (Fig. 2), and it is phenotypically diverse. Pacific coconuts include Talls but are also the source of the widely disseminated, self-pollinating Dwarfs, which our data suggest originated in Southeast Asia (Fig. 2). An additional compact-growth form, the outcrossing Polynesian ‘*niu leka*’ (‘Fiji Dwarf’) variety, also arose in the Pacific group (Table 1; Table S1). While the Pacific coconut fruit is predominantly of the round ‘*niu vai*’ type, the ‘*niu kafa*’ form is also present, including in Samoa where these names originate. Moreover, unlike the geographically limited Indian Ocean coconuts, Pacific coconuts had become widely distributed throughout the Pacific basin, including the New World tropics, before any European contact. Thus, there is a fundamental asymmetry in the genetic heterogeneity, phenotypic diversity, and regional and global impacts of these two domestication events.

### Genetic impacts of coconut dispersal by humans

The genetic distinctness of the Indo-Atlantic and Pacific coconut lineages facilitates our ability to track the genetic footprints of human introductions around the world. Most striking is the genetic admixture in the western Indian Ocean reflecting Pacific coconut introgression. Our analyses suggest that admixed coconuts predominate in the region corresponding to the ancient Austronesian trade route connecting Southeast Asia to Madagascar and coastal east Africa; in contrast, no admixture is evident in the more northerly Seychelles, which fall outside the trade route (Table 2; Fig. 2). The influence of Austronesians along this corridor is well documented [39], perhaps most notably in its lasting impact on human population structure (e.g., [40]). Interestingly, like coconut, a recent study of rice in Madagascar also indicates a shared role for crop varieties originating from Southeast Asia (*japonica* rice) and the Indian subcontinent (*indica* rice), with admixture in Madagascar [41].

Admixture between Pacific and the Indian Ocean coconuts was likely further promoted by the later presence of Arabo-Persian merchants who regularly visited East Africa, trading coconut and favoring its cultivation [42]. Archaeobotanical sources from Pemba [43] show the importance of coconuts from 700–1500 CE in the food culture influenced by Islamic traders in the Indian Ocean. This dual dissemination of the coconut in the Indian Ocean, first by Austronesians and later by South Asians and Arabs, has been well captured linguistically by Allibert [27]: “I have been able to follow the diffusion of the coconut palm from the East to the West, through the Austronesian terms *buahniu* (Bali)/*voanio* (Madagascar), not to mention *vanu* in the Loyalty Islands, but also from *narikela* (Sanskrit)/*nargil* (Arabic, Persian)/*mnazi* (Bantu), a double linguistic pathway for the same tree, the one directly across the Indian Ocean, the other via the north of the same ocean.” Recent observations of genetically admixed coconut populations in Oman [44] further support this dissemination history.

Within the Pacific basin, human influence on coconut population structure is most readily detectable in the pre-historic introduction of Southeast Asian coconuts to the New World coast. This introduction is estimated to have occurred ∼2,250 years ago, and our analyses are consistent with previous findings suggesting a Philippine origin (Fig. 2; ref [7]); the low genetic diversity in Panama Talls provides further evidence of establishment through a founder event (Table 1). Later European influences are apparent in the Spanish establishment of Mexican populations (see ref [2]); the clear Pacific composition of these coconuts stands in marked contrast to European introductions into the Caribbean and Atlantic basins, which appear to be of Indian origin (Figs. 1, 2; Fig. S2; Table S1). Historical records confirm that the Portuguese established coconut plantations in West Africa, Brazil, and later the Caribbean after Vasco da Gama's 1498 expedition to the Indian Ocean [8]. In the Old World portion of the Pacific basin, our analyses reveal geographical substructure in a pattern that could plausibly reflect human dispersal of coconuts out of the proposed Southeast Asian center of domestication (H, I, J; Fig. 2) and south and east towards Polynesia (M and N; Fig. 2) (see also discussion in ref [45]).

### Conclusions

In the most extensive genetic analysis of coconuts to date, we find evidence for independent origins of coconut cultivation in the Pacific and Indian Ocean basins. Interestingly, despite the long-term, extensive movement of coconuts by humans both within and between these oceanic basins, most contemporary coconuts do not show evidence of substantial genetic admixture between the two major genetic subpopulations (Fig. 1; Fig. S2). Given the absence of any known reproductive isolating barriers, the high level of genetic differentiation between these subpopulations suggests a long period of isolation prior to human influence. In this light, the predominance of genetic admixture in the western Indian Ocean (Figs. 1, 2; Tables 1, 3) suggests that humans likely played a prominent role in the establishment and propagation of coconuts in that region.

Besides revealing basic insights into the cultivation and dispersal history of this iconic tropical species, our findings may also facilitate efforts to protect the viability of the coconut as a crop species. Coconut lethal yellowing, a phytoplasma infection, has reached epidemic levels in the Caribbean and other regions of the Neotropics; susceptible trees typically succumb within a year of infection. Knowledge of the worldwide genetic structure of the coconut, including regions where genetic admixture has generated augmented levels of genetic diversity (e.g., Madagascar; Table 1), may ultimately prove useful in targeting source populations for disease resistance and other crop improvement traits.

## Materials and Methods

GCP/CIRAD accessions correspond to those in the GCP database (http://gcpcr.grinfo.net/index.php); growth form, variety name, source country, and germplasm group assignment are indicated in Table S1. An additional 112 coconut palms were sampled from populations occurring on the islands of Madagascar, Comoros and Seychelles. Portions of emerging leaf fronds were collected from the crowns of trees; tissue samples were dried in silica gel desiccant for DNA extraction. Voucher herbarium specimens for the Indian Ocean collections are housed at the Missouri Botanical Garden (MO). Sampled accessions represent 11 locations on Madagascar, 5 on Comoros, and 6 on the Seychelles (Table S1). Genomic DNA was extracted using DNeasy Kits (Qiagen, Valencia, CA) at Washington University.

Genetic analyses were performed using ten microsatellite loci (CnCrF2, CnCrC12, CnCrE10, CnCrA9, CnCrC7, CnCrB6, CnCrE12, CnCrA3, CnCrG11 and CnCrH7). Genotyping of the GCP/CIRAD collection is described in ref [20]. For Indian Ocean accessions, PCR amplifications were performed using similar conditions, and products were separated on an ABI Prism 3130 genetic analyzer at Washington University. Control DNAs with known allele lengths were amplified for all ten loci to standardize scoring of allele sizes. Data were collected and assembled with Genotyper 2.5 software (Perkin Elmer Biosystems).

### Genetic Analyses

Analyses of genetic diversity and AMOVA were performed with *GENALEX 6* [46]. To investigate population structure we used Bayesian clustering methods as implemented in *Structure* 2.3 [24] and *InStruct* [26]. *InStruct* is similar to *Structure* but relaxes assumptions of Hardy-Weinberg equilibrium within subpopulations. For *Structure* analyses, the number of subpopulations, K, was set at values ranging from 1–10, with 20 replicate runs apiece (100,000 burnin, 1,000,000 runs). An admixture ancestry model was selected with allele frequencies correlated. For the optimal inferred K value (K = 2), we employed CLUMPP version 1.1.2 [47] to confirm the similarity of clustering memberships among multiple *Structure* runs (the maximum H′ value was >0.9995 at the optimal inferred K value). *InStruct* analyses were performed using the Cornell University BioHPC web portal (http://cbsuapps.tc.cornell.edu/InStruct.aspx). The program *DISTRUCT* [48] was used to visualize outputs from *CLUMPP* and *InStruct* analyses.

Because Dwarf accessions are highly homozygous and show little genetic diversity, clustering analyses were performed both with and without Dwarfs to test for potential artifacts created by their inclusion; excluding these accessions did not substantially alter inferences. In additional analyses, we applied explicit spatial clustering as implemented in *BAPS* [49] and *GENELAND* [50]. However, results were highly biased towards sampling location, a reflection of the pan-global distribution of our dataset, and were not included in further analysis.

### Introgression index

To test for Pacific introgression into the Indian Ocean populations, we defined ‘diagnostic alleles,’ i.e., alleles that are differentially represented in GCP/CIRAD subgroup A3 (a representative Pacific subgroup) relative to subgroup B1 (representative Indo-Atlantic), and we selected them using Shannon's mutual information [51], [52] (Table S3). We calculated the entropy of allele *a* in population A3 as a function of *p_aA_*, its frequency in population A3: *h_A_*(*a*) = −*p_aA_*log *p_aA_*−(1−*p_aA_*)log(1−*p_aA_*). Likewise, we calculated *h_B_*(*a*) based on *p_aB_* its frequency in population B1 and *h_T_*(*a*), based on *p_aT_* = ½(*p_aA_*+*p_aB_*). The mutual information quantity between *a* (the allele) and *G* (the group) is thus *I*(*a;G*) = *h_T_*(*a*)−½[*h_A_*(*a*)+*h_B_*(*a*)]. Expressed in Shannon units (Sh, using base 2 logarithms), the mutual information quantity may range from 0 (same frequencies in A3 and B1) to 1 (the allele is specific to one population). We retained alleles corresponding to the six top values.

Based on the frequencies of these alleles in six groups (A3, B1, B2, Madagascar, Comoros, and Seychelles), we then calculated ‘introgression indices’ for each allele: *T_ia_* = (*Z_ia_*−*X_a_*)/(*Y_a_*−*X_a_*) where *X_a_*, *Y_a_*, and *Z_ia_* are the respective frequencies in B1, A3, and the four other groups. Indices *i* and *a* refer to group and allele, respectively. The mean of the index over all alleles (*T_i_*) is an estimation of the percentage of alleles from Southeast Asia in each group. Finally, we assessed the consistency of the introgression model by calculating the coefficient of determination R^2^ of the regression of the frequencies of each allele on *T_i_* (excluding groups B1 and A3).

### Differentiation measures

Jost [29] shows that Nei's heterozygosity (*H*) and the associated *G_ST_* are not adequate measures of diversity and differentiation, respectively. He suggests instead using the reciprocal of Nei's identity as a measure of diversity, and he derives absolute and relative measures of differentiation. These measures are, respectively, Δ*_ST_* = Δ*_T_*/Δ*_S_* = J*_S_*/J*_T_* and *D* = (*J_T_*/*J_S_* −1)/[(1/*n*)−1]. In these formulae, *J* = 1−*F* refers to Nei's identity and is the expected proportion of homozygotes in a population. *J_S_* is the average of Nei's identities in the sub-populations. The within-population component of diversity is Δ*_S_* = 1/*J_S_*.The total diversity is Δ*_T_* = 1/*J_T_* where *J_T_* is calculated based on the allele frequencies in the pooled population. We derived these parameters from the *Structure* outputs (heterozygosities and distances).

## Supporting Information

Figure S1 Assessment of subpopulation number in *Structure* analyses.
(DOC)

Figure S2 *InStruct* output at K = 2 subpopulations.
(DOC)

Table S1 Information on coconut accessions used in analyses and assignment probabilities at K = 2 and K = 5 using *Structure* analysis.
(DOC)

Table S2 Allele frequencies for each locus for Pacific and western Indian Ocean populations.
(DOC)

Table S3 Analysis of Molecular Variance (AMOVA) for all coconut accessions (1322 individuals).
(DOC)

Table S4 Language roots associated with the coconut in proto-South-Dravidian and proto-Telugu.
(DOC)

Text S1 Early evidence of coconut use in the southern Indian subcontinent and neighboring islands.
(DOC)

## References

[1] Harries HC (1978). The evolution, dissemination and classification of *Cocos nucifera*. L.. Bot Rev.

[2] Zizumbo-Villarreal D (1996). History of coconut (*Cocos nucifera* L.) in Mexico: 1539–1810.. Genet Resour Crop Evol.

[3] Batugal P, Rao V, Oliver J (2005). Coconut Genetic Resources..

[4] Ward G, Brookfield M (1992). The dispersal of the coconut: did it float or was it carried to Panama?. J Biogeogr.

[5] Lebrun P, N'Cho Y, Bourdeix R, Baudouin L, Hamon P, Seguin M, Perrier X, Glaszmann JC (2003). Coconut.. Genetic diversity of cultivated tropical plants.

[6] Harries HC, Baudouin L, Cardena R (2004). Floating, boating and introgression: Molecular techniques and ancestry of the coconut palm populations on Pacific islands.. Ethnobot Res and App.

[7] Baudouin L, Lebrun P (2009). Coconut (*Cocos nucifera* L.) DNA studies support the hypothesis of an ancient Austronesian migration from Southeast Asia to America.. Genet Resour Crop Evol.

[8] Sauer JD, Riley C (1971). A re-evaluation of the coconut as an indicator of human dispersal.. Man across the sea.

[9] Whitehead RA (1966). Sample survey and collection of coconut germplasm in the Pacific islands (30 May–5 September 1964).

[10] Harries H (1981). Germination and taxonomy of the coconut.. Ann Bot.

[11] Bourdeix R, Baudouin L, Billotte N, Labouisse J, Noiret J, André C, Michel J, Serge H, Dominique N (2001). Tropical plant breeding.. Tropical plant breeding.

[12] Mauro-Herrera M, Meerow A, Perera L, Russell JR, Schnell RJ (2010). Ambiguous genetic relationships among coconut (*Cocos nucifera* L.) cultivars: the effects of outcrossing, sample source and size, and method of analysis.. Genet Res and Crop Evol.

[13] Meerow A, Noblick L, Borrone JW, Couvreur TL, Mauro-Herrera M (2009). Phylogenetic analysis of seven WRKY genes across the palm subtribe Attaleinae (Arecaceae) identifies *Syagrus* as sister group of the coconut.. PLoS ONE.

[14] Lebrun P, N'Cho YP, Seguin M, Grivet L, Baudouin L (1998). Genetic diversity in the coconut (*Cocos nucifera* L.) revealed by restriction fragment length polymorphism (RFLP) markers.. Euphytica.

[15] Perera L, Russell JR, Provan J, Powell W (1999). Identification and characterization of microsatellite loci in coconut (*Cocos nucifera* L.) and the analysis of coconut populations in Sri Lanka.. Mol Ecol.

[16] Rivera R, Edwards KJ, Barker JH, Arnold GM, Ayad G (1999). Isolation and characterization of polymorphic microsatellites in *Cocos nucifera* L.. Genome.

[17] Teulat B, Aldam C, Trehin R, Lebrun P, Barker JHA (2000). An analysis of genetic diversity of the coconut (*Cocos nucifera*) populations from across the geographic range using sequence-tagged microsatellites (SSRs) and AFLPs.. Theor Appl Genet.

[18] Perera L, Russell JR, Provan J, Powell W (2003). Studying genetic relationships among coconut varieties/populations using microsatellite markers.. Euphytica.

[19] Baudouin L, Lebrun P, Rognon F, Ritter E, Batugal P, Rao VR, Oliver J (2005). Use of molecular markers for coconut improvement.. Coconut Genetic Resources.

[20] Baudouin L, Lebrun P (2002). The development of a microsatellite kit for use with coconuts.

[21] Rajesh MK, Arunachalam V, Nagarajan P, Lebrun P, Samsudeen K (2008). Genetic survey of 10 Indian coconut landraces by simple sequence repeats (SSRs).. Sci Hortic.

[22] Martinez R, Baudouin L, Berger A, Dollet M (2010). Characterization of the genetic diversity of the tall coconut (*Cocos nucifera* L.) population by molecular markers microsatellite (SSR) types in the Dominican Republic.. Tree Genet Genom.

[23] Baudouin L, Lebrun P, Berger A, Myrie W, Been B (2008). The Panama Tall and the Maypan hybrid coconut in Jamaica: Did genetic contamination cause a loss of resistance to Lethal Yellowing?. Euphytica.

[24] Pritchard JK, Stephens M, Donnelly P (2000). Inference of population structure using multilocus genotype data.. Genetics.

[25] Evanno G, Renaut S, Goudet J (2005). Detecting the number of clusters of individuals using the software STRUCTURE: a simulation study.. Mol Ecol.

[26] Gao H, Williamson H, Bustamante CD (2007). An MCMC approach for joint inference of population structure and inbreeding rates from multi-locus genotype data.. Genetics.

[27] Allibert C (2008). Austronesian migration and the establishment of the Malagasy civilisation: Contrasted readings in linguistics, archaeology, genetics and cultural anthropology.. Diogenes.

[28] Jost L (2008). G_ST_ and its relatives do not measure differentiation.. Mol Ecol.

[29] Jost L (2009). D vs. G_ST_: Response to Heller and Siegismund (2009) and Ryman and Leimar (2009).. Mol Ecol.

[30] Cooper Z (2002). Archeology and history: early settlements in the Andaman Islands.

[31] Rigby JF, Pant DD (1995). A fossil *Cocos nucifera* L. fruit from the latest Pliocene of Queensland, Australia.. Global environment and diversification of plants through geological time, Society of Indian Plant Taxonomists, Allahabad University, Allahabad, India: Birbal Sahni Institute, Centennial Volume.

[32] Tripathi RP, Mishra SN, Sharma BD (1999). *Cocos nucifera*-like petrified fruit from the Tertiary of Amarkantak, M.P., India.. Paleobotanist.

[33] Harries HC, Baas P, Kalkman K, Geesink R (1990). Malesian origin for a domestic *Cocos nucifera*.. The Plant Diversity of Malesia.

[34] Larson G, Dobney K, Albarella U, Fang M, Matisoo-Smith E (2005). Worldwide phyloeography of wild boar reveals multiple centers of pig domestication.. Science.

[35] Wheeler REM, Ghosh A, Deva K (1946). Arikamedu: an Indo-Roman trading station on east coast of India.. Ancient India: Bulletin of the Archaeological Survey of India.

[36] Fuller D, Petraglia MD, Allchin B (2007). Non-human genetics, agricultural origins and historical linguistics in South Asia.. The evolution and history of human populations in South Asia.

[37] Menon KP, Pandalai KM (1958). The coconut palm, a monograph.

[38] Chester K (1951). The origin, variation, immunity and breeding of cultivated plants. Selected writings of N. I. Valvilov; trans Chester KS.

[39] Spriggs M (1997). The Island Melanesians.

[40] Hurles M, Skyes B, Jobling M, Forster P (2005). The dual origin of the Malagasy in Island Southeast Asia and East Africa: Evidence from maternal and paternal lineages.. Am J Hum Genet.

[41] Mather KA, Molina J, Flowers JM, Rubinstein S, Rauh BL (2010). Migration, isolation and hybridization in island crop populations: the case of Madagascar rice.. Mol Ecol.

[42] Schuilling M, Harries HC (1994). The coconut in East Africa 1. East African Tall.. Principes.

[43] Walshaw S (2010). Converting to rice: urbanization, Islamization and crops on Pemba Island, Tanzania, AD 700–1500.. World Archaeol.

[44] Perera L, Baudouin L, Bourdeix R, Bait Fadil A, Hountoundji FCC (2011). Coconut palms on the edge of the desert: genetic diversity of *Cocos nucifera* L. in Oman.. CORD.

[45] Hurles M, Matisoo-Smith E, Gray R, Penny D (2003). Untangling Oceanic settlement: the edge of the knowable.. Trends in Ecology and Evolution.

[46] Peakall R, Smouse PE (2006). *GENALEX 6*: genetic analysis in Excel. Population genetic software for teaching and research.. Mol Ecol Notes.

[47] Jakobsson M, Rosenberg NA (2007). *CLUMPP*: a cluster matching and permutation program for dealing with label switching and multimodality in analysis of population structure.. Bioinformatics.

[48] Rosenberg NA (2004). *DISTRUCT*: a program for the graphical display of population structure.. Mol Ecol Notes.

[49] Corander J, Waldmann P, Marttinen P, Sillanpää MJ (2004). BAPS 2: enhanced possibilities for the analysis of genetic population structure.. Bioinformatics.

[50] Guillot G, Estoup A, Mortier F, Cosso J (2005). A Spatial Statistical Model for Landscape Genetics.. Genetics.

[51] Shannon C (1948). A mathematical theory of communication.. Bell System Tech J.

[52] Battail G (1997). Théorie de l'information, Application aux techniques de communication.

