# Supplementary material for: Independent Origins of Cultivated Coconut (Cocos nucifera L.) in the Old World Tropics
Source: PLoS One. 2011 Jun 22;6(6):e21143. doi: 10.1371/journal.pone.0021143 (PMC3120816; doi:10.1371/journal.pone.0021143)
Supplement: Figure S1 — Assessment of subpopulation number in Structure analyses. (DOC) [file pone.0021143.s001.doc]

**Supporting information**

**Figure S1. Assessment of subpopulation number in *Structure* analyses.** Delta-K refers to the *ad hoc* method of Evanno *et al.* (2005) for assessing optimal subpopulation number.
