# Supplementary material for: Independent Origins of Cultivated Coconut (Cocos nucifera L.) in the Old World Tropics
Source: PLoS One. 2011 Jun 22;6(6):e21143. doi: 10.1371/journal.pone.0021143 (PMC3120816; doi:10.1371/journal.pone.0021143)

**Supporting Information**

**Figure S2.** **Results of *InStruct* analysis for a worldwide sample of 1322 coconuts.** Population assignments for each accession are shown at K=2 subpopulations. Numbers along the x-axis correspond to group designations in Table 1. Vertical black lines distinguish the population groups.


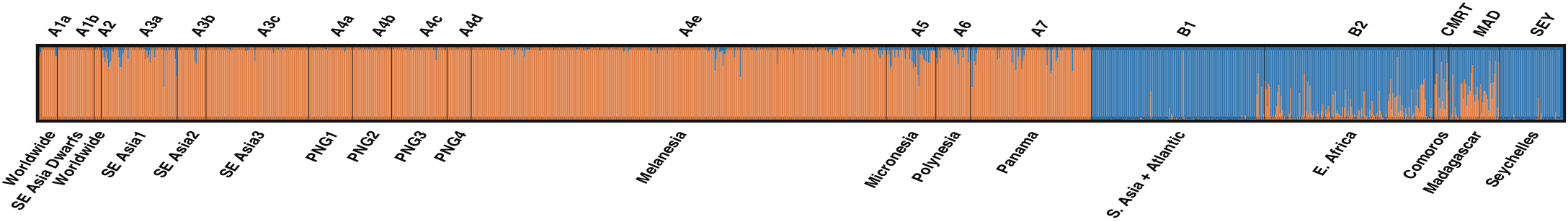

Supplement: Figure S2 — InStruct output at K = 2 subpopulations. (DOC) [file pone.0021143.s002.doc]
