# Supplementary material for: Independent Origins of Cultivated Coconut (Cocos nucifera L.) in the Old World Tropics
Source: PLoS One. 2011 Jun 22;6(6):e21143. doi: 10.1371/journal.pone.0021143 (PMC3120816; doi:10.1371/journal.pone.0021143)
Supplement: Table S1 — Information on coconut accessions used in analyses and assignment probabilities at K = 2 and K = 5 using Structure analysis. (DOC) [file pone.0021143.s003.doc]

**Supporting information**

***Table S1. Information on coconut accessions used in analyses and assignment probabilities with K=2 and K=5 using*** Structure.

| **Accession no.** | **Population (Group)** | **Growth  form** | **Variety name** | **Country of origin** | **K=2** | | **K=5** | | | | |
| --- | --- | --- | --- | --- | --- | --- | --- | --- | --- | --- | --- |
|  |  |  |  |  | **Q_1_** | **Q_2_** | **Q_1_** | **Q_2_** | **Q_3_** | **Q_4_** | **Q_5_** |
| 1 | 1(A1a) | Dwarf | Malayan Green Dwarf | MYS | 0.870 | 0.130 | 0.008 | 0.010 | 0.920 | 0.009 | 0.053 |
| 2 | 1(A1a) | Dwarf | Malayan Green Dwarf | MYS | 0.938 | 0.062 | 0.128 | 0.024 | 0.736 | 0.076 | 0.036 |
| 3 | 1(A1a) | Dwarf | Malayan Red Dwarf | MYS | 0.991 | 0.009 | 0.006 | 0.005 | 0.977 | 0.008 | 0.004 |
| 4 | 1(A1a) | Dwarf | Malayan Yellow Dwarf | MYS | 0.994 | 0.006 | 0.007 | 0.006 | 0.973 | 0.010 | 0.004 |
| 5 | 1(A1a) | Dwarf | Ghana Yellow Dwarf | GHA | 0.994 | 0.006 | 0.008 | 0.006 | 0.973 | 0.010 | 0.004 |
| 6 | 1(A1a) | Dwarf | Kiribati Green Dwarf | KIR | 0.993 | 0.007 | 0.007 | 0.010 | 0.970 | 0.010 | 0.004 |
| 7 | 1(A1a) | Dwarf | Kiribati Green Dwarf | KIR | 0.992 | 0.008 | 0.007 | 0.013 | 0.970 | 0.007 | 0.004 |
| 8 | 1(A1a) | Dwarf | Kiribati Green Dwarf | KIR | 0.993 | 0.007 | 0.007 | 0.013 | 0.969 | 0.007 | 0.004 |
| 9 | 1(A1a) | Dwarf | Madang Brown Dwarf | PNG | 0.992 | 0.008 | 0.006 | 0.005 | 0.978 | 0.007 | 0.004 |
| 10 | 1(A1a) | Dwarf | Madang Brown Dwarf | PNG | 0.992 | 0.008 | 0.006 | 0.005 | 0.978 | 0.007 | 0.004 |
| 11 | 1(A1a) | Dwarf | Sri Lanka Green Dwarf | LKA | 0.992 | 0.008 | 0.008 | 0.011 | 0.967 | 0.010 | 0.004 |
| 12 | 1(A1a) | Dwarf | Ternate Brown Dwarf | IDN | 0.992 | 0.008 | 0.006 | 0.005 | 0.978 | 0.007 | 0.004 |
| 13 | 1(A1a) | Dwarf | Malaysian Red Dwarf | MYS | 0.991 | 0.009 | 0.006 | 0.005 | 0.977 | 0.008 | 0.004 |
| 14 | 1(A1a) | Dwarf | Malayan Green Dwarf | MYS | 0.994 | 0.006 | 0.008 | 0.006 | 0.973 | 0.010 | 0.004 |
| 15 | 1(A1a) | Dwarf | Malayan Green Dwarf | MYS | 0.870 | 0.130 | 0.030 | 0.013 | 0.847 | 0.022 | 0.088 |
| 16 | 1(A1a) | Dwarf | Malayan Green Dwarf | MYS | 0.869 | 0.131 | 0.008 | 0.008 | 0.921 | 0.009 | 0.054 |
| 17 | 2(A1b) | Dwarf | Catigan Green Dwarf | PHL | 0.995 | 0.005 | 0.006 | 0.012 | 0.970 | 0.008 | 0.004 |
| 18 | 2(A1b) | Dwarf | Catigan Green Dwarf | PHL | 0.995 | 0.005 | 0.008 | 0.032 | 0.947 | 0.009 | 0.004 |
| 19 | 2(A1b) | Dwarf | Tacunan Green Dwarf | PHL | 0.995 | 0.005 | 0.007 | 0.014 | 0.966 | 0.009 | 0.003 |
| 20 | 2(A1b) | Dwarf | Tacunan Green Dwarf | PHL | 0.990 | 0.010 | 0.008 | 0.024 | 0.950 | 0.010 | 0.008 |
| 21 | 2(A1b) | Dwarf | Tacunan Green Dwarf | PHL | 0.992 | 0.008 | 0.010 | 0.016 | 0.954 | 0.013 | 0.007 |
| 22 | 2(A1b) | Dwarf | Tacunan Green Dwarf | PHL | 0.995 | 0.005 | 0.007 | 0.015 | 0.964 | 0.010 | 0.003 |
| 23 | 2(A1b) | Dwarf | Tacunan Green Dwarf | PHL | 0.992 | 0.008 | 0.009 | 0.016 | 0.954 | 0.014 | 0.007 |
| 24 | 2(A1b) | Dwarf | Pilipog Green Dwarf | PHL | 0.994 | 0.006 | 0.006 | 0.011 | 0.972 | 0.008 | 0.004 |
| 25 | 2(A1b) | Dwarf | Kapatagan Dwarf | PHL | 0.995 | 0.005 | 0.015 | 0.108 | 0.657 | 0.216 | 0.004 |
| 26 | 2(A1b) | Dwarf | Aromatic Green Dwarf | THA | 0.995 | 0.005 | 0.012 | 0.026 | 0.950 | 0.008 | 0.004 |
| 27 | 2(A1b) | Dwarf | Aromatic Green Dwarf | THA | 0.995 | 0.005 | 0.008 | 0.019 | 0.961 | 0.008 | 0.004 |
| 28 | 2(A1b) | Dwarf | Aromatic Green Dwarf | THA | 0.995 | 0.005 | 0.010 | 0.026 | 0.953 | 0.008 | 0.004 |
| 29 | 2(A1b) | Dwarf | Aromatic Green Dwarf | THA | 0.994 | 0.006 | 0.010 | 0.026 | 0.953 | 0.007 | 0.004 |
| 30 | 2(A1b) | Dwarf | Aromatic Green Dwarf | THA | 0.995 | 0.005 | 0.008 | 0.018 | 0.961 | 0.008 | 0.004 |
| 31 | 2(A1b) | Dwarf | Aromatic Green Dwarf | THA | 0.994 | 0.006 | 0.010 | 0.026 | 0.953 | 0.008 | 0.004 |
| 32 | 2(A1b) | Dwarf | Aromatic Green Dwarf | THA | 0.995 | 0.005 | 0.011 | 0.025 | 0.953 | 0.008 | 0.004 |
| 33 | 2(A1b) | Dwarf | Aromatic Green Dwarf | THA | 0.995 | 0.005 | 0.010 | 0.025 | 0.953 | 0.008 | 0.004 |
| 34 | 2(A1b) | Dwarf | Aromatic Green Dwarf | THA | 0.995 | 0.005 | 0.010 | 0.025 | 0.953 | 0.008 | 0.004 |
| 35 | 2(A1b) | Dwarf | Aromatic Green Dwarf | THA | 0.995 | 0.005 | 0.010 | 0.026 | 0.952 | 0.008 | 0.004 |
| 36 | 2(A1b) | Dwarf | Aromatic Green Dwarf | THA | 0.995 | 0.005 | 0.067 | 0.102 | 0.741 | 0.086 | 0.004 |
| 37 | 2(A1b) | Dwarf | Aromatic Green Dwarf | THA | 0.994 | 0.006 | 0.007 | 0.178 | 0.795 | 0.015 | 0.005 |
| 38 | 2(A1b) | Dwarf | Aromatic Green Dwarf | THA | 0.995 | 0.005 | 0.010 | 0.024 | 0.910 | 0.052 | 0.004 |
| 39 | 2(A1b) | Dwarf | Thailand Green Dwarf | THA | 0.995 | 0.005 | 0.007 | 0.010 | 0.972 | 0.008 | 0.004 |
| 40 | 2(A1b) | Dwarf | Thailand Green Dwarf | THA | 0.994 | 0.006 | 0.009 | 0.012 | 0.966 | 0.009 | 0.004 |
| 41 | 2(A1b) | Dwarf | Thailand Green Dwarf | THA | 0.995 | 0.005 | 0.007 | 0.009 | 0.972 | 0.008 | 0.004 |
| 42 | 2(A1b) | Dwarf | Thailand Green Dwarf | THA | 0.994 | 0.006 | 0.008 | 0.013 | 0.966 | 0.009 | 0.004 |
| 43 | 2(A1b) | Dwarf | Thailand Green Dwarf | THA | 0.994 | 0.006 | 0.010 | 0.018 | 0.955 | 0.012 | 0.004 |
| 44 | 2(A1b) | Dwarf | Thailand Green Dwarf | THA | 0.995 | 0.005 | 0.007 | 0.009 | 0.972 | 0.008 | 0.004 |
| 45 | 2(A1b) | Dwarf | Thailand Green Dwarf | THA | 0.995 | 0.005 | 0.007 | 0.009 | 0.973 | 0.008 | 0.004 |
| 46 | 2(A1b) | Dwarf | Thailand Green Dwarf | THA | 0.995 | 0.005 | 0.007 | 0.009 | 0.972 | 0.008 | 0.004 |
| 47 | 2(A1b) | Dwarf | Thailand Green Dwarf | THA | 0.995 | 0.005 | 0.007 | 0.009 | 0.972 | 0.008 | 0.004 |
| 48 | 2(A1b) | Dwarf | Thailand Green Dwarf | THA | 0.995 | 0.005 | 0.007 | 0.009 | 0.972 | 0.008 | 0.004 |
| 49 | 3(A2) | Dwarf | Cameroon Red Dwarf | CMR | 0.981 | 0.019 | 0.012 | 0.010 | 0.951 | 0.017 | 0.009 |
| 50 | 3(A2) | Dwarf | Cameroon Red Dwarf | CMR | 0.981 | 0.019 | 0.013 | 0.010 | 0.950 | 0.018 | 0.009 |
| 51 | 3(A2) | Dwarf | Vanuatu Red Dwarf | VUT | 0.984 | 0.016 | 0.361 | 0.012 | 0.497 | 0.120 | 0.010 |
| 52 | 3(A2) | Dwarf | Tahiti Red Dwarf | PYF | 0.994 | 0.006 | 0.227 | 0.607 | 0.043 | 0.118 | 0.004 |
| 53 | 3(A2) | Dwarf | Rath Thembili | LKA | 0.985 | 0.015 | 0.023 | 0.006 | 0.833 | 0.131 | 0.007 |
| 54 | 3(A2) | Dwarf | Rath Thembili | LKA | 0.985 | 0.015 | 0.023 | 0.006 | 0.841 | 0.123 | 0.008 |
| 55 | 4(A3a) | Tall | Malayan Tall | MYS | 0.994 | 0.006 | 0.142 | 0.011 | 0.407 | 0.436 | 0.005 |
| 56 | 4(A3a) | Tall | Malayan Tall | MYS | 0.949 | 0.051 | 0.017 | 0.018 | 0.419 | 0.527 | 0.019 |
| 57 | 4(A3a) | Tall | Malayan Tall | MYS | 0.863 | 0.137 | 0.040 | 0.006 | 0.013 | 0.887 | 0.054 |
| 58 | 4(A3a) | Tall | Malayan Tall | MYS | 0.793 | 0.207 | 0.015 | 0.019 | 0.010 | 0.823 | 0.133 |
| 59 | 4(A3a) | Tall | Malayan Tall | MYS | 0.930 | 0.070 | 0.012 | 0.022 | 0.037 | 0.912 | 0.016 |
| 60 | 4(A3a) | Tall | Malayan Tall | MYS | 0.845 | 0.155 | 0.022 | 0.012 | 0.102 | 0.757 | 0.108 |
| 61 | 4(A3a) | Tall | Malayan Tall | MYS | 0.724 | 0.276 | 0.017 | 0.014 | 0.014 | 0.721 | 0.233 |
| 62 | 4(A3a) | Tall | Malayan Tall | MYS | 0.777 | 0.223 | 0.035 | 0.008 | 0.017 | 0.761 | 0.178 |
| 63 | 4(A3a) | Tall | Malayan Tall | MYS | 0.802 | 0.198 | 0.264 | 0.018 | 0.017 | 0.534 | 0.167 |
| 64 | 4(A3a) | Tall | Malayan Tall | MYS | 0.995 | 0.005 | 0.013 | 0.147 | 0.045 | 0.791 | 0.004 |
| 65 | 4(A3a) | Tall | Cambodia Tall Koh Rong | KHM | 0.932 | 0.068 | 0.041 | 0.013 | 0.066 | 0.860 | 0.019 |
| 66 | 4(A3a) | Tall | Cambodia Tall Koh Rong | KHM | 0.993 | 0.007 | 0.024 | 0.026 | 0.371 | 0.572 | 0.006 |
| 67 | 4(A3a) | Tall | Cambodia Tall Koh Rong | KHM | 0.995 | 0.005 | 0.044 | 0.013 | 0.230 | 0.709 | 0.004 |
| 68 | 4(A3a) | Tall | Cambodia Tall Koh Rong | KHM | 0.995 | 0.005 | 0.053 | 0.045 | 0.283 | 0.615 | 0.004 |
| 69 | 4(A3a) | Tall | Cambodia Tall Koh Rong | KHM | 0.995 | 0.005 | 0.030 | 0.009 | 0.039 | 0.918 | 0.004 |
| 70 | 4(A3a) | Tall | Cambodia Tall Ream | KHM | 0.857 | 0.143 | 0.118 | 0.013 | 0.138 | 0.657 | 0.075 |
| 71 | 4(A3a) | Tall | Cambodia Tall Ream | KHM | 0.718 | 0.282 | 0.020 | 0.076 | 0.131 | 0.537 | 0.237 |
| 72 | 4(A3a) | Tall | Cambodia Tall Ream | KHM | 0.725 | 0.275 | 0.069 | 0.027 | 0.083 | 0.563 | 0.258 |
| 73 | 4(A3a) | Tall | Cambodia Tall Ream | KHM | 0.885 | 0.115 | 0.073 | 0.017 | 0.080 | 0.734 | 0.095 |
| 74 | 4(A3a) | Tall | Cambodia Tall Ream | KHM | 0.922 | 0.078 | 0.040 | 0.017 | 0.245 | 0.639 | 0.060 |
| 75 | 4(A3a) | Tall | Thailand Tall | THA | 0.990 | 0.010 | 0.030 | 0.043 | 0.026 | 0.894 | 0.006 |
| 76 | 4(A3a) | Tall | Thailand Tall | THA | 0.995 | 0.005 | 0.134 | 0.370 | 0.167 | 0.324 | 0.004 |
| 77 | 4(A3a) | Tall | Thailand Tall | THA | 0.994 | 0.006 | 0.134 | 0.149 | 0.033 | 0.680 | 0.005 |
| 78 | 4(A3a) | Tall | Thailand Tall | THA | 0.831 | 0.169 | 0.017 | 0.029 | 0.300 | 0.548 | 0.106 |
| 79 | 4(A3a) | Tall | Thailand Tall | THA | 0.990 | 0.010 | 0.026 | 0.061 | 0.104 | 0.801 | 0.008 |
| 80 | 4(A3a) | Tall | Ta Tall | VNM | 0.995 | 0.005 | 0.047 | 0.010 | 0.874 | 0.066 | 0.004 |
| 81 | 4(A3a) | Tall | Ta Tall | VNM | 0.995 | 0.005 | 0.178 | 0.203 | 0.060 | 0.555 | 0.004 |
| 82 | 4(A3a) | Tall | Ta Tall | VNM | 0.994 | 0.006 | 0.014 | 0.055 | 0.909 | 0.018 | 0.004 |
| 83 | 4(A3a) | Tall | Ta Tall | VNM | 0.995 | 0.005 | 0.041 | 0.012 | 0.094 | 0.849 | 0.004 |
| 84 | 4(A3a) | Tall | Ta Tall | VNM | 0.995 | 0.005 | 0.137 | 0.489 | 0.237 | 0.133 | 0.004 |
| 85 | 4(A3a) | Tall | Ta Tall | VNM | 0.994 | 0.006 | 0.032 | 0.027 | 0.664 | 0.273 | 0.005 |
| 86 | 4(A3a) | Tall | Ta Tall | VNM | 0.991 | 0.009 | 0.044 | 0.298 | 0.076 | 0.574 | 0.007 |
| 87 | 4(A3a) | Tall | Dau Tall | VNM | 0.995 | 0.005 | 0.014 | 0.010 | 0.075 | 0.897 | 0.004 |
| 88 | 4(A3a) | Tall | Dau Tall | VNM | 0.984 | 0.016 | 0.233 | 0.015 | 0.166 | 0.572 | 0.015 |
| 89 | 4(A3a) | Tall | Dau Tall | VNM | 0.985 | 0.015 | 0.020 | 0.030 | 0.014 | 0.926 | 0.010 |
| 90 | 4(A3a) | Tall | Dau Tall | VNM | 0.995 | 0.005 | 0.024 | 0.103 | 0.062 | 0.808 | 0.004 |
| 91 | 4(A3a) | Tall | Dau Tall | VNM | 0.990 | 0.010 | 0.040 | 0.032 | 0.039 | 0.882 | 0.007 |
| 92 | 4(A3a) | Tall | Dau Tall | VNM | 0.978 | 0.022 | 0.013 | 0.106 | 0.756 | 0.030 | 0.095 |
| 93 | 4(A3a) | Tall | Lubuk Pakam Tall | IDN | 0.830 | 0.170 | 0.085 | 0.020 | 0.040 | 0.764 | 0.091 |
| 94 | 4(A3a) | Tall | Lubuk Pakam Tall | IDN | 0.948 | 0.052 | 0.032 | 0.008 | 0.010 | 0.906 | 0.044 |
| 95 | 4(A3a) | Tall | Lubuk Pakam Tall | IDN | 0.857 | 0.143 | 0.108 | 0.033 | 0.098 | 0.692 | 0.069 |
| 96 | 4(A3a) | Tall | Lubuk Pakam Tall | IDN | 0.879 | 0.121 | 0.150 | 0.130 | 0.110 | 0.514 | 0.096 |
| 97 | 4(A3a) | Tall | Lubuk Pakam Tall | IDN | 0.779 | 0.221 | 0.042 | 0.012 | 0.106 | 0.684 | 0.155 |
| 98 | 4(A3a) | Tall | Thailand Tall | THA | 0.995 | 0.005 | 0.018 | 0.024 | 0.874 | 0.080 | 0.004 |
| 99 | 4(A3a) | Tall | Thailand Tall | THA | 0.985 | 0.015 | 0.057 | 0.045 | 0.238 | 0.648 | 0.012 |
| 100 | 4(A3a) | Tall | Hainan Tall | CHN | 0.991 | 0.009 | 0.039 | 0.027 | 0.011 | 0.916 | 0.006 |
| 101 | 4(A3a) | Tall | Hainan Tall | CHN | 0.859 | 0.141 | 0.053 | 0.302 | 0.057 | 0.506 | 0.082 |
| 102 | 4(A3a) | Tall | Hainan Tall | CHN | 0.987 | 0.013 | 0.060 | 0.417 | 0.069 | 0.444 | 0.011 |
| 103 | 4(A3a) | Tall | Hainan Tall | CHN | 0.992 | 0.008 | 0.067 | 0.080 | 0.012 | 0.835 | 0.006 |
| 104 | 4(A3a) | Tall | Hainan Tall | CHN | 0.995 | 0.005 | 0.075 | 0.447 | 0.040 | 0.434 | 0.004 |
| 105 | 4(A3a) | Tall | Hainan Tall | CHN | 0.994 | 0.006 | 0.103 | 0.038 | 0.056 | 0.799 | 0.004 |
| 106 | 4(A3a) | Tall | Hainan Tall | CHN | 0.992 | 0.008 | 0.017 | 0.031 | 0.112 | 0.834 | 0.006 |
| 107 | 4(A3a) | Tall | Sarawak Tall | MYS | 0.976 | 0.024 | 0.050 | 0.031 | 0.043 | 0.858 | 0.018 |
| 108 | 4(A3a) | Tall | Sarawak Tall | MYS | 0.994 | 0.006 | 0.054 | 0.032 | 0.402 | 0.508 | 0.004 |
| 109 | 4(A3a) | Tall | Sarawak Tall | MYS | 0.445 | 0.555 | 0.070 | 0.158 | 0.150 | 0.137 | 0.485 |
| 110 | 4(A3a) | Tall | Thailand Tall | THA | 0.994 | 0.006 | 0.051 | 0.024 | 0.080 | 0.841 | 0.004 |
| 111 | 4(A3a) | Tall | Thailand Tall | THA | 0.943 | 0.057 | 0.038 | 0.028 | 0.015 | 0.904 | 0.015 |
| 112 | 4(A3a) | Tall | Thailand Tall | THA | 0.995 | 0.005 | 0.075 | 0.263 | 0.486 | 0.172 | 0.004 |
| 113 | 4(A3a) | Tall | Thailand Tall | THA | 0.989 | 0.011 | 0.042 | 0.013 | 0.030 | 0.906 | 0.008 |
| 114 | 4(A3a) | Tall | Thailand Tall | THA | 0.964 | 0.036 | 0.045 | 0.017 | 0.771 | 0.066 | 0.101 |
| 115 | 4(A3a) | Tall | Thailand Tall | THA | 0.994 | 0.006 | 0.072 | 0.015 | 0.551 | 0.356 | 0.005 |
| 116 | 4(A3a) | Tall | Thailand Tall | THA | 0.995 | 0.005 | 0.026 | 0.021 | 0.279 | 0.670 | 0.004 |
| 117 | 4(A3a) | Tall | Thailand Tall | THA | 0.994 | 0.006 | 0.020 | 0.012 | 0.866 | 0.097 | 0.004 |
| 118 | 4(A3a) | Tall | Thailand Tall | THA | 0.993 | 0.007 | 0.014 | 0.052 | 0.025 | 0.903 | 0.005 |
| 119 | 4(A3a) | Tall | Thailand Tall | THA | 0.830 | 0.170 | 0.013 | 0.024 | 0.788 | 0.022 | 0.153 |
| 120 | 4(A3a) | Tall | Thailand Tall | THA | 0.597 | 0.403 | 0.092 | 0.017 | 0.458 | 0.088 | 0.344 |
| 121 | 5(A3b) | Tall | Takome Tall | IDN | 0.995 | 0.005 | 0.014 | 0.026 | 0.942 | 0.014 | 0.004 |
| 122 | 5(A3b) | Tall | Takome Tall | IDN | 0.994 | 0.006 | 0.032 | 0.654 | 0.237 | 0.072 | 0.005 |
| 123 | 5(A3b) | Tall | Takome Tall | IDN | 0.994 | 0.006 | 0.011 | 0.015 | 0.034 | 0.936 | 0.004 |
| 124 | 5(A3b) | Tall | Takome Tall | IDN | 0.995 | 0.005 | 0.012 | 0.014 | 0.017 | 0.954 | 0.004 |
| 125 | 5(A3b) | Tall | Takome Tall | IDN | 0.991 | 0.009 | 0.055 | 0.019 | 0.041 | 0.879 | 0.006 |
| 126 | 5(A3b) | Tall | Tenga Tall | IDN | 0.993 | 0.007 | 0.020 | 0.012 | 0.061 | 0.901 | 0.005 |
| 127 | 5(A3b) | Tall | Tenga Tall | IDN | 0.995 | 0.005 | 0.024 | 0.026 | 0.674 | 0.272 | 0.004 |
| 128 | 5(A3b) | Tall | Tenga Tall | IDN | 0.994 | 0.006 | 0.010 | 0.011 | 0.854 | 0.121 | 0.004 |
| 129 | 5(A3b) | Tall | Tenga Tall | IDN | 0.994 | 0.006 | 0.111 | 0.017 | 0.145 | 0.722 | 0.005 |
| 130 | 5(A3b) | Tall | Tenga Tall | IDN | 0.995 | 0.005 | 0.042 | 0.477 | 0.342 | 0.135 | 0.004 |
| 131 | 5(A3b) | Tall | Palu Tall | IDN | 0.995 | 0.005 | 0.111 | 0.019 | 0.038 | 0.827 | 0.004 |
| 132 | 5(A3b) | Tall | Palu Tall | IDN | 0.994 | 0.006 | 0.075 | 0.052 | 0.102 | 0.767 | 0.004 |
| 133 | 5(A3b) | Tall | Palu Tall | IDN | 0.994 | 0.006 | 0.034 | 0.119 | 0.042 | 0.801 | 0.004 |
| 134 | 5(A3b) | Tall | Palu Tall | IDN | 0.991 | 0.009 | 0.016 | 0.077 | 0.136 | 0.764 | 0.006 |
| 135 | 5(A3b) | Tall | Palu Tall | IDN | 0.995 | 0.005 | 0.028 | 0.053 | 0.040 | 0.875 | 0.004 |
| 136 | 5(A3b) | Tall | Sawarna Tall | IDN | 0.801 | 0.199 | 0.176 | 0.019 | 0.023 | 0.645 | 0.137 |
| 137 | 5(A3b) | Tall | Sawarna Tall | IDN | 0.766 | 0.234 | 0.284 | 0.075 | 0.033 | 0.409 | 0.200 |
| 138 | 5(A3b) | Tall | Sawarna Tall | IDN | 0.983 | 0.017 | 0.098 | 0.088 | 0.047 | 0.757 | 0.010 |
| 139 | 5(A3b) | Tall | Sawarna Tall | IDN | 0.994 | 0.006 | 0.064 | 0.041 | 0.261 | 0.629 | 0.005 |
| 140 | 5(A3b) | Tall | Sawarna Tall | IDN | 0.983 | 0.017 | 0.340 | 0.025 | 0.183 | 0.426 | 0.025 |
| 141 | 5(A3b) | Tall | Kopyor | IDN | 0.994 | 0.006 | 0.124 | 0.014 | 0.476 | 0.381 | 0.005 |
| 142 | 5(A3b) | Tall | Kopyor | IDN | 0.995 | 0.005 | 0.010 | 0.011 | 0.962 | 0.013 | 0.004 |
| 143 | 5(A3b) | Tall | Kopyor | IDN | 0.995 | 0.005 | 0.011 | 0.011 | 0.960 | 0.014 | 0.004 |
| 144 | 5(A3b) | Tall | Kopyor | IDN | 0.995 | 0.005 | 0.211 | 0.097 | 0.278 | 0.410 | 0.004 |
| 145 | 5(A3b) | Tall | Kopyor | IDN | 0.995 | 0.005 | 0.011 | 0.011 | 0.962 | 0.012 | 0.004 |
| 146 | 6(A3c) | Tall | Tagnanan Tall | PHL | 0.995 | 0.005 | 0.018 | 0.205 | 0.742 | 0.032 | 0.004 |
| 147 | 6(A3c) | Tall | Tagnanan Tall | PHL | 0.995 | 0.005 | 0.050 | 0.699 | 0.128 | 0.119 | 0.004 |
| 148 | 6(A3c) | Tall | Baybay Tall | PHL | 0.995 | 0.005 | 0.012 | 0.033 | 0.090 | 0.861 | 0.004 |
| 149 | 6(A3c) | Tall | Baybay Tall | PHL | 0.995 | 0.005 | 0.354 | 0.288 | 0.070 | 0.283 | 0.004 |
| 150 | 6(A3c) | Tall | Baybay Tall | PHL | 0.992 | 0.008 | 0.300 | 0.292 | 0.025 | 0.377 | 0.007 |
| 151 | 6(A3c) | Tall | Baybay Tall | PHL | 0.995 | 0.005 | 0.019 | 0.366 | 0.025 | 0.587 | 0.004 |
| 152 | 6(A3c) | Tall | Baybay Tall | PHL | 0.995 | 0.005 | 0.479 | 0.374 | 0.016 | 0.126 | 0.004 |
| 153 | 6(A3c) | Tall | Tagnanan Tall | PHL | 0.995 | 0.005 | 0.024 | 0.083 | 0.866 | 0.022 | 0.004 |
| 154 | 6(A3c) | Tall | Tagnanan Tall | PHL | 0.995 | 0.005 | 0.051 | 0.531 | 0.360 | 0.054 | 0.004 |
| 155 | 6(A3c) | Tall | Tagnanan Tall | PHL | 0.995 | 0.005 | 0.033 | 0.471 | 0.465 | 0.027 | 0.004 |
| 156 | 6(A3c) | Tall | Tagnanan Tall | PHL | 0.995 | 0.005 | 0.021 | 0.012 | 0.035 | 0.928 | 0.004 |
| 157 | 6(A3c) | Tall | San Ramon Tall | PHL | 0.994 | 0.006 | 0.041 | 0.022 | 0.473 | 0.460 | 0.005 |
| 158 | 6(A3c) | Tall | Baybay Tall | PHL | 0.995 | 0.005 | 0.013 | 0.135 | 0.105 | 0.742 | 0.004 |
| 159 | 6(A3c) | Tall | Mexican Pacific Tall Michoacan | MEX | 0.990 | 0.010 | 0.100 | 0.113 | 0.107 | 0.672 | 0.008 |
| 160 | 6(A3c) | Tall | Mexican Pacific Tall Michoacan | MEX | 0.991 | 0.009 | 0.033 | 0.054 | 0.857 | 0.042 | 0.015 |
| 161 | 6(A3c) | Tall | Mexican Pacific Tall Michoacan | MEX | 0.990 | 0.010 | 0.025 | 0.312 | 0.079 | 0.577 | 0.008 |
| 162 | 6(A3c) | Tall | Mexican Pacific Tall Michoacan | MEX | 0.994 | 0.006 | 0.014 | 0.296 | 0.498 | 0.187 | 0.005 |
| 163 | 6(A3c) | Tall | Mexican Pacific Tall Michoacan | MEX | 0.994 | 0.006 | 0.008 | 0.056 | 0.920 | 0.012 | 0.004 |
| 164 | 6(A3c) | Tall | Mexican Pacific Tall Colima | MEX | 0.981 | 0.019 | 0.211 | 0.146 | 0.024 | 0.607 | 0.013 |
| 165 | 6(A3c) | Tall | Mexican Pacific Tall Colima | MEX | 0.983 | 0.017 | 0.135 | 0.168 | 0.060 | 0.626 | 0.011 |
| 166 | 6(A3c) | Tall | Mexican Pacific Tall Colima | MEX | 0.973 | 0.027 | 0.342 | 0.011 | 0.435 | 0.191 | 0.021 |
| 167 | 6(A3c) | Tall | Mexican Pacific Tall Colima | MEX | 0.951 | 0.049 | 0.827 | 0.013 | 0.025 | 0.096 | 0.039 |
| 168 | 6(A3c) | Tall | Mexican Pacific Tall Colima | MEX | 0.994 | 0.006 | 0.024 | 0.018 | 0.909 | 0.044 | 0.005 |
| 169 | 6(A3c) | Tall | Baybay Tall | PHL | 0.993 | 0.007 | 0.016 | 0.866 | 0.072 | 0.031 | 0.015 |
| 170 | 6(A3c) | Tall | Baybay Tall | PHL | 0.995 | 0.005 | 0.037 | 0.252 | 0.014 | 0.693 | 0.004 |
| 171 | 6(A3c) | Tall | Ballesteros Tall Tarraq | PHL | 0.995 | 0.005 | 0.381 | 0.387 | 0.036 | 0.191 | 0.004 |
| 172 | 6(A3c) | Tall | Ballesteros Tall Tarraq | PHL | 0.995 | 0.005 | 0.252 | 0.034 | 0.056 | 0.654 | 0.004 |
| 173 | 6(A3c) | Tall | Ballesteros Tall Tarraq | PHL | 0.995 | 0.005 | 0.056 | 0.270 | 0.038 | 0.632 | 0.004 |
| 174 | 6(A3c) | Tall | Ballesteros Tall Tarraq | PHL | 0.993 | 0.007 | 0.084 | 0.042 | 0.766 | 0.104 | 0.004 |
| 175 | 6(A3c) | Tall | Ballesteros Tall Tarraq | PHL | 0.994 | 0.006 | 0.433 | 0.073 | 0.118 | 0.372 | 0.005 |
| 176 | 6(A3c) | Tall | Ballesteros Tall Tarraq | PHL | 0.992 | 0.008 | 0.107 | 0.075 | 0.071 | 0.741 | 0.006 |
| 177 | 6(A3c) | Tall | Ballesteros Tall Tarraq | PHL | 0.994 | 0.006 | 0.037 | 0.043 | 0.420 | 0.495 | 0.004 |
| 178 | 6(A3c) | Tall | Pandan Tall | PHL | 0.994 | 0.006 | 0.130 | 0.633 | 0.017 | 0.215 | 0.005 |
| 179 | 6(A3c) | Tall | Pandan Tall | PHL | 0.993 | 0.007 | 0.224 | 0.310 | 0.032 | 0.430 | 0.005 |
| 180 | 6(A3c) | Tall | Pandan Tall | PHL | 0.936 | 0.064 | 0.064 | 0.334 | 0.024 | 0.546 | 0.032 |
| 181 | 6(A3c) | Tall | Pandan Tall | PHL | 0.994 | 0.006 | 0.087 | 0.794 | 0.025 | 0.090 | 0.005 |
| 182 | 6(A3c) | Tall | Pandan Tall | PHL | 0.949 | 0.051 | 0.017 | 0.098 | 0.781 | 0.057 | 0.046 |
| 183 | 6(A3c) | Tall | Pandan Tall | PHL | 0.995 | 0.005 | 0.178 | 0.321 | 0.181 | 0.317 | 0.004 |
| 184 | 6(A3c) | Tall | San Ramon Tall | PHL | 0.995 | 0.005 | 0.073 | 0.368 | 0.082 | 0.472 | 0.004 |
| 185 | 6(A3c) | Tall | San Ramon Tall | PHL | 0.995 | 0.005 | 0.184 | 0.518 | 0.031 | 0.264 | 0.004 |
| 186 | 6(A3c) | Tall | San Ramon Tall | PHL | 0.995 | 0.005 | 0.031 | 0.305 | 0.043 | 0.617 | 0.004 |
| 187 | 6(A3c) | Tall | San Ramon Tall | PHL | 0.993 | 0.007 | 0.093 | 0.462 | 0.202 | 0.236 | 0.006 |
| 188 | 6(A3c) | Tall | San Ramon Tall | PHL | 0.808 | 0.192 | 0.010 | 0.763 | 0.019 | 0.013 | 0.194 |
| 189 | 6(A3c) | Tall | Mexican Pacific Tall Michoacan | MEX | 0.989 | 0.011 | 0.028 | 0.143 | 0.790 | 0.026 | 0.012 |
| 190 | 6(A3c) | Tall | Mexican Pacific Tall Michoacan | MEX | 0.990 | 0.010 | 0.027 | 0.314 | 0.078 | 0.574 | 0.008 |
| 191 | 6(A3c) | Tall | Mexican Pacific Tall Michoacan | MEX | 0.994 | 0.006 | 0.014 | 0.307 | 0.492 | 0.182 | 0.005 |
| 192 | 6(A3c) | Tall | Mexican Pacific Tall Michoacan | MEX | 0.994 | 0.006 | 0.440 | 0.038 | 0.280 | 0.238 | 0.005 |
| 193 | 6(A3c) | Tall | Mexican Pacific Tall Michoacan | MEX | 0.994 | 0.006 | 0.018 | 0.007 | 0.840 | 0.131 | 0.004 |
| 194 | 6(A3c) | Tall | Mexican Pacific Tall Michoacan | MEX | 0.993 | 0.007 | 0.054 | 0.034 | 0.071 | 0.835 | 0.005 |
| 195 | 6(A3c) | Tall | Mexican Pacific Tall Michoacan | MEX | 0.994 | 0.006 | 0.454 | 0.011 | 0.146 | 0.385 | 0.005 |
| 196 | 6(A3c) | Tall | Mexican Pacific Tall Michoacan | MEX | 0.995 | 0.005 | 0.105 | 0.025 | 0.113 | 0.752 | 0.004 |
| 197 | 6(A3c) | Tall | Mexican Pacific Tall Michoacan | MEX | 0.994 | 0.006 | 0.601 | 0.017 | 0.139 | 0.240 | 0.004 |
| 198 | 6(A3c) | Tall | Mexican Pacific Tall Guerrero | MEX | 0.991 | 0.009 | 0.270 | 0.554 | 0.018 | 0.147 | 0.011 |
| 199 | 6(A3c) | Tall | Mexican Pacific Tall Guerrero | MEX | 0.994 | 0.006 | 0.057 | 0.044 | 0.553 | 0.340 | 0.007 |
| 200 | 6(A3c) | Tall | Mexican Pacific Tall Guerrero | MEX | 0.993 | 0.007 | 0.018 | 0.185 | 0.772 | 0.021 | 0.004 |
| 201 | 6(A3c) | Tall | Mexican Pacific Tall Guerrero | MEX | 0.995 | 0.005 | 0.015 | 0.330 | 0.608 | 0.044 | 0.004 |
| 202 | 6(A3c) | Tall | Mexican Pacific Tall Guerrero | MEX | 0.994 | 0.006 | 0.030 | 0.006 | 0.100 | 0.859 | 0.005 |
| 203 | 6(A3c) | Tall | Mexican Pacific Tall Guerrero | MEX | 0.995 | 0.005 | 0.093 | 0.012 | 0.456 | 0.435 | 0.004 |
| 204 | 6(A3c) | Tall | Mexican Pacific Tall Colima | MEX | 0.916 | 0.084 | 0.635 | 0.018 | 0.133 | 0.128 | 0.086 |
| 205 | 6(A3c) | Tall | Mexican Pacific Tall Colima | MEX | 0.992 | 0.008 | 0.469 | 0.024 | 0.024 | 0.476 | 0.007 |
| 206 | 6(A3c) | Tall | Mexican Pacific Tall Colima | MEX | 0.995 | 0.005 | 0.942 | 0.022 | 0.014 | 0.018 | 0.004 |
| 207 | 6(A3c) | Tall | Mexican Pacific Tall Colima | MEX | 0.993 | 0.007 | 0.703 | 0.022 | 0.029 | 0.240 | 0.005 |
| 208 | 6(A3c) | Tall | Mexican Pacific Tall Colima | MEX | 0.978 | 0.022 | 0.366 | 0.011 | 0.039 | 0.570 | 0.013 |
| 209 | 6(A3c) | Tall | Mexican Pacific Tall Colima | MEX | 0.995 | 0.005 | 0.241 | 0.014 | 0.043 | 0.699 | 0.004 |
| 210 | 6(A3c) | Tall | Mexican Pacific Tall Colima | MEX | 0.994 | 0.006 | 0.032 | 0.076 | 0.073 | 0.815 | 0.004 |
| 211 | 6(A3c) | Tall | Mexican Pacific Tall Colima | MEX | 0.995 | 0.005 | 0.121 | 0.286 | 0.296 | 0.293 | 0.004 |
| 212 | 6(A3c) | Tall | Mexican Pacific Tall Colima | MEX | 0.973 | 0.027 | 0.147 | 0.076 | 0.089 | 0.667 | 0.021 |
| 213 | 6(A3c) | Tall | Mexican Pacific Tall Guerrero | MEX | 0.994 | 0.006 | 0.055 | 0.045 | 0.550 | 0.343 | 0.007 |
| 214 | 6(A3c) | Tall | Mexican Pacific Tall Guerrero | MEX | 0.995 | 0.005 | 0.121 | 0.352 | 0.103 | 0.420 | 0.004 |
| 215 | 6(A3c) | Tall | Mexican Pacific Tall Guerrero | MEX | 0.991 | 0.009 | 0.268 | 0.555 | 0.017 | 0.149 | 0.011 |
| 216 | 6(A3c) | Tall | Mexican Pacific Tall Guerrero | MEX | 0.990 | 0.010 | 0.107 | 0.645 | 0.103 | 0.136 | 0.010 |
| 217 | 6(A3c) | Tall | Mexican Pacific Tall Guerrero | MEX | 0.993 | 0.007 | 0.018 | 0.180 | 0.774 | 0.022 | 0.004 |
| 218 | 6(A3c) | Tall | Mexican Pacific Tall Nuxco | MEX | 0.994 | 0.006 | 0.109 | 0.014 | 0.546 | 0.326 | 0.005 |
| 219 | 6(A3c) | Tall | Mexican Pacific Tall Nuxco | MEX | 0.994 | 0.006 | 0.118 | 0.065 | 0.082 | 0.730 | 0.005 |
| 220 | 6(A3c) | Tall | Mexican Pacific Tall Nuxco | MEX | 0.994 | 0.006 | 0.074 | 0.419 | 0.430 | 0.073 | 0.004 |
| 221 | 6(A3c) | Tall | Mexican Pacific Tall Nuxco | MEX | 0.995 | 0.005 | 0.038 | 0.019 | 0.065 | 0.875 | 0.004 |
| 222 | 6(A3c) | Tall | Tagnanan Tall | PHL | 0.995 | 0.005 | 0.129 | 0.648 | 0.022 | 0.197 | 0.004 |
| 223 | 6(A3c) | Tall | Tagnanan Tall | PHL | 0.995 | 0.005 | 0.021 | 0.196 | 0.748 | 0.032 | 0.004 |
| 224 | 6(A3c) | Tall | Tagnanan Tall | PHL | 0.994 | 0.006 | 0.018 | 0.036 | 0.928 | 0.014 | 0.004 |
| 225 | 6(A3c) | Tall | Tagnanan Tall | PHL | 0.995 | 0.005 | 0.017 | 0.381 | 0.537 | 0.061 | 0.004 |
| 226 | 6(A3c) | Tall | Tagnanan Tall | PHL | 0.995 | 0.005 | 0.021 | 0.061 | 0.290 | 0.624 | 0.004 |
| 227 | 6(A3c) | Tall | Tagnanan Tall | PHL | 0.995 | 0.005 | 0.019 | 0.091 | 0.813 | 0.074 | 0.004 |
| 228 | 6(A3c) | Tall | Tagnanan Tall | PHL | 0.995 | 0.005 | 0.031 | 0.623 | 0.198 | 0.145 | 0.004 |
| 229 | 6(A3c) | Tall | Tagnanan Tall | PHL | 0.995 | 0.005 | 0.015 | 0.114 | 0.846 | 0.021 | 0.004 |
| 230 | 6(A3c) | Tall | Macapuno Tall | PHL | 0.994 | 0.006 | 0.011 | 0.061 | 0.906 | 0.018 | 0.005 |
| 231 | 6(A3c) | Tall | Macapuno Tall | PHL | 0.995 | 0.005 | 0.051 | 0.095 | 0.774 | 0.076 | 0.004 |
| 232 | 6(A3c) | Tall | Macapuno Tall | PHL | 0.995 | 0.005 | 0.052 | 0.044 | 0.843 | 0.059 | 0.004 |
| 233 | 6(A3c) | Tall | Macapuno Tall | PHL | 0.989 | 0.011 | 0.075 | 0.442 | 0.243 | 0.228 | 0.012 |
| 234 | 6(A3c) | Tall | Macapuno Tall | PHL | 0.995 | 0.005 | 0.047 | 0.048 | 0.832 | 0.069 | 0.004 |
| 235 | 7(A4a) | Tall | Kar Kar Tall | PNG | 0.986 | 0.014 | 0.031 | 0.016 | 0.023 | 0.921 | 0.009 |
| 236 | 7(A4a) | Tall | Kar Kar Tall | PNG | 0.984 | 0.016 | 0.139 | 0.006 | 0.027 | 0.819 | 0.009 |
| 237 | 7(A4a) | Tall | Kar Kar Tall | PNG | 0.992 | 0.008 | 0.239 | 0.329 | 0.026 | 0.398 | 0.007 |
| 238 | 7(A4a) | Tall | Kar Kar Tall | PNG | 0.983 | 0.017 | 0.748 | 0.008 | 0.013 | 0.218 | 0.014 |
| 239 | 7(A4a) | Tall | Kar Kar Tall | PNG | 0.994 | 0.006 | 0.181 | 0.027 | 0.019 | 0.768 | 0.004 |
| 240 | 7(A4a) | Tall | East Sepik Tall Yangoru | PNG | 0.991 | 0.009 | 0.115 | 0.031 | 0.164 | 0.684 | 0.007 |
| 241 | 7(A4a) | Tall | East Sepik Tall Yangoru | PNG | 0.991 | 0.009 | 0.056 | 0.011 | 0.021 | 0.907 | 0.006 |
| 242 | 7(A4a) | Tall | East Sepik Tall Yangoru | PNG | 0.994 | 0.006 | 0.592 | 0.108 | 0.028 | 0.266 | 0.005 |
| 243 | 7(A4a) | Tall | East Sepik Tall Vokio | PNG | 0.994 | 0.006 | 0.029 | 0.007 | 0.011 | 0.950 | 0.004 |
| 244 | 7(A4a) | Tall | Madang Red Tall | PNG | 0.992 | 0.008 | 0.074 | 0.007 | 0.017 | 0.896 | 0.005 |
| 245 | 7(A4a) | Tall | Madang Red Tall | PNG | 0.993 | 0.007 | 0.143 | 0.309 | 0.049 | 0.494 | 0.005 |
| 246 | 7(A4a) | Tall | Madang Red Tall | PNG | 0.993 | 0.007 | 0.082 | 0.006 | 0.101 | 0.806 | 0.005 |
| 247 | 7(A4a) | Tall | Madang Red Tall | PNG | 0.995 | 0.005 | 0.201 | 0.020 | 0.012 | 0.763 | 0.004 |
| 248 | 7(A4a) | Tall | Madang Yellow tall | PNG | 0.991 | 0.009 | 0.014 | 0.065 | 0.629 | 0.283 | 0.008 |
| 249 | 7(A4a) | Tall | Madang Yellow tall | PNG | 0.985 | 0.015 | 0.268 | 0.109 | 0.231 | 0.375 | 0.016 |
| 250 | 7(A4a) | Tall | Madang Yellow tall | PNG | 0.974 | 0.026 | 0.018 | 0.012 | 0.037 | 0.919 | 0.014 |
| 251 | 7(A4a) | Tall | Madang Yellow tall | PNG | 0.995 | 0.005 | 0.015 | 0.063 | 0.103 | 0.816 | 0.004 |
| 252 | 7(A4a) | Tall | Karkar Tall Guanaga | PNG | 0.993 | 0.007 | 0.240 | 0.012 | 0.015 | 0.728 | 0.005 |
| 253 | 7(A4a) | Tall | Karkar Tall Guanaga | PNG | 0.995 | 0.005 | 0.242 | 0.010 | 0.569 | 0.175 | 0.004 |
| 254 | 7(A4a) | Tall | Karkar Tall Guanaga | PNG | 0.995 | 0.005 | 0.459 | 0.019 | 0.020 | 0.498 | 0.004 |
| 255 | 7(A4a) | Tall | Karkar Tall Guanaga | PNG | 0.994 | 0.006 | 0.507 | 0.014 | 0.021 | 0.454 | 0.004 |
| 256 | 7(A4a) | Tall | Karkar Tall Guanaga | PNG | 0.991 | 0.009 | 0.261 | 0.013 | 0.162 | 0.557 | 0.007 |
| 257 | 7(A4a) | Tall | Karkar Tall Kinim | PNG | 0.993 | 0.007 | 0.022 | 0.033 | 0.879 | 0.061 | 0.005 |
| 258 | 7(A4a) | Tall | Karkar Tall Kinim | PNG | 0.991 | 0.009 | 0.050 | 0.044 | 0.098 | 0.802 | 0.006 |
| 259 | 7(A4a) | Tall | Karkar Tall Kinim | PNG | 0.993 | 0.007 | 0.290 | 0.024 | 0.187 | 0.493 | 0.005 |
| 260 | 7(A4a) | Tall | Karkar Tall Kinim | PNG | 0.991 | 0.009 | 0.121 | 0.010 | 0.113 | 0.750 | 0.006 |
| 261 | 7(A4a) | Tall | Karkar Tall Ulatava | PNG | 0.991 | 0.009 | 0.903 | 0.008 | 0.015 | 0.068 | 0.006 |
| 262 | 7(A4a) | Tall | Karkar Tall Ulatava | PNG | 0.990 | 0.010 | 0.013 | 0.012 | 0.011 | 0.958 | 0.006 |
| 263 | 7(A4a) | Tall | Karkar Tall Ulatava | PNG | 0.992 | 0.008 | 0.779 | 0.010 | 0.014 | 0.192 | 0.006 |
| 264 | 7(A4a) | Tall | Karkar Tall Ulatava | PNG | 0.992 | 0.008 | 0.096 | 0.007 | 0.015 | 0.876 | 0.005 |
| 265 | 7(A4a) | Tall | East Sepik Tall Vokio | PNG | 0.994 | 0.006 | 0.043 | 0.233 | 0.021 | 0.698 | 0.004 |
| 266 | 7(A4a) | Tall | East Sepik Tall Vokio | PNG | 0.923 | 0.077 | 0.145 | 0.009 | 0.162 | 0.626 | 0.057 |
| 267 | 7(A4a) | Tall | East Sepik Tall Vokio | PNG | 0.994 | 0.006 | 0.281 | 0.484 | 0.037 | 0.193 | 0.004 |
| 268 | 7(A4a) | Tall | East Sepik Tall Vokio | PNG | 0.993 | 0.007 | 0.029 | 0.017 | 0.068 | 0.881 | 0.005 |
| 269 | 7(A4a) | Tall | Karkar Tall Kinim | PNG | 0.994 | 0.006 | 0.092 | 0.026 | 0.074 | 0.805 | 0.004 |
| 270 | 7(A4a) | Tall | Karkar Tall Kinim | PNG | 0.990 | 0.010 | 0.023 | 0.021 | 0.225 | 0.724 | 0.007 |
| 271 | 7(A4a) | Tall | Karkar Tall Kinim | PNG | 0.993 | 0.007 | 0.150 | 0.030 | 0.568 | 0.243 | 0.009 |
| 272 | 7(A4a) | Tall | Karkar Tall Kinim | PNG | 0.994 | 0.006 | 0.040 | 0.100 | 0.015 | 0.840 | 0.005 |
| 273 | 8(A4b) | Tall | Baibara Tall | PNG | 0.992 | 0.008 | 0.752 | 0.043 | 0.119 | 0.081 | 0.006 |
| 274 | 8(A4b) | Tall | Baibara Tall | PNG | 0.995 | 0.005 | 0.135 | 0.024 | 0.014 | 0.824 | 0.004 |
| 275 | 8(A4b) | Tall | Baibara Tall | PNG | 0.995 | 0.005 | 0.342 | 0.046 | 0.011 | 0.596 | 0.004 |
| 276 | 8(A4b) | Tall | Baibara Tall | PNG | 0.995 | 0.005 | 0.292 | 0.038 | 0.547 | 0.119 | 0.004 |
| 277 | 8(A4b) | Tall | Baibara Tall | PNG | 0.994 | 0.006 | 0.084 | 0.016 | 0.009 | 0.887 | 0.004 |
| 278 | 8(A4b) | Tall | Hisihu Tall | PNG | 0.993 | 0.007 | 0.044 | 0.007 | 0.008 | 0.937 | 0.005 |
| 279 | 8(A4b) | Tall | Hisihu Tall | PNG | 0.992 | 0.008 | 0.043 | 0.494 | 0.016 | 0.441 | 0.005 |
| 280 | 8(A4b) | Tall | Hisihu Tall | PNG | 0.984 | 0.016 | 0.185 | 0.021 | 0.021 | 0.763 | 0.010 |
| 281 | 8(A4b) | Tall | Hisihu Tall | PNG | 0.993 | 0.007 | 0.045 | 0.018 | 0.010 | 0.922 | 0.004 |
| 282 | 8(A4b) | Tall | Hisihu Tall | PNG | 0.994 | 0.006 | 0.480 | 0.032 | 0.020 | 0.464 | 0.004 |
| 283 | 8(A4b) | Tall | Poligolo Tall | PNG | 0.976 | 0.024 | 0.134 | 0.012 | 0.013 | 0.810 | 0.031 |
| 284 | 8(A4b) | Tall | Poligolo Tall | PNG | 0.993 | 0.007 | 0.036 | 0.137 | 0.015 | 0.807 | 0.005 |
| 285 | 8(A4b) | Tall | Poligolo Tall | PNG | 0.994 | 0.006 | 0.041 | 0.119 | 0.011 | 0.825 | 0.004 |
| 286 | 8(A4b) | Tall | Vailala Tall Keakea | PNG | 0.981 | 0.019 | 0.057 | 0.848 | 0.017 | 0.038 | 0.039 |
| 287 | 8(A4b) | Tall | Vailala Tall Keakea | PNG | 0.990 | 0.010 | 0.086 | 0.124 | 0.019 | 0.764 | 0.007 |
| 288 | 8(A4b) | Tall | Vailala Tall Keakea | PNG | 0.972 | 0.028 | 0.026 | 0.070 | 0.011 | 0.877 | 0.016 |
| 289 | 8(A4b) | Tall | Vailala Tall Keakea | PNG | 0.989 | 0.011 | 0.054 | 0.012 | 0.012 | 0.915 | 0.007 |
| 290 | 8(A4b) | Tall | Vailala Tall Keakea | PNG | 0.996 | 0.004 | 0.037 | 0.009 | 0.013 | 0.937 | 0.004 |
| 291 | 8(A4b) | Tall | Vailala Tall | PNG | 0.994 | 0.006 | 0.059 | 0.141 | 0.011 | 0.784 | 0.004 |
| 292 | 8(A4b) | Tall | Vailala Tall | PNG | 0.987 | 0.013 | 0.059 | 0.025 | 0.012 | 0.896 | 0.009 |
| 293 | 8(A4b) | Tall | Vailala Tall | PNG | 0.993 | 0.007 | 0.019 | 0.017 | 0.014 | 0.944 | 0.005 |
| 294 | 8(A4b) | Tall | Vailala Tall | PNG | 0.991 | 0.009 | 0.217 | 0.020 | 0.010 | 0.746 | 0.006 |
| 295 | 8(A4b) | Tall | Vailala Tall | PNG | 0.993 | 0.007 | 0.084 | 0.021 | 0.010 | 0.879 | 0.005 |
| 296 | 8(A4b) | Tall | Kiwai Tall Boze | PNG | 0.994 | 0.006 | 0.013 | 0.085 | 0.086 | 0.811 | 0.005 |
| 297 | 8(A4b) | Tall | Kiwai Tall Boze | PNG | 0.981 | 0.019 | 0.145 | 0.027 | 0.015 | 0.801 | 0.012 |
| 298 | 8(A4b) | Tall | Kiwai Tall Boze | PNG | 0.990 | 0.010 | 0.143 | 0.086 | 0.088 | 0.677 | 0.008 |
| 299 | 8(A4b) | Tall | Kiwai Tall Boze | PNG | 0.994 | 0.006 | 0.271 | 0.007 | 0.011 | 0.706 | 0.004 |
| 300 | 8(A4b) | Tall | Milne Bay Tall Siagara | PNG | 0.994 | 0.006 | 0.625 | 0.095 | 0.063 | 0.214 | 0.005 |
| 301 | 8(A4b) | Tall | Oro Tall Ajoa | PNG | 0.995 | 0.005 | 0.465 | 0.018 | 0.019 | 0.495 | 0.004 |
| 302 | 8(A4b) | Tall | Oro Tall Ajoa | PNG | 0.987 | 0.013 | 0.053 | 0.029 | 0.040 | 0.870 | 0.008 |
| 303 | 8(A4b) | Tall | Oro Tall Ajoa | PNG | 0.988 | 0.012 | 0.187 | 0.354 | 0.040 | 0.409 | 0.010 |
| 304 | 8(A4b) | Tall | Oro Tall Ajoa | PNG | 0.990 | 0.010 | 0.586 | 0.012 | 0.014 | 0.382 | 0.006 |
| 305 | 8(A4b) | Tall | Milne Bay Tall Siagara | PNG | 0.995 | 0.005 | 0.682 | 0.058 | 0.020 | 0.236 | 0.004 |
| 306 | 8(A4b) | Tall | Milne Bay Tall Siagara | PNG | 0.976 | 0.024 | 0.072 | 0.020 | 0.007 | 0.887 | 0.013 |
| 307 | 9(A4c) | Tall | Gazelle Peninsula Tall | PNG | 0.988 | 0.012 | 0.279 | 0.005 | 0.012 | 0.696 | 0.008 |
| 308 | 9(A4c) | Tall | Gazelle Peninsula Tall | PNG | 0.975 | 0.025 | 0.034 | 0.016 | 0.011 | 0.928 | 0.011 |
| 309 | 9(A4c) | Tall | Gazelle Peninsula Tall | PNG | 0.991 | 0.009 | 0.022 | 0.059 | 0.136 | 0.777 | 0.006 |
| 310 | 9(A4c) | Tall | Gazelle Peninsula Tall | PNG | 0.991 | 0.009 | 0.052 | 0.009 | 0.012 | 0.921 | 0.006 |
| 311 | 9(A4c) | Tall | Gazelle Peninsula Tall | PNG | 0.987 | 0.013 | 0.073 | 0.008 | 0.054 | 0.857 | 0.007 |
| 312 | 9(A4c) | Tall | Manus Tall | PNG | 0.992 | 0.008 | 0.538 | 0.009 | 0.229 | 0.215 | 0.009 |
| 313 | 9(A4c) | Tall | Manus Tall | PNG | 0.995 | 0.005 | 0.816 | 0.008 | 0.030 | 0.143 | 0.004 |
| 314 | 9(A4c) | Tall | Manus Tall | PNG | 0.994 | 0.006 | 0.035 | 0.006 | 0.154 | 0.800 | 0.004 |
| 315 | 9(A4c) | Tall | Manus Tall | PNG | 0.994 | 0.006 | 0.022 | 0.014 | 0.045 | 0.915 | 0.004 |
| 316 | 9(A4c) | Tall | Manus Tall | PNG | 0.994 | 0.006 | 0.113 | 0.009 | 0.095 | 0.778 | 0.005 |
| 317 | 9(A4c) | Tall | Gazelle Peninsula Tall New Massawa | PNG | 0.990 | 0.010 | 0.348 | 0.022 | 0.012 | 0.611 | 0.007 |
| 318 | 9(A4c) | Tall | Gazelle Peninsula Tall New Massawa | PNG | 0.994 | 0.006 | 0.037 | 0.013 | 0.010 | 0.936 | 0.005 |
| 319 | 9(A4c) | Tall | Gazelle Peninsula Tall New Massawa | PNG | 0.975 | 0.025 | 0.332 | 0.037 | 0.044 | 0.570 | 0.017 |
| 320 | 9(A4c) | Tall | Gazelle Peninsula Tall New Massawa | PNG | 0.993 | 0.007 | 0.018 | 0.060 | 0.041 | 0.876 | 0.005 |
| 321 | 9(A4c) | Tall | Gazelle Peninsula Tall New Massawa | PNG | 0.991 | 0.009 | 0.061 | 0.078 | 0.024 | 0.831 | 0.006 |
| 322 | 9(A4c) | Tall | Gazelle Peninsula Tall Pellavarua | PNG | 0.992 | 0.008 | 0.546 | 0.007 | 0.010 | 0.430 | 0.006 |
| 323 | 9(A4c) | Tall | Gazelle Peninsula Tall Pellavarua | PNG | 0.961 | 0.039 | 0.065 | 0.019 | 0.018 | 0.888 | 0.011 |
| 324 | 9(A4c) | Tall | Gazelle Peninsula Tall Pellavarua | PNG | 0.992 | 0.008 | 0.022 | 0.016 | 0.022 | 0.934 | 0.006 |
| 325 | 9(A4c) | Tall | West New Britain Tall Gaungo | PNG | 0.987 | 0.013 | 0.051 | 0.100 | 0.030 | 0.811 | 0.008 |
| 326 | 9(A4c) | Tall | West New Britain Tall Gaungo | PNG | 0.986 | 0.014 | 0.043 | 0.011 | 0.008 | 0.931 | 0.008 |
| 327 | 9(A4c) | Tall | West New Britain Tall Gaungo | PNG | 0.995 | 0.005 | 0.034 | 0.170 | 0.017 | 0.775 | 0.004 |
| 328 | 9(A4c) | Tall | West New Britain Tall Gaungo | PNG | 0.990 | 0.010 | 0.434 | 0.367 | 0.021 | 0.171 | 0.007 |
| 329 | 9(A4c) | Tall | West New Britain Tall Gaungo | PNG | 0.992 | 0.008 | 0.088 | 0.171 | 0.021 | 0.714 | 0.006 |
| 330 | 9(A4c) | Tall | West New Britain Tall Naviro | PNG | 0.995 | 0.005 | 0.019 | 0.016 | 0.009 | 0.952 | 0.004 |
| 331 | 9(A4c) | Tall | West New Britain Tall Naviro | PNG | 0.985 | 0.015 | 0.024 | 0.057 | 0.018 | 0.893 | 0.009 |
| 332 | 9(A4c) | Tall | Gazelle Peninsula Tall | PNG | 0.990 | 0.010 | 0.724 | 0.012 | 0.015 | 0.243 | 0.007 |
| 333 | 9(A4c) | Tall | Gazelle Peninsula Tall | PNG | 0.993 | 0.007 | 0.040 | 0.010 | 0.032 | 0.914 | 0.005 |
| 334 | 9(A4c) | Tall | Gazelle Peninsula Tall | PNG | 0.993 | 0.007 | 0.070 | 0.018 | 0.017 | 0.891 | 0.005 |
| 335 | 9(A4c) | Tall | Gazelle Peninsula Tall | PNG | 0.993 | 0.007 | 0.175 | 0.036 | 0.014 | 0.770 | 0.005 |
| 336 | 9(A4c) | Tall | Gazelle Peninsula Tall | PNG | 0.993 | 0.007 | 0.024 | 0.013 | 0.030 | 0.928 | 0.005 |
| 337 | 9(A4c) | Tall | Namatanai Tall Karu Village | PNG | 0.994 | 0.006 | 0.158 | 0.011 | 0.017 | 0.810 | 0.004 |
| 338 | 9(A4c) | Tall | Namatanai Tall Karu Village | PNG | 0.994 | 0.006 | 0.422 | 0.009 | 0.036 | 0.529 | 0.005 |
| 339 | 9(A4c) | Tall | Namatanai Tall Karu Village | PNG | 0.995 | 0.005 | 0.359 | 0.007 | 0.024 | 0.605 | 0.004 |
| 340 | 9(A4c) | Tall | Namatanai Tall Karu Village | PNG | 0.993 | 0.007 | 0.078 | 0.008 | 0.012 | 0.897 | 0.005 |
| 341 | 9(A4c) | Tall | Namatanai Tall Karu Village | PNG | 0.993 | 0.007 | 0.177 | 0.014 | 0.030 | 0.774 | 0.005 |
| 342 | 9(A4c) | Tall | Gazelle Peninsula Tall Pellavarua | PNG | 0.993 | 0.007 | 0.205 | 0.009 | 0.013 | 0.769 | 0.005 |
| 343 | 9(A4c) | Tall | Gazelle Peninsula Tall Pellavarua | PNG | 0.967 | 0.033 | 0.017 | 0.029 | 0.032 | 0.913 | 0.009 |
| 344 | 9(A4c) | Tall | Gazelle Peninsula Tall Natava "many fruited" | PNG | 0.992 | 0.008 | 0.135 | 0.012 | 0.034 | 0.814 | 0.006 |
| 345 | 9(A4c) | Tall | Gazelle Peninsula Tall Natava "many fruited" | PNG | 0.821 | 0.179 | 0.021 | 0.020 | 0.073 | 0.747 | 0.140 |
| 346 | 9(A4c) | Tall | Gazelle Peninsula Tall Natava "many fruited" | PNG | 0.986 | 0.014 | 0.016 | 0.027 | 0.031 | 0.919 | 0.007 |
| 347 | 9(A4c) | Tall | Gazelle Peninsula Tall Natava "many fruited" | PNG | 0.994 | 0.006 | 0.032 | 0.008 | 0.021 | 0.935 | 0.004 |
| 348 | 9(A4c) | Tall | West New Britain Tall Naviro | PNG | 0.995 | 0.005 | 0.020 | 0.017 | 0.044 | 0.916 | 0.004 |
| 349 | 9(A4c) | Tall | West New Britain Tall Naviro | PNG | 0.992 | 0.008 | 0.371 | 0.018 | 0.036 | 0.569 | 0.006 |
| 350 | 9(A4c) | Tall | Namatanai Tall Etalat | PNG | 0.994 | 0.006 | 0.035 | 0.175 | 0.019 | 0.766 | 0.004 |
| 351 | 9(A4c) | Tall | Namatanai Tall Etalat | PNG | 0.993 | 0.007 | 0.183 | 0.028 | 0.642 | 0.143 | 0.004 |
| 352 | 9(A4c) | Tall | Namatanai Tall Etalat | PNG | 0.990 | 0.010 | 0.025 | 0.021 | 0.235 | 0.712 | 0.007 |
| 353 | 9(A4c) | Tall | Namatanai Tall Etalat | PNG | 0.945 | 0.055 | 0.848 | 0.021 | 0.044 | 0.067 | 0.020 |
| 354 | 9(A4c) | Tall | Namatanai Tall Etalat | PNG | 0.994 | 0.006 | 0.410 | 0.021 | 0.017 | 0.548 | 0.004 |
| 355 | 10(A4d) | Tall | Markham Valley Tall | PNG | 0.993 | 0.007 | 0.021 | 0.072 | 0.015 | 0.888 | 0.005 |
| 356 | 10(A4d) | Tall | Markham Valley Tall | PNG | 0.994 | 0.006 | 0.023 | 0.283 | 0.037 | 0.652 | 0.004 |
| 357 | 10(A4d) | Tall | Markham Valley Tall | PNG | 0.993 | 0.007 | 0.016 | 0.008 | 0.011 | 0.961 | 0.004 |
| 358 | 10(A4d) | Tall | Markham Valley Tall | PNG | 0.993 | 0.007 | 0.017 | 0.045 | 0.009 | 0.925 | 0.004 |
| 359 | 10(A4d) | Tall | Markham Valley Tall | PNG | 0.995 | 0.005 | 0.060 | 0.151 | 0.022 | 0.762 | 0.004 |
| 360 | 10(A4d) | Tall | Markham Valley Tall Markham Farm | PNG | 0.992 | 0.008 | 0.063 | 0.016 | 0.035 | 0.880 | 0.005 |
| 361 | 10(A4d) | Tall | Markham Valley Tall Markham Farm | PNG | 0.987 | 0.013 | 0.045 | 0.035 | 0.013 | 0.899 | 0.009 |
| 362 | 10(A4d) | Tall | Markham Valley Tall Markham Farm | PNG | 0.990 | 0.010 | 0.098 | 0.062 | 0.042 | 0.791 | 0.007 |
| 363 | 10(A4d) | Tall | Markham Valley Tall Markham Farm | PNG | 0.985 | 0.015 | 0.009 | 0.031 | 0.021 | 0.932 | 0.008 |
| 364 | 10(A4d) | Tall | Markham Valley Tall Markham Farm | PNG | 0.983 | 0.017 | 0.115 | 0.019 | 0.021 | 0.833 | 0.011 |
| 365 | 10(A4d) | Tall | Markham Valley Tall Liara Village | PNG | 0.987 | 0.013 | 0.062 | 0.015 | 0.007 | 0.908 | 0.007 |
| 366 | 10(A4d) | Tall | Markham Valley Tall Liara Village | PNG | 0.994 | 0.006 | 0.129 | 0.403 | 0.014 | 0.448 | 0.005 |
| 367 | 10(A4d) | Tall | Markham Valley Tall Liara Village | PNG | 0.989 | 0.011 | 0.106 | 0.219 | 0.010 | 0.657 | 0.008 |
| 368 | 10(A4d) | Tall | Markham Valley Tall Liara Village | PNG | 0.994 | 0.006 | 0.116 | 0.088 | 0.019 | 0.771 | 0.005 |
| 369 | 10(A4d) | Tall | Markham Valley Tall Liara Village | PNG | 0.993 | 0.007 | 0.155 | 0.175 | 0.024 | 0.640 | 0.006 |
| 370 | 10(A4d) | Tall | Markham Valley Tall Markham Farm | PNG | 0.992 | 0.008 | 0.021 | 0.281 | 0.014 | 0.679 | 0.006 |
| 371 | 10(A4d) | Tall | Markham Valley Tall Markham Farm | PNG | 0.995 | 0.005 | 0.077 | 0.030 | 0.012 | 0.877 | 0.004 |
| 372 | 10(A4d) | Tall | Markham Valley Tall Markham Farm | PNG | 0.994 | 0.006 | 0.085 | 0.021 | 0.057 | 0.833 | 0.004 |
| 373 | 10(A4d) | Tall | Markham Valley Tall Markham Farm | PNG | 0.994 | 0.006 | 0.622 | 0.043 | 0.184 | 0.146 | 0.004 |
| 374 | 10(A4d) | Tall | Markham Valley Tall Markham Farm | PNG | 0.985 | 0.015 | 0.020 | 0.009 | 0.016 | 0.948 | 0.007 |
| 375 | 10(A4d) | Tall | Markham Valley Tall | PNG | 0.993 | 0.007 | 0.495 | 0.021 | 0.395 | 0.084 | 0.005 |
| 376 | 11(A4e) | Tall | Solomon Island Tall | SLB | 0.991 | 0.009 | 0.797 | 0.018 | 0.039 | 0.139 | 0.006 |
| 377 | 11(A4e) | Tall | Rennell Island Tall | SLB | 0.995 | 0.005 | 0.916 | 0.020 | 0.024 | 0.037 | 0.004 |
| 378 | 11(A4e) | Tall | Rennell Island Tall | SLB | 0.993 | 0.007 | 0.539 | 0.032 | 0.021 | 0.402 | 0.005 |
| 379 | 11(A4e) | Tall | Rennell Island Tall | SLB | 0.995 | 0.005 | 0.481 | 0.131 | 0.025 | 0.359 | 0.004 |
| 380 | 11(A4e) | Tall | Rennell Island Tall | SLB | 0.991 | 0.009 | 0.904 | 0.016 | 0.039 | 0.035 | 0.006 |
| 381 | 11(A4e) | Tall | Rennell Island Tall | SLB | 0.995 | 0.005 | 0.444 | 0.452 | 0.045 | 0.054 | 0.004 |
| 382 | 11(A4e) | Tall | Rennell Island Tall | SLB | 0.993 | 0.007 | 0.948 | 0.012 | 0.019 | 0.017 | 0.005 |
| 383 | 11(A4e) | Tall | Rennell Island Tall | SLB | 0.993 | 0.007 | 0.950 | 0.013 | 0.014 | 0.019 | 0.005 |
| 384 | 11(A4e) | Tall | Solomon Island Tall | SLB | 0.995 | 0.005 | 0.452 | 0.046 | 0.305 | 0.194 | 0.004 |
| 385 | 11(A4e) | Tall | Solomon Island Tall | SLB | 0.995 | 0.005 | 0.646 | 0.023 | 0.197 | 0.130 | 0.004 |
| 386 | 11(A4e) | Tall | Solomon Island Tall | SLB | 0.992 | 0.008 | 0.419 | 0.017 | 0.234 | 0.325 | 0.006 |
| 387 | 11(A4e) | Tall | Solomon Island Tall | SLB | 0.995 | 0.005 | 0.362 | 0.070 | 0.204 | 0.360 | 0.004 |
| 388 | 11(A4e) | Tall | Solomon Island Tall | SLB | 0.994 | 0.006 | 0.419 | 0.015 | 0.012 | 0.549 | 0.005 |
| 389 | 11(A4e) | Tall | Rennell Island Tall | SLB | 0.993 | 0.007 | 0.938 | 0.015 | 0.019 | 0.022 | 0.005 |
| 390 | 11(A4e) | Tall | Rennell Island Tall | SLB | 0.991 | 0.009 | 0.895 | 0.015 | 0.022 | 0.060 | 0.007 |
| 391 | 11(A4e) | Tall | Rennell Island Tall | SLB | 0.995 | 0.005 | 0.901 | 0.037 | 0.019 | 0.039 | 0.004 |
| 392 | 11(A4e) | Tall | Rennell Island Tall | SLB | 0.994 | 0.006 | 0.874 | 0.010 | 0.038 | 0.074 | 0.005 |
| 393 | 11(A4e) | Tall | Rennell Island Tall | SLB | 0.994 | 0.006 | 0.933 | 0.022 | 0.016 | 0.024 | 0.005 |
| 394 | 11(A4e) | Tall | Vanuatu Tall | VUT | 0.991 | 0.009 | 0.139 | 0.017 | 0.511 | 0.317 | 0.017 |
| 395 | 11(A4e) | Tall | Vanuatu Tall | VUT | 0.994 | 0.006 | 0.784 | 0.014 | 0.008 | 0.188 | 0.005 |
| 396 | 11(A4e) | Tall | Vanuatu Tall | VUT | 0.995 | 0.005 | 0.868 | 0.027 | 0.034 | 0.068 | 0.004 |
| 397 | 11(A4e) | Tall | Vanuatu Tall | VUT | 0.994 | 0.006 | 0.135 | 0.122 | 0.197 | 0.541 | 0.005 |
| 398 | 11(A4e) | Tall | Vanuatu Tall | VUT | 0.982 | 0.018 | 0.216 | 0.012 | 0.173 | 0.583 | 0.016 |
| 399 | 11(A4e) | Tall | New Caledonia Tall | NCL | 0.980 | 0.020 | 0.657 | 0.008 | 0.018 | 0.306 | 0.011 |
| 400 | 11(A4e) | Tall | New Caledonia Tall | NCL | 0.990 | 0.010 | 0.665 | 0.006 | 0.008 | 0.314 | 0.006 |
| 401 | 11(A4e) | Tall | New Caledonia Tall | NCL | 0.959 | 0.041 | 0.043 | 0.007 | 0.011 | 0.915 | 0.024 |
| 402 | 11(A4e) | Tall | New Caledonia Tall | NCL | 0.953 | 0.047 | 0.097 | 0.005 | 0.009 | 0.871 | 0.017 |
| 403 | 11(A4e) | Tall | New Caledonia Tall | NCL | 0.993 | 0.007 | 0.402 | 0.013 | 0.037 | 0.544 | 0.005 |
| 404 | 11(A4e) | Tall | New Caledonia Tall | NCL | 0.952 | 0.048 | 0.907 | 0.007 | 0.013 | 0.051 | 0.022 |
| 405 | 11(A4e) | Tall | New Caledonia Tall | NCL | 0.990 | 0.010 | 0.774 | 0.023 | 0.035 | 0.162 | 0.006 |
| 406 | 11(A4e) | Tall | Vanuatu Tall | VUT | 0.995 | 0.005 | 0.930 | 0.007 | 0.012 | 0.048 | 0.004 |
| 407 | 11(A4e) | Tall | Vanuatu Tall | VUT | 0.992 | 0.008 | 0.666 | 0.006 | 0.010 | 0.312 | 0.006 |
| 408 | 11(A4e) | Tall | Vanuatu Tall | VUT | 0.977 | 0.023 | 0.947 | 0.014 | 0.013 | 0.016 | 0.011 |
| 409 | 11(A4e) | Tall | Vanuatu Tall | VUT | 0.991 | 0.009 | 0.274 | 0.050 | 0.081 | 0.588 | 0.007 |
| 410 | 11(A4e) | Tall | Vanuatu Tall | VUT | 0.993 | 0.007 | 0.590 | 0.200 | 0.059 | 0.145 | 0.006 |
| 411 | 11(A4e) | Tall | Vanuatu Tall | VUT | 0.993 | 0.007 | 0.077 | 0.019 | 0.010 | 0.889 | 0.005 |
| 412 | 11(A4e) | Tall | Vanuatu Tall | VUT | 0.993 | 0.007 | 0.077 | 0.039 | 0.043 | 0.835 | 0.005 |
| 413 | 11(A4e) | Tall | Vanuatu Tall | VUT | 0.892 | 0.108 | 0.100 | 0.009 | 0.012 | 0.798 | 0.081 |
| 414 | 11(A4e) | Tall | Vanuatu Tall | VUT | 0.995 | 0.005 | 0.915 | 0.025 | 0.007 | 0.048 | 0.004 |
| 415 | 11(A4e) | Tall | Vanuatu Tall | VUT | 0.991 | 0.009 | 0.941 | 0.011 | 0.013 | 0.030 | 0.005 |
| 416 | 11(A4e) | Tall | Vanuatu Tall | VUT | 0.980 | 0.020 | 0.843 | 0.011 | 0.050 | 0.086 | 0.010 |
| 417 | 11(A4e) | Tall | Vanuatu Tall | VUT | 0.992 | 0.008 | 0.425 | 0.019 | 0.193 | 0.358 | 0.006 |
| 418 | 11(A4e) | Tall | Vanuatu Tall | VUT | 0.994 | 0.006 | 0.670 | 0.040 | 0.008 | 0.277 | 0.005 |
| 419 | 11(A4e) | Tall | Vanuatu Tall | VUT | 0.993 | 0.007 | 0.904 | 0.018 | 0.048 | 0.025 | 0.005 |
| 420 | 11(A4e) | Tall | Vanuatu Tall | VUT | 0.949 | 0.051 | 0.053 | 0.039 | 0.050 | 0.844 | 0.014 |
| 421 | 11(A4e) | Tall | Vanuatu Tall | VUT | 0.993 | 0.007 | 0.562 | 0.125 | 0.015 | 0.292 | 0.006 |
| 422 | 11(A4e) | Tall | Vanuatu Tall | VUT | 0.865 | 0.135 | 0.079 | 0.020 | 0.107 | 0.667 | 0.127 |
| 423 | 11(A4e) | Tall | Vanuatu Tall | VUT | 0.986 | 0.014 | 0.915 | 0.007 | 0.009 | 0.061 | 0.008 |
| 424 | 11(A4e) | Tall | Vanuatu Tall | VUT | 0.989 | 0.011 | 0.777 | 0.013 | 0.010 | 0.191 | 0.009 |
| 425 | 11(A4e) | Tall | Vanuatu Tall | VUT | 0.971 | 0.029 | 0.475 | 0.025 | 0.020 | 0.461 | 0.019 |
| 426 | 11(A4e) | Tall | Vanuatu Tall | VUT | 0.967 | 0.033 | 0.475 | 0.127 | 0.024 | 0.350 | 0.024 |
| 427 | 11(A4e) | Tall | Vanuatu Tall | VUT | 0.986 | 0.014 | 0.933 | 0.010 | 0.022 | 0.026 | 0.010 |
| 428 | 11(A4e) | Tall | Vanuatu Tall | VUT | 0.995 | 0.005 | 0.911 | 0.020 | 0.033 | 0.033 | 0.003 |
| 429 | 11(A4e) | Tall | Vanuatu Tall | VUT | 0.994 | 0.006 | 0.763 | 0.009 | 0.038 | 0.186 | 0.004 |
| 430 | 11(A4e) | Tall | Vanuatu Tall | VUT | 0.994 | 0.006 | 0.905 | 0.031 | 0.038 | 0.020 | 0.005 |
| 431 | 11(A4e) | Tall | Vanuatu Tall | VUT | 0.993 | 0.007 | 0.502 | 0.034 | 0.210 | 0.248 | 0.005 |
| 432 | 11(A4e) | Tall | Vanuatu Tall | VUT | 0.990 | 0.010 | 0.875 | 0.012 | 0.036 | 0.070 | 0.007 |
| 433 | 11(A4e) | Tall | Vanuatu Tall | VUT | 0.993 | 0.007 | 0.932 | 0.008 | 0.014 | 0.041 | 0.006 |
| 434 | 11(A4e) | Tall | Vanuatu Tall | VUT | 0.994 | 0.006 | 0.263 | 0.010 | 0.142 | 0.580 | 0.005 |
| 435 | 11(A4e) | Tall | Vanuatu Tall | VUT | 0.941 | 0.059 | 0.313 | 0.213 | 0.147 | 0.267 | 0.060 |
| 436 | 11(A4e) | Tall | Vanuatu Tall | VUT | 0.992 | 0.008 | 0.944 | 0.012 | 0.019 | 0.022 | 0.004 |
| 437 | 11(A4e) | Tall | Vanuatu Tall | VUT | 0.993 | 0.007 | 0.169 | 0.011 | 0.027 | 0.789 | 0.005 |
| 438 | 11(A4e) | Tall | Vanuatu Tall | VUT | 0.995 | 0.005 | 0.772 | 0.170 | 0.023 | 0.031 | 0.004 |
| 439 | 11(A4e) | Tall | Vanuatu Tall | VUT | 0.995 | 0.005 | 0.867 | 0.017 | 0.016 | 0.096 | 0.004 |
| 440 | 11(A4e) | Tall | Vanuatu Tall | VUT | 0.992 | 0.008 | 0.859 | 0.017 | 0.011 | 0.108 | 0.005 |
| 441 | 11(A4e) | Tall | Vanuatu Tall | VUT | 0.994 | 0.006 | 0.709 | 0.055 | 0.038 | 0.193 | 0.004 |
| 442 | 11(A4e) | Tall | Vanuatu Tall | VUT | 0.995 | 0.005 | 0.324 | 0.020 | 0.009 | 0.643 | 0.004 |
| 443 | 11(A4e) | Tall | Vanuatu Tall | VUT | 0.992 | 0.008 | 0.946 | 0.008 | 0.015 | 0.027 | 0.005 |
| 444 | 11(A4e) | Tall | Vanuatu Tall | VUT | 0.993 | 0.007 | 0.872 | 0.011 | 0.015 | 0.097 | 0.005 |
| 445 | 11(A4e) | Tall | Vanuatu Tall | VUT | 0.995 | 0.005 | 0.797 | 0.089 | 0.038 | 0.071 | 0.004 |
| 446 | 11(A4e) | Tall | Vanuatu Tall | VUT | 0.995 | 0.005 | 0.501 | 0.471 | 0.010 | 0.014 | 0.004 |
| 447 | 11(A4e) | Tall | Vanuatu Tall | VUT | 0.955 | 0.045 | 0.877 | 0.023 | 0.006 | 0.046 | 0.047 |
| 448 | 11(A4e) | Tall | Vanuatu Tall | VUT | 0.994 | 0.006 | 0.825 | 0.034 | 0.038 | 0.098 | 0.005 |
| 449 | 11(A4e) | Tall | Vanuatu Tall | VUT | 0.995 | 0.005 | 0.712 | 0.155 | 0.112 | 0.017 | 0.004 |
| 450 | 11(A4e) | Tall | Vanuatu Tall | VUT | 0.986 | 0.014 | 0.689 | 0.177 | 0.064 | 0.057 | 0.013 |
| 451 | 11(A4e) | Tall | Vanuatu Tall | VUT | 0.994 | 0.006 | 0.930 | 0.008 | 0.015 | 0.042 | 0.005 |
| 452 | 11(A4e) | Tall | Vanuatu Tall | VUT | 0.995 | 0.005 | 0.699 | 0.044 | 0.043 | 0.209 | 0.004 |
| 453 | 11(A4e) | Tall | Vanuatu Tall | VUT | 0.995 | 0.005 | 0.317 | 0.019 | 0.009 | 0.650 | 0.004 |
| 454 | 11(A4e) | Tall | Vanuatu Tall | VUT | 0.982 | 0.018 | 0.934 | 0.014 | 0.025 | 0.020 | 0.008 |
| 455 | 11(A4e) | Tall | Vanuatu Tall | VUT | 0.986 | 0.014 | 0.576 | 0.062 | 0.341 | 0.012 | 0.009 |
| 456 | 11(A4e) | Tall | Vanuatu Tall | VUT | 0.994 | 0.006 | 0.361 | 0.087 | 0.322 | 0.224 | 0.005 |
| 457 | 11(A4e) | Tall | Vanuatu Tall | VUT | 0.993 | 0.007 | 0.032 | 0.022 | 0.062 | 0.879 | 0.005 |
| 458 | 11(A4e) | Tall | Vanuatu Tall | VUT | 0.993 | 0.007 | 0.645 | 0.028 | 0.022 | 0.299 | 0.005 |
| 459 | 11(A4e) | Tall | Vanuatu Tall | VUT | 0.990 | 0.010 | 0.899 | 0.010 | 0.053 | 0.032 | 0.006 |
| 460 | 11(A4e) | Tall | Vanuatu Tall | VUT | 0.991 | 0.009 | 0.794 | 0.031 | 0.117 | 0.051 | 0.007 |
| 461 | 11(A4e) | Tall | Vanuatu Tall | VUT | 0.991 | 0.009 | 0.094 | 0.041 | 0.016 | 0.843 | 0.006 |
| 462 | 11(A4e) | Tall | Vanuatu Tall | VUT | 0.992 | 0.008 | 0.442 | 0.093 | 0.234 | 0.225 | 0.006 |
| 463 | 11(A4e) | Tall | Vanuatu Tall | VUT | 0.995 | 0.005 | 0.774 | 0.179 | 0.023 | 0.020 | 0.004 |
| 464 | 11(A4e) | Tall | Vanuatu Tall | VUT | 0.995 | 0.005 | 0.947 | 0.016 | 0.013 | 0.019 | 0.004 |
| 465 | 11(A4e) | Tall | Vanuatu Tall | VUT | 0.988 | 0.012 | 0.390 | 0.026 | 0.267 | 0.307 | 0.010 |
| 466 | 11(A4e) | Tall | Vanuatu Tall | VUT | 0.995 | 0.005 | 0.939 | 0.011 | 0.018 | 0.029 | 0.004 |
| 467 | 11(A4e) | Tall | Vanuatu Tall | VUT | 0.974 | 0.026 | 0.621 | 0.008 | 0.019 | 0.334 | 0.016 |
| 468 | 11(A4e) | Tall | Vanuatu Tall | VUT | 0.995 | 0.005 | 0.139 | 0.025 | 0.054 | 0.778 | 0.004 |
| 469 | 11(A4e) | Tall | Vanuatu Tall | VUT | 0.995 | 0.005 | 0.918 | 0.014 | 0.011 | 0.053 | 0.004 |
| 470 | 11(A4e) | Tall | Vanuatu Tall | VUT | 0.995 | 0.005 | 0.502 | 0.062 | 0.201 | 0.231 | 0.004 |
| 471 | 11(A4e) | Tall | Vanuatu Tall | VUT | 0.990 | 0.010 | 0.786 | 0.138 | 0.010 | 0.058 | 0.009 |
| 472 | 11(A4e) | Tall | Vanuatu Tall | VUT | 0.994 | 0.006 | 0.480 | 0.218 | 0.031 | 0.267 | 0.005 |
| 473 | 11(A4e) | Tall | Vanuatu Tall | VUT | 0.957 | 0.043 | 0.134 | 0.018 | 0.105 | 0.728 | 0.016 |
| 474 | 11(A4e) | Tall | Vanuatu Tall | VUT | 0.995 | 0.005 | 0.528 | 0.321 | 0.026 | 0.122 | 0.004 |
| 475 | 11(A4e) | Tall | Vanuatu Tall | VUT | 0.993 | 0.007 | 0.239 | 0.022 | 0.036 | 0.698 | 0.005 |
| 476 | 11(A4e) | Tall | Vanuatu Tall | VUT | 0.951 | 0.049 | 0.475 | 0.022 | 0.067 | 0.410 | 0.027 |
| 477 | 11(A4e) | Tall | Vanuatu Tall | VUT | 0.976 | 0.024 | 0.691 | 0.007 | 0.030 | 0.257 | 0.016 |
| 478 | 11(A4e) | Tall | Vanuatu Tall | VUT | 0.977 | 0.023 | 0.027 | 0.007 | 0.016 | 0.941 | 0.009 |
| 479 | 11(A4e) | Tall | Vanuatu Tall | VUT | 0.995 | 0.005 | 0.779 | 0.053 | 0.045 | 0.119 | 0.004 |
| 480 | 11(A4e) | Tall | Vanuatu Tall | VUT | 0.995 | 0.005 | 0.912 | 0.005 | 0.009 | 0.070 | 0.004 |
| 481 | 11(A4e) | Tall | Vanuatu Tall | VUT | 0.995 | 0.005 | 0.752 | 0.061 | 0.033 | 0.150 | 0.004 |
| 482 | 11(A4e) | Tall | Vanuatu Tall | VUT | 0.995 | 0.005 | 0.672 | 0.212 | 0.014 | 0.099 | 0.004 |
| 483 | 11(A4e) | Tall | Vanuatu Tall | VUT | 0.987 | 0.013 | 0.528 | 0.036 | 0.210 | 0.217 | 0.009 |
| 484 | 11(A4e) | Tall | Vanuatu Tall | VUT | 0.991 | 0.009 | 0.894 | 0.007 | 0.022 | 0.070 | 0.007 |
| 485 | 11(A4e) | Tall | Vanuatu Tall | VUT | 0.994 | 0.006 | 0.328 | 0.057 | 0.018 | 0.592 | 0.005 |
| 486 | 11(A4e) | Tall | Vanuatu Tall | VUT | 0.993 | 0.007 | 0.969 | 0.005 | 0.007 | 0.015 | 0.005 |
| 487 | 11(A4e) | Tall | Vanuatu Tall | VUT | 0.964 | 0.036 | 0.890 | 0.027 | 0.012 | 0.051 | 0.020 |
| 488 | 11(A4e) | Tall | Vanuatu Tall | VUT | 0.995 | 0.005 | 0.214 | 0.022 | 0.472 | 0.289 | 0.004 |
| 489 | 11(A4e) | Tall | Vanuatu Tall | VUT | 0.995 | 0.005 | 0.768 | 0.061 | 0.126 | 0.041 | 0.004 |
| 490 | 11(A4e) | Tall | Vanuatu Tall | VUT | 0.993 | 0.007 | 0.973 | 0.005 | 0.006 | 0.011 | 0.005 |
| 491 | 11(A4e) | Tall | Vanuatu Tall | VUT | 0.989 | 0.011 | 0.061 | 0.014 | 0.016 | 0.903 | 0.006 |
| 492 | 11(A4e) | Tall | Vanuatu Tall | VUT | 0.995 | 0.005 | 0.389 | 0.020 | 0.561 | 0.025 | 0.004 |
| 493 | 11(A4e) | Tall | Vanuatu Tall | VUT | 0.995 | 0.005 | 0.878 | 0.044 | 0.034 | 0.041 | 0.004 |
| 494 | 11(A4e) | Tall | Vanuatu Tall | VUT | 0.995 | 0.005 | 0.048 | 0.039 | 0.861 | 0.048 | 0.004 |
| 495 | 11(A4e) | Tall | Vanuatu Tall | VUT | 0.980 | 0.020 | 0.843 | 0.008 | 0.006 | 0.122 | 0.022 |
| 496 | 11(A4e) | Tall | Vanuatu Tall | VUT | 0.993 | 0.007 | 0.770 | 0.022 | 0.027 | 0.177 | 0.005 |
| 497 | 11(A4e) | Tall | Vanuatu Tall | VUT | 0.993 | 0.007 | 0.757 | 0.007 | 0.015 | 0.215 | 0.006 |
| 498 | 11(A4e) | Tall | Vanuatu Tall | VUT | 0.995 | 0.005 | 0.086 | 0.046 | 0.038 | 0.826 | 0.004 |
| 499 | 11(A4e) | Tall | Vanuatu Tall | VUT | 0.995 | 0.005 | 0.916 | 0.019 | 0.014 | 0.046 | 0.005 |
| 500 | 11(A4e) | Tall | Vanuatu Tall | VUT | 0.995 | 0.005 | 0.100 | 0.006 | 0.015 | 0.875 | 0.004 |
| 501 | 11(A4e) | Tall | Vanuatu Tall | VUT | 0.980 | 0.020 | 0.930 | 0.010 | 0.032 | 0.018 | 0.010 |
| 502 | 11(A4e) | Tall | Vanuatu Tall | VUT | 0.992 | 0.008 | 0.864 | 0.017 | 0.012 | 0.102 | 0.005 |
| 503 | 11(A4e) | Tall | Vanuatu Tall | VUT | 0.995 | 0.005 | 0.956 | 0.014 | 0.013 | 0.013 | 0.004 |
| 504 | 11(A4e) | Tall | Vanuatu Tall | VUT | 0.996 | 0.004 | 0.957 | 0.011 | 0.015 | 0.013 | 0.003 |
| 505 | 11(A4e) | Tall | Vanuatu Tall | VUT | 0.995 | 0.005 | 0.821 | 0.008 | 0.051 | 0.116 | 0.004 |
| 506 | 11(A4e) | Tall | Vanuatu Tall | VUT | 0.995 | 0.005 | 0.943 | 0.025 | 0.011 | 0.017 | 0.004 |
| 507 | 11(A4e) | Tall | Vanuatu Tall | VUT | 0.993 | 0.007 | 0.728 | 0.052 | 0.046 | 0.169 | 0.006 |
| 508 | 11(A4e) | Tall | Vanuatu Tall | VUT | 0.931 | 0.069 | 0.010 | 0.010 | 0.012 | 0.949 | 0.019 |
| 509 | 11(A4e) | Tall | Vanuatu Tall | VUT | 0.994 | 0.006 | 0.344 | 0.464 | 0.017 | 0.170 | 0.005 |
| 510 | 11(A4e) | Tall | Vanuatu Tall | VUT | 0.995 | 0.005 | 0.839 | 0.020 | 0.052 | 0.085 | 0.004 |
| 511 | 11(A4e) | Tall | Vanuatu Tall | VUT | 0.993 | 0.007 | 0.847 | 0.028 | 0.085 | 0.033 | 0.006 |
| 512 | 11(A4e) | Tall | Vanuatu Tall | VUT | 0.941 | 0.059 | 0.418 | 0.021 | 0.022 | 0.509 | 0.031 |
| 513 | 11(A4e) | Tall | Vanuatu Tall | VUT | 0.975 | 0.025 | 0.542 | 0.050 | 0.047 | 0.348 | 0.014 |
| 514 | 11(A4e) | Tall | Vanuatu Tall | VUT | 0.995 | 0.005 | 0.451 | 0.237 | 0.044 | 0.265 | 0.004 |
| 515 | 11(A4e) | Tall | Vanuatu Tall | VUT | 0.987 | 0.013 | 0.921 | 0.012 | 0.010 | 0.047 | 0.010 |
| 516 | 11(A4e) | Tall | Vanuatu Tall | VUT | 0.995 | 0.005 | 0.671 | 0.262 | 0.016 | 0.047 | 0.004 |
| 517 | 11(A4e) | Tall | Vanuatu Tall | VUT | 0.995 | 0.005 | 0.820 | 0.018 | 0.009 | 0.149 | 0.004 |
| 518 | 11(A4e) | Tall | Vanuatu Tall | VUT | 0.994 | 0.006 | 0.901 | 0.005 | 0.009 | 0.081 | 0.004 |
| 519 | 11(A4e) | Tall | Vanuatu Tall | VUT | 0.994 | 0.006 | 0.235 | 0.090 | 0.117 | 0.554 | 0.004 |
| 520 | 11(A4e) | Tall | Vanuatu Tall | VUT | 0.987 | 0.013 | 0.546 | 0.072 | 0.284 | 0.084 | 0.013 |
| 521 | 11(A4e) | Tall | Vanuatu Tall | VUT | 0.995 | 0.005 | 0.544 | 0.011 | 0.410 | 0.031 | 0.004 |
| 522 | 11(A4e) | Tall | Vanuatu Tall | VUT | 0.995 | 0.005 | 0.897 | 0.031 | 0.012 | 0.056 | 0.004 |
| 523 | 11(A4e) | Tall | Vanuatu Tall | VUT | 0.995 | 0.005 | 0.886 | 0.028 | 0.058 | 0.025 | 0.004 |
| 524 | 11(A4e) | Tall | Vanuatu Tall | VUT | 0.993 | 0.007 | 0.195 | 0.042 | 0.081 | 0.677 | 0.005 |
| 525 | 11(A4e) | Tall | Vanuatu Tall | VUT | 0.995 | 0.005 | 0.918 | 0.035 | 0.005 | 0.037 | 0.005 |
| 526 | 11(A4e) | Tall | Vanuatu Tall | VUT | 0.994 | 0.006 | 0.819 | 0.062 | 0.014 | 0.100 | 0.005 |
| 527 | 11(A4e) | Tall | Vanuatu Tall | VUT | 0.995 | 0.005 | 0.714 | 0.028 | 0.077 | 0.177 | 0.004 |
| 528 | 11(A4e) | Tall | Vanuatu Tall | VUT | 0.995 | 0.005 | 0.468 | 0.020 | 0.126 | 0.383 | 0.004 |
| 529 | 11(A4e) | Tall | Vanuatu Tall | VUT | 0.989 | 0.011 | 0.639 | 0.013 | 0.122 | 0.218 | 0.008 |
| 530 | 11(A4e) | Tall | Vanuatu Tall | VUT | 0.990 | 0.010 | 0.312 | 0.049 | 0.024 | 0.609 | 0.007 |
| 531 | 11(A4e) | Tall | Vanuatu Tall | VUT | 0.955 | 0.045 | 0.876 | 0.024 | 0.007 | 0.045 | 0.048 |
| 532 | 11(A4e) | Tall | Vanuatu Tall | VUT | 0.944 | 0.056 | 0.877 | 0.020 | 0.056 | 0.013 | 0.034 |
| 533 | 11(A4e) | Tall | Vanuatu Tall | VUT | 0.995 | 0.005 | 0.722 | 0.108 | 0.129 | 0.037 | 0.004 |
| 534 | 11(A4e) | Tall | Vanuatu Tall | VUT | 0.991 | 0.009 | 0.946 | 0.012 | 0.006 | 0.029 | 0.007 |
| 535 | 11(A4e) | Tall | Vanuatu Tall | VUT | 0.994 | 0.006 | 0.623 | 0.053 | 0.198 | 0.121 | 0.005 |
| 536 | 11(A4e) | Tall | Vanuatu Tall | VUT | 0.994 | 0.006 | 0.251 | 0.040 | 0.094 | 0.609 | 0.005 |
| 537 | 11(A4e) | Tall | Vanuatu Tall | VUT | 0.906 | 0.094 | 0.178 | 0.037 | 0.467 | 0.225 | 0.093 |
| 538 | 11(A4e) | Tall | Vanuatu Tall | VUT | 0.995 | 0.005 | 0.931 | 0.012 | 0.019 | 0.034 | 0.004 |
| 539 | 11(A4e) | Tall | Vanuatu Tall | VUT | 0.983 | 0.017 | 0.630 | 0.010 | 0.028 | 0.322 | 0.009 |
| 540 | 11(A4e) | Tall | Vanuatu Tall | VUT | 0.991 | 0.009 | 0.365 | 0.020 | 0.012 | 0.597 | 0.006 |
| 541 | 11(A4e) | Tall | Vanuatu Tall | VUT | 0.988 | 0.012 | 0.660 | 0.018 | 0.099 | 0.214 | 0.009 |
| 542 | 11(A4e) | Tall | Vanuatu Tall | VUT | 0.989 | 0.011 | 0.695 | 0.016 | 0.010 | 0.269 | 0.010 |
| 543 | 11(A4e) | Tall | Vanuatu Tall | VUT | 0.988 | 0.012 | 0.071 | 0.557 | 0.144 | 0.217 | 0.011 |
| 544 | 11(A4e) | Tall | Vanuatu Tall | VUT | 0.994 | 0.006 | 0.669 | 0.235 | 0.016 | 0.073 | 0.005 |
| 545 | 11(A4e) | Tall | Vanuatu Tall | VUT | 0.990 | 0.010 | 0.409 | 0.026 | 0.029 | 0.530 | 0.007 |
| 546 | 11(A4e) | Tall | Vanuatu Tall | VUT | 0.994 | 0.006 | 0.664 | 0.158 | 0.052 | 0.122 | 0.005 |
| 547 | 11(A4e) | Tall | Vanuatu Tall | VUT | 0.993 | 0.007 | 0.273 | 0.016 | 0.027 | 0.679 | 0.005 |
| 548 | 11(A4e) | Tall | Vanuatu Tall | VUT | 0.994 | 0.006 | 0.885 | 0.011 | 0.025 | 0.074 | 0.004 |
| 549 | 11(A4e) | Tall | Vanuatu Tall | VUT | 0.990 | 0.010 | 0.523 | 0.009 | 0.042 | 0.420 | 0.007 |
| 550 | 11(A4e) | Tall | Vanuatu Tall | VUT | 0.994 | 0.006 | 0.965 | 0.008 | 0.005 | 0.017 | 0.005 |
| 551 | 11(A4e) | Tall | Vanuatu Tall | VUT | 0.992 | 0.008 | 0.890 | 0.009 | 0.052 | 0.043 | 0.006 |
| 552 | 11(A4e) | Tall | Vanuatu Tall | VUT | 0.995 | 0.005 | 0.776 | 0.014 | 0.020 | 0.187 | 0.004 |
| 553 | 11(A4e) | Tall | Vanuatu Tall | VUT | 0.992 | 0.008 | 0.866 | 0.008 | 0.035 | 0.085 | 0.006 |
| 554 | 11(A4e) | Tall | Vanuatu Tall | VUT | 0.995 | 0.005 | 0.920 | 0.028 | 0.027 | 0.021 | 0.004 |
| 555 | 11(A4e) | Tall | Vanuatu Tall | VUT | 0.992 | 0.008 | 0.472 | 0.010 | 0.013 | 0.500 | 0.006 |
| 556 | 11(A4e) | Tall | Vanuatu Tall | VUT | 0.974 | 0.026 | 0.919 | 0.007 | 0.014 | 0.045 | 0.014 |
| 557 | 11(A4e) | Tall | Vanuatu Tall | VUT | 0.993 | 0.007 | 0.455 | 0.056 | 0.014 | 0.469 | 0.005 |
| 558 | 11(A4e) | Tall | Vanuatu Tall | VUT | 0.989 | 0.011 | 0.052 | 0.018 | 0.039 | 0.884 | 0.007 |
| 559 | 11(A4e) | Tall | Vanuatu Tall | VUT | 0.993 | 0.007 | 0.370 | 0.054 | 0.401 | 0.168 | 0.007 |
| 560 | 11(A4e) | Tall | Vanuatu Tall | VUT | 0.995 | 0.005 | 0.952 | 0.015 | 0.011 | 0.018 | 0.004 |
| 561 | 11(A4e) | Tall | Vanuatu Tall | VUT | 0.993 | 0.007 | 0.919 | 0.021 | 0.013 | 0.042 | 0.005 |
| 562 | 11(A4e) | Tall | Vanuatu Tall | VUT | 0.995 | 0.005 | 0.943 | 0.016 | 0.016 | 0.021 | 0.004 |
| 563 | 11(A4e) | Tall | Vanuatu Tall | VUT | 0.991 | 0.009 | 0.646 | 0.308 | 0.022 | 0.017 | 0.007 |
| 564 | 11(A4e) | Tall | Vanuatu Tall | VUT | 0.993 | 0.007 | 0.431 | 0.016 | 0.020 | 0.528 | 0.005 |
| 565 | 11(A4e) | Tall | Vanuatu Tall | VUT | 0.990 | 0.010 | 0.805 | 0.018 | 0.038 | 0.131 | 0.008 |
| 566 | 11(A4e) | Tall | Vanuatu Tall | VUT | 0.994 | 0.006 | 0.648 | 0.041 | 0.086 | 0.220 | 0.005 |
| 567 | 11(A4e) | Tall | Vanuatu Tall | VUT | 0.990 | 0.010 | 0.461 | 0.123 | 0.093 | 0.314 | 0.009 |
| 568 | 11(A4e) | Tall | Vanuatu Tall | VUT | 0.995 | 0.005 | 0.320 | 0.649 | 0.016 | 0.012 | 0.004 |
| 569 | 11(A4e) | Tall | Vanuatu Tall | VUT | 0.994 | 0.006 | 0.801 | 0.030 | 0.017 | 0.147 | 0.004 |
| 570 | 11(A4e) | Tall | Vanuatu Tall | VUT | 0.992 | 0.008 | 0.497 | 0.249 | 0.026 | 0.221 | 0.007 |
| 571 | 11(A4e) | Tall | Vanuatu Tall | VUT | 0.995 | 0.005 | 0.362 | 0.324 | 0.032 | 0.277 | 0.004 |
| 572 | 11(A4e) | Tall | Vanuatu Tall | VUT | 0.986 | 0.014 | 0.075 | 0.046 | 0.029 | 0.841 | 0.009 |
| 573 | 11(A4e) | Tall | Vanuatu Tall | VUT | 0.993 | 0.007 | 0.848 | 0.038 | 0.010 | 0.099 | 0.006 |
| 574 | 11(A4e) | Tall | Vanuatu Tall | VUT | 0.993 | 0.007 | 0.904 | 0.041 | 0.009 | 0.040 | 0.006 |
| 575 | 11(A4e) | Tall | Vanuatu Tall | VUT | 0.994 | 0.006 | 0.918 | 0.011 | 0.032 | 0.035 | 0.004 |
| 576 | 11(A4e) | Tall | Vanuatu Tall | VUT | 0.991 | 0.009 | 0.922 | 0.008 | 0.015 | 0.049 | 0.006 |
| 577 | 11(A4e) | Tall | Vanuatu Tall | VUT | 0.990 | 0.010 | 0.960 | 0.008 | 0.006 | 0.019 | 0.007 |
| 578 | 11(A4e) | Tall | Vanuatu Tall | VUT | 0.983 | 0.017 | 0.441 | 0.009 | 0.022 | 0.517 | 0.010 |
| 579 | 11(A4e) | Tall | Vanuatu Tall | VUT | 0.994 | 0.006 | 0.652 | 0.101 | 0.060 | 0.182 | 0.005 |
| 580 | 11(A4e) | Tall | Vanuatu Tall | VUT | 0.992 | 0.008 | 0.278 | 0.019 | 0.075 | 0.623 | 0.006 |
| 581 | 11(A4e) | Tall | Vanuatu Tall | VUT | 0.933 | 0.067 | 0.581 | 0.007 | 0.057 | 0.311 | 0.044 |
| 582 | 11(A4e) | Tall | Vanuatu Tall | VUT | 0.949 | 0.051 | 0.423 | 0.010 | 0.043 | 0.499 | 0.026 |
| 583 | 11(A4e) | Tall | Vanuatu Tall | VUT | 0.989 | 0.011 | 0.516 | 0.089 | 0.012 | 0.376 | 0.008 |
| 584 | 11(A4e) | Tall | Vanuatu Tall | VUT | 0.994 | 0.006 | 0.907 | 0.012 | 0.018 | 0.058 | 0.005 |
| 585 | 11(A4e) | Tall | Vanuatu Tall | VUT | 0.993 | 0.007 | 0.783 | 0.048 | 0.087 | 0.077 | 0.005 |
| 586 | 11(A4e) | Tall | Vanuatu Tall | VUT | 0.992 | 0.008 | 0.035 | 0.070 | 0.840 | 0.049 | 0.006 |
| 587 | 11(A4e) | Tall | Vanuatu Tall | VUT | 0.672 | 0.328 | 0.339 | 0.030 | 0.253 | 0.078 | 0.300 |
| 588 | 11(A4e) | Tall | Vanuatu Tall | VUT | 0.830 | 0.170 | 0.750 | 0.039 | 0.024 | 0.024 | 0.163 |
| 589 | 11(A4e) | Tall | Vanuatu Tall | VUT | 0.988 | 0.012 | 0.561 | 0.377 | 0.026 | 0.026 | 0.010 |
| 590 | 11(A4e) | Tall | Vanuatu Tall | VUT | 0.993 | 0.007 | 0.287 | 0.028 | 0.018 | 0.662 | 0.005 |
| 591 | 11(A4e) | Tall | Vanuatu Tall | VUT | 0.931 | 0.069 | 0.101 | 0.013 | 0.727 | 0.125 | 0.034 |
| 592 | 11(A4e) | Tall | Vanuatu Tall | VUT | 0.931 | 0.069 | 0.050 | 0.025 | 0.016 | 0.890 | 0.019 |
| 593 | 11(A4e) | Tall | Vanuatu Tall | VUT | 0.942 | 0.058 | 0.792 | 0.020 | 0.116 | 0.038 | 0.034 |
| 594 | 11(A4e) | Tall | Vanuatu Tall | VUT | 0.739 | 0.261 | 0.663 | 0.027 | 0.061 | 0.025 | 0.223 |
| 595 | 11(A4e) | Tall | Vanuatu Tall | VUT | 0.901 | 0.099 | 0.833 | 0.022 | 0.046 | 0.038 | 0.061 |
| 596 | 11(A4e) | Tall | Vanuatu Tall | VUT | 0.897 | 0.103 | 0.864 | 0.020 | 0.017 | 0.031 | 0.069 |
| 597 | 11(A4e) | Tall | Vanuatu Tall | VUT | 0.971 | 0.029 | 0.020 | 0.012 | 0.013 | 0.944 | 0.011 |
| 598 | 11(A4e) | Tall | Vanuatu Tall | VUT | 0.993 | 0.007 | 0.686 | 0.010 | 0.050 | 0.248 | 0.005 |
| 599 | 11(A4e) | Tall | Vanuatu Tall | VUT | 0.995 | 0.005 | 0.917 | 0.008 | 0.022 | 0.049 | 0.004 |
| 600 | 11(A4e) | Tall | Vanuatu Tall | VUT | 0.994 | 0.006 | 0.875 | 0.020 | 0.015 | 0.085 | 0.004 |
| 601 | 11(A4e) | Tall | Vanuatu Tall | VUT | 0.986 | 0.014 | 0.913 | 0.008 | 0.014 | 0.056 | 0.009 |
| 602 | 11(A4e) | Tall | Vanuatu Tall | VUT | 0.993 | 0.007 | 0.663 | 0.012 | 0.029 | 0.292 | 0.005 |
| 603 | 11(A4e) | Tall | Vanuatu Tall | VUT | 0.995 | 0.005 | 0.879 | 0.011 | 0.025 | 0.081 | 0.004 |
| 604 | 11(A4e) | Tall | Vanuatu Tall | VUT | 0.855 | 0.145 | 0.826 | 0.008 | 0.029 | 0.021 | 0.116 |
| 605 | 11(A4e) | Tall | Vanuatu Tall | VUT | 0.994 | 0.006 | 0.892 | 0.014 | 0.036 | 0.052 | 0.005 |
| 606 | 11(A4e) | Tall | Vanuatu Tall | VUT | 0.992 | 0.008 | 0.785 | 0.032 | 0.030 | 0.147 | 0.006 |
| 607 | 11(A4e) | Tall | Vanuatu Tall | VUT | 0.995 | 0.005 | 0.239 | 0.032 | 0.022 | 0.703 | 0.004 |
| 608 | 11(A4e) | Tall | Vanuatu Tall | VUT | 0.995 | 0.005 | 0.957 | 0.007 | 0.015 | 0.016 | 0.004 |
| 609 | 11(A4e) | Tall | Vanuatu Tall | VUT | 0.584 | 0.416 | 0.100 | 0.028 | 0.417 | 0.134 | 0.322 |
| 610 | 11(A4e) | Tall | Vanuatu Tall | VUT | 0.992 | 0.008 | 0.893 | 0.013 | 0.025 | 0.063 | 0.006 |
| 611 | 11(A4e) | Tall | Vanuatu Tall | VUT | 0.991 | 0.009 | 0.924 | 0.028 | 0.013 | 0.029 | 0.006 |
| 612 | 11(A4e) | Tall | Vanuatu Tall | VUT | 0.993 | 0.007 | 0.932 | 0.015 | 0.017 | 0.031 | 0.005 |
| 613 | 11(A4e) | Tall | Vanuatu Tall | VUT | 0.994 | 0.006 | 0.954 | 0.009 | 0.009 | 0.023 | 0.004 |
| 614 | 11(A4e) | Tall | Vanuatu Tall | VUT | 0.991 | 0.009 | 0.719 | 0.029 | 0.051 | 0.194 | 0.007 |
| 615 | 11(A4e) | Tall | Vanuatu Tall | VUT | 0.993 | 0.007 | 0.339 | 0.044 | 0.052 | 0.559 | 0.006 |
| 616 | 11(A4e) | Tall | Vanuatu Tall | VUT | 0.993 | 0.007 | 0.955 | 0.005 | 0.017 | 0.018 | 0.005 |
| 617 | 11(A4e) | Tall | Vanuatu Tall | VUT | 0.990 | 0.010 | 0.359 | 0.013 | 0.027 | 0.595 | 0.007 |
| 618 | 11(A4e) | Tall | Vanuatu Tall | VUT | 0.993 | 0.007 | 0.724 | 0.045 | 0.045 | 0.181 | 0.006 |
| 619 | 11(A4e) | Tall | Vanuatu Tall | VUT | 0.959 | 0.041 | 0.912 | 0.024 | 0.021 | 0.031 | 0.013 |
| 620 | 11(A4e) | Tall | Vanuatu Tall | VUT | 0.995 | 0.005 | 0.907 | 0.008 | 0.024 | 0.057 | 0.004 |
| 621 | 11(A4e) | Tall | Vanuatu Tall | VUT | 0.994 | 0.006 | 0.954 | 0.014 | 0.014 | 0.014 | 0.004 |
| 622 | 11(A4e) | Tall | Vanuatu Tall | VUT | 0.986 | 0.014 | 0.874 | 0.017 | 0.011 | 0.089 | 0.009 |
| 623 | 11(A4e) | Tall | Vanuatu Tall | VUT | 0.949 | 0.051 | 0.759 | 0.012 | 0.033 | 0.170 | 0.027 |
| 624 | 11(A4e) | Tall | Vanuatu Tall | VUT | 0.994 | 0.006 | 0.904 | 0.006 | 0.016 | 0.071 | 0.004 |
| 625 | 11(A4e) | Tall | Vanuatu Tall | VUT | 0.991 | 0.009 | 0.904 | 0.005 | 0.013 | 0.071 | 0.007 |
| 626 | 11(A4e) | Tall | Vanuatu Tall | VUT | 0.987 | 0.013 | 0.945 | 0.011 | 0.021 | 0.013 | 0.009 |
| 627 | 11(A4e) | Tall | Vanuatu Tall | VUT | 0.994 | 0.006 | 0.936 | 0.018 | 0.016 | 0.024 | 0.005 |
| 628 | 11(A4e) | Tall | Vanuatu Tall | VUT | 0.984 | 0.016 | 0.791 | 0.018 | 0.115 | 0.067 | 0.010 |
| 629 | 11(A4e) | Tall | Vanuatu Tall | VUT | 0.979 | 0.021 | 0.850 | 0.007 | 0.016 | 0.113 | 0.013 |
| 630 | 11(A4e) | Tall | Vanuatu Tall | VUT | 0.995 | 0.005 | 0.952 | 0.007 | 0.009 | 0.029 | 0.004 |
| 631 | 11(A4e) | Tall | Vanuatu Tall | VUT | 0.992 | 0.008 | 0.933 | 0.006 | 0.026 | 0.030 | 0.006 |
| 632 | 11(A4e) | Tall | Vanuatu Tall | VUT | 0.983 | 0.017 | 0.513 | 0.086 | 0.083 | 0.305 | 0.013 |
| 633 | 11(A4e) | Tall | Vanuatu Tall | VUT | 0.995 | 0.005 | 0.194 | 0.074 | 0.048 | 0.679 | 0.004 |
| 634 | 11(A4e) | Tall | Vanuatu Tall | VUT | 0.994 | 0.006 | 0.844 | 0.081 | 0.022 | 0.047 | 0.005 |
| 635 | 11(A4e) | Tall | Vanuatu Tall | VUT | 0.984 | 0.016 | 0.873 | 0.031 | 0.014 | 0.072 | 0.010 |
| 636 | 11(A4e) | Tall | Vanuatu Tall | VUT | 0.988 | 0.012 | 0.894 | 0.020 | 0.028 | 0.049 | 0.009 |
| 637 | 11(A4e) | Tall | Vanuatu Tall | VUT | 0.989 | 0.011 | 0.791 | 0.024 | 0.054 | 0.123 | 0.007 |
| 638 | 11(A4e) | Tall | Vanuatu Tall | VUT | 0.995 | 0.005 | 0.760 | 0.012 | 0.033 | 0.191 | 0.004 |
| 639 | 11(A4e) | Tall | Vanuatu Tall | VUT | 0.991 | 0.009 | 0.871 | 0.018 | 0.015 | 0.089 | 0.007 |
| 640 | 11(A4e) | Tall | Vanuatu Tall | VUT | 0.995 | 0.005 | 0.955 | 0.007 | 0.016 | 0.019 | 0.004 |
| 641 | 11(A4e) | Tall | Vanuatu Tall | VUT | 0.761 | 0.239 | 0.318 | 0.026 | 0.450 | 0.022 | 0.184 |
| 642 | 11(A4e) | Tall | Vanuatu Tall | VUT | 0.991 | 0.009 | 0.931 | 0.011 | 0.027 | 0.024 | 0.007 |
| 643 | 11(A4e) | Tall | Vanuatu Tall | VUT | 0.994 | 0.006 | 0.267 | 0.036 | 0.030 | 0.662 | 0.005 |
| 644 | 11(A4e) | Tall | Vanuatu Tall | VUT | 0.993 | 0.007 | 0.785 | 0.144 | 0.018 | 0.048 | 0.006 |
| 645 | 11(A4e) | Tall | Vanuatu Tall | VUT | 0.995 | 0.005 | 0.342 | 0.014 | 0.051 | 0.589 | 0.004 |
| 646 | 11(A4e) | Tall | Vanuatu Tall | VUT | 0.994 | 0.006 | 0.892 | 0.020 | 0.026 | 0.058 | 0.004 |
| 647 | 11(A4e) | Tall | Vanuatu Tall | VUT | 0.994 | 0.006 | 0.732 | 0.071 | 0.015 | 0.177 | 0.005 |
| 648 | 11(A4e) | Tall | Vanuatu Tall | VUT | 0.991 | 0.009 | 0.818 | 0.013 | 0.034 | 0.129 | 0.006 |
| 649 | 11(A4e) | Tall | Vanuatu Tall | VUT | 0.994 | 0.006 | 0.415 | 0.020 | 0.062 | 0.498 | 0.005 |
| 650 | 11(A4e) | Tall | Vanuatu Tall | VUT | 0.996 | 0.004 | 0.959 | 0.007 | 0.013 | 0.017 | 0.003 |
| 651 | 11(A4e) | Tall | Vanuatu Tall | VUT | 0.972 | 0.028 | 0.917 | 0.011 | 0.034 | 0.024 | 0.014 |
| 652 | 11(A4e) | Tall | Vanuatu Tall | VUT | 0.995 | 0.005 | 0.877 | 0.010 | 0.052 | 0.057 | 0.004 |
| 653 | 11(A4e) | Tall | Vanuatu Tall | VUT | 0.992 | 0.008 | 0.785 | 0.021 | 0.054 | 0.134 | 0.006 |
| 654 | 11(A4e) | Tall | Vanuatu Tall | VUT | 0.993 | 0.007 | 0.871 | 0.023 | 0.076 | 0.024 | 0.005 |
| 655 | 11(A4e) | Tall | Vanuatu Tall | VUT | 0.904 | 0.096 | 0.323 | 0.013 | 0.596 | 0.026 | 0.042 |
| 656 | 11(A4e) | Tall | Vanuatu Tall | VUT | 0.992 | 0.008 | 0.950 | 0.006 | 0.019 | 0.020 | 0.006 |
| 657 | 11(A4e) | Tall | Vanuatu Tall | VUT | 0.989 | 0.011 | 0.774 | 0.101 | 0.017 | 0.100 | 0.009 |
| 658 | 11(A4e) | Tall | Vanuatu Tall | VUT | 0.994 | 0.006 | 0.063 | 0.020 | 0.010 | 0.902 | 0.005 |
| 659 | 11(A4e) | Tall | Vanuatu Tall | VUT | 0.991 | 0.009 | 0.030 | 0.012 | 0.044 | 0.908 | 0.006 |
| 660 | 11(A4e) | Tall | Vanuatu Tall | VUT | 0.995 | 0.005 | 0.876 | 0.018 | 0.023 | 0.079 | 0.004 |
| 661 | 11(A4e) | Tall | Vanuatu Tall | VUT | 0.995 | 0.005 | 0.717 | 0.097 | 0.046 | 0.135 | 0.004 |
| 662 | 11(A4e) | Tall | Vanuatu Tall | VUT | 0.994 | 0.006 | 0.848 | 0.014 | 0.015 | 0.119 | 0.005 |
| 663 | 11(A4e) | Tall | Vanuatu Tall | VUT | 0.985 | 0.015 | 0.496 | 0.013 | 0.083 | 0.398 | 0.010 |
| 664 | 11(A4e) | Tall | Vanuatu Tall | VUT | 0.995 | 0.005 | 0.908 | 0.014 | 0.019 | 0.055 | 0.004 |
| 665 | 11(A4e) | Tall | Vanuatu Tall | VUT | 0.994 | 0.006 | 0.923 | 0.011 | 0.012 | 0.049 | 0.004 |
| 666 | 11(A4e) | Tall | Vanuatu Tall | VUT | 0.994 | 0.006 | 0.483 | 0.013 | 0.193 | 0.306 | 0.005 |
| 667 | 11(A4e) | Tall | Vanuatu Tall | VUT | 0.965 | 0.035 | 0.567 | 0.022 | 0.062 | 0.319 | 0.030 |
| 668 | 11(A4e) | Tall | Vanuatu Tall | VUT | 0.995 | 0.005 | 0.933 | 0.013 | 0.013 | 0.038 | 0.004 |
| 669 | 11(A4e) | Tall | Vanuatu Tall | VUT | 0.992 | 0.008 | 0.816 | 0.082 | 0.069 | 0.027 | 0.006 |
| 670 | 11(A4e) | Tall | Vanuatu Tall | VUT | 0.993 | 0.007 | 0.565 | 0.029 | 0.026 | 0.375 | 0.005 |
| 671 | 11(A4e) | Tall | Vanuatu Tall | VUT | 0.991 | 0.009 | 0.960 | 0.006 | 0.007 | 0.023 | 0.005 |
| 672 | 11(A4e) | Tall | Vanuatu Tall | VUT | 0.986 | 0.014 | 0.073 | 0.126 | 0.736 | 0.047 | 0.018 |
| 673 | 11(A4e) | Tall | Vanuatu Tall | VUT | 0.990 | 0.010 | 0.869 | 0.011 | 0.024 | 0.089 | 0.007 |
| 674 | 11(A4e) | Tall | Vanuatu Tall | VUT | 0.995 | 0.005 | 0.888 | 0.013 | 0.031 | 0.064 | 0.004 |
| 675 | 11(A4e) | Tall | Vanuatu Tall | VUT | 0.994 | 0.006 | 0.357 | 0.154 | 0.067 | 0.416 | 0.005 |
| 676 | 11(A4e) | Tall | Vanuatu Tall | VUT | 0.927 | 0.073 | 0.937 | 0.008 | 0.018 | 0.016 | 0.021 |
| 677 | 11(A4e) | Tall | Vanuatu Tall | VUT | 0.993 | 0.007 | 0.936 | 0.008 | 0.010 | 0.041 | 0.005 |
| 678 | 11(A4e) | Tall | Vanuatu Tall | VUT | 0.995 | 0.005 | 0.908 | 0.008 | 0.036 | 0.044 | 0.004 |
| 679 | 11(A4e) | Tall | Vanuatu Tall | VUT | 0.995 | 0.005 | 0.587 | 0.044 | 0.038 | 0.327 | 0.004 |
| 680 | 11(A4e) | Tall | Vanuatu Tall | VUT | 0.995 | 0.005 | 0.834 | 0.007 | 0.031 | 0.125 | 0.004 |
| 681 | 11(A4e) | Tall | Vanuatu Tall | VUT | 0.996 | 0.004 | 0.967 | 0.007 | 0.012 | 0.011 | 0.004 |
| 682 | 11(A4e) | Tall | Vanuatu Tall | VUT | 0.994 | 0.006 | 0.961 | 0.008 | 0.008 | 0.019 | 0.004 |
| 683 | 11(A4e) | Tall | Vanuatu Tall | VUT | 0.965 | 0.035 | 0.672 | 0.021 | 0.211 | 0.076 | 0.020 |
| 684 | 11(A4e) | Tall | Vanuatu Tall | VUT | 0.992 | 0.008 | 0.789 | 0.023 | 0.148 | 0.035 | 0.005 |
| 685 | 11(A4e) | Tall | Vanuatu Tall | VUT | 0.926 | 0.074 | 0.869 | 0.012 | 0.022 | 0.070 | 0.027 |
| 686 | 11(A4e) | Tall | Vanuatu Tall | VUT | 0.976 | 0.024 | 0.936 | 0.008 | 0.026 | 0.019 | 0.011 |
| 687 | 11(A4e) | Tall | Vanuatu Tall | VUT | 0.973 | 0.027 | 0.919 | 0.027 | 0.013 | 0.027 | 0.015 |
| 688 | 11(A4e) | Tall | Vanuatu Tall | VUT | 0.987 | 0.013 | 0.954 | 0.007 | 0.011 | 0.022 | 0.007 |
| 689 | 11(A4e) | Tall | Vanuatu Tall | VUT | 0.891 | 0.109 | 0.928 | 0.006 | 0.016 | 0.019 | 0.032 |
| 690 | 11(A4e) | Tall | Vanuatu Tall | VUT | 0.976 | 0.024 | 0.924 | 0.018 | 0.015 | 0.032 | 0.011 |
| 691 | 11(A4e) | Tall | Vanuatu Tall | VUT | 0.956 | 0.044 | 0.945 | 0.010 | 0.006 | 0.019 | 0.020 |
| 692 | 11(A4e) | Tall | Vanuatu Tall | VUT | 0.971 | 0.029 | 0.892 | 0.007 | 0.028 | 0.062 | 0.011 |
| 693 | 11(A4e) | Tall | Vanuatu Tall | VUT | 0.977 | 0.023 | 0.940 | 0.022 | 0.011 | 0.016 | 0.011 |
| 694 | 11(A4e) | Tall | Vanuatu Tall | VUT | 0.995 | 0.005 | 0.963 | 0.008 | 0.013 | 0.013 | 0.004 |
| 695 | 11(A4e) | Tall | Vanuatu Tall | VUT | 0.995 | 0.005 | 0.952 | 0.015 | 0.008 | 0.021 | 0.004 |
| 696 | 11(A4e) | Tall | Vanuatu Tall | VUT | 0.989 | 0.011 | 0.727 | 0.010 | 0.229 | 0.029 | 0.006 |
| 697 | 11(A4e) | Tall | Vanuatu Tall | VUT | 0.993 | 0.007 | 0.895 | 0.014 | 0.063 | 0.022 | 0.006 |
| 698 | 11(A4e) | Tall | Vanuatu Tall | VUT | 0.864 | 0.136 | 0.926 | 0.009 | 0.010 | 0.013 | 0.042 |
| 699 | 11(A4e) | Tall | Vanuatu Tall | VUT | 0.925 | 0.075 | 0.909 | 0.007 | 0.007 | 0.013 | 0.064 |
| 700 | 11(A4e) | Tall | Vanuatu Tall | VUT | 0.980 | 0.020 | 0.944 | 0.013 | 0.017 | 0.016 | 0.009 |
| 701 | 11(A4e) | Tall | Vanuatu Tall | VUT | 0.978 | 0.022 | 0.948 | 0.007 | 0.011 | 0.024 | 0.010 |
| 702 | 11(A4e) | Tall | Vanuatu Tall | VUT | 0.995 | 0.005 | 0.961 | 0.009 | 0.013 | 0.014 | 0.003 |
| 703 | 11(A4e) | Tall | Vanuatu Tall | VUT | 0.992 | 0.008 | 0.801 | 0.078 | 0.035 | 0.081 | 0.006 |
| 704 | 11(A4e) | Tall | Vanuatu Tall | VUT | 0.991 | 0.009 | 0.758 | 0.031 | 0.023 | 0.180 | 0.007 |
| 705 | 11(A4e) | Tall | Vanuatu Tall | VUT | 0.992 | 0.008 | 0.963 | 0.011 | 0.011 | 0.011 | 0.004 |
| 706 | 11(A4e) | Tall | Vanuatu Tall | VUT | 0.994 | 0.006 | 0.948 | 0.022 | 0.010 | 0.016 | 0.005 |
| 707 | 11(A4e) | Tall | Vanuatu Tall | VUT | 0.913 | 0.087 | 0.939 | 0.010 | 0.017 | 0.018 | 0.016 |
| 708 | 11(A4e) | Tall | Vanuatu Tall | VUT | 0.899 | 0.101 | 0.935 | 0.006 | 0.009 | 0.023 | 0.027 |
| 709 | 11(A4e) | Tall | Vanuatu Tall | VUT | 0.987 | 0.013 | 0.953 | 0.006 | 0.009 | 0.025 | 0.007 |
| 710 | 11(A4e) | Tall | Vanuatu Tall | VUT | 0.989 | 0.011 | 0.933 | 0.024 | 0.018 | 0.019 | 0.006 |
| 711 | 11(A4e) | Tall | Vanuatu Tall | VUT | 0.988 | 0.012 | 0.960 | 0.009 | 0.010 | 0.016 | 0.006 |
| 712 | 11(A4e) | Tall | Vanuatu Tall | VUT | 0.989 | 0.011 | 0.958 | 0.006 | 0.015 | 0.014 | 0.007 |
| 713 | 11(A4e) | Tall | Vanuatu Tall | VUT | 0.973 | 0.027 | 0.936 | 0.010 | 0.020 | 0.022 | 0.012 |
| 714 | 11(A4e) | Tall | Vanuatu Tall | VUT | 0.993 | 0.007 | 0.948 | 0.008 | 0.012 | 0.027 | 0.005 |
| 715 | 11(A4e) | Tall | Vanuatu Tall | VUT | 0.995 | 0.005 | 0.946 | 0.018 | 0.016 | 0.017 | 0.004 |
| 716 | 11(A4e) | Tall | Vanuatu Tall | VUT | 0.993 | 0.007 | 0.904 | 0.067 | 0.010 | 0.014 | 0.005 |
| 717 | 11(A4e) | Tall | Vanuatu Tall | VUT | 0.995 | 0.005 | 0.959 | 0.013 | 0.009 | 0.016 | 0.004 |
| 718 | 11(A4e) | Tall | Vanuatu Tall | VUT | 0.993 | 0.007 | 0.947 | 0.009 | 0.010 | 0.029 | 0.005 |
| 719 | 11(A4e) | Tall | Vanuatu Tall | VUT | 0.995 | 0.005 | 0.946 | 0.015 | 0.020 | 0.016 | 0.004 |
| 720 | 11(A4e) | Tall | Vanuatu Tall | VUT | 0.990 | 0.010 | 0.968 | 0.007 | 0.007 | 0.012 | 0.005 |
| 721 | 11(A4e) | Tall | Vanuatu Tall | VUT | 0.953 | 0.047 | 0.939 | 0.006 | 0.011 | 0.027 | 0.016 |
| 722 | 11(A4e) | Tall | Vanuatu Tall | VUT | 0.989 | 0.011 | 0.943 | 0.016 | 0.012 | 0.023 | 0.006 |
| 723 | 11(A4e) | Tall | Vanuatu Tall | VUT | 0.984 | 0.016 | 0.966 | 0.005 | 0.008 | 0.015 | 0.006 |
| 724 | 11(A4e) | Tall | Vanuatu Tall | VUT | 0.975 | 0.025 | 0.957 | 0.007 | 0.009 | 0.019 | 0.008 |
| 725 | 11(A4e) | Tall | Vanuatu Tall | VUT | 0.979 | 0.021 | 0.835 | 0.013 | 0.119 | 0.024 | 0.008 |
| 726 | 11(A4e) | Tall | Vanuatu Tall | VUT | 0.992 | 0.008 | 0.953 | 0.006 | 0.017 | 0.018 | 0.006 |
| 727 | 11(A4e) | Tall | Vanuatu Tall | VUT | 0.992 | 0.008 | 0.957 | 0.012 | 0.010 | 0.014 | 0.006 |
| 728 | 11(A4e) | Tall | Vanuatu Tall | VUT | 0.993 | 0.007 | 0.947 | 0.010 | 0.009 | 0.028 | 0.005 |
| 729 | 11(A4e) | Tall | Vanuatu Tall | VUT | 0.893 | 0.107 | 0.951 | 0.008 | 0.006 | 0.010 | 0.025 |
| 730 | 11(A4e) | Tall | Vanuatu Tall | VUT | 0.838 | 0.162 | 0.921 | 0.005 | 0.010 | 0.016 | 0.047 |
| 731 | 11(A4e) | Tall | Vanuatu Tall | VUT | 0.956 | 0.044 | 0.955 | 0.011 | 0.008 | 0.014 | 0.012 |
| 732 | 11(A4e) | Tall | Vanuatu Tall | VUT | 0.889 | 0.111 | 0.909 | 0.009 | 0.011 | 0.034 | 0.037 |
| 733 | 11(A4e) | Tall | Vanuatu Tall | VUT | 0.990 | 0.010 | 0.947 | 0.007 | 0.009 | 0.031 | 0.005 |
| 734 | 11(A4e) | Tall | Vanuatu Tall | VUT | 0.959 | 0.041 | 0.956 | 0.006 | 0.008 | 0.019 | 0.011 |
| 735 | 11(A4e) | Tall | Vanuatu Tall | VUT | 0.995 | 0.005 | 0.971 | 0.008 | 0.006 | 0.011 | 0.004 |
| 736 | 12(A5) | Tall | Tuvalu Tall | TUV | 0.884 | 0.116 | 0.029 | 0.008 | 0.032 | 0.903 | 0.028 |
| 737 | 12(A5) | Tall | Tuvalu Tall | TUV | 0.993 | 0.007 | 0.019 | 0.008 | 0.019 | 0.950 | 0.004 |
| 738 | 12(A5) | Tall | Tuvalu Tall | TUV | 0.896 | 0.104 | 0.014 | 0.021 | 0.059 | 0.873 | 0.033 |
| 739 | 12(A5) | Tall | Tuvalu Tall | TUV | 0.700 | 0.300 | 0.020 | 0.005 | 0.037 | 0.714 | 0.224 |
| 740 | 12(A5) | Tall | Tuvalu Tall | TUV | 0.732 | 0.268 | 0.010 | 0.005 | 0.017 | 0.890 | 0.078 |
| 741 | 12(A5) | Tall | Tuvalu Tall | TUV | 0.968 | 0.032 | 0.017 | 0.005 | 0.011 | 0.959 | 0.007 |
| 742 | 12(A5) | Tall | Tuvalu Tall | TUV | 0.984 | 0.016 | 0.436 | 0.009 | 0.102 | 0.442 | 0.011 |
| 743 | 12(A5) | Tall | Tuvalu Tall | TUV | 0.849 | 0.151 | 0.035 | 0.007 | 0.012 | 0.907 | 0.039 |
| 744 | 12(A5) | Tall | Tuvalu Tall | TUV | 0.969 | 0.031 | 0.024 | 0.008 | 0.029 | 0.932 | 0.007 |
| 745 | 12(A5) | Tall | Tuvalu Tall | TUV | 0.968 | 0.032 | 0.426 | 0.011 | 0.028 | 0.507 | 0.028 |
| 746 | 12(A5) | Tall | Tuvalu Tall | TUV | 0.864 | 0.136 | 0.020 | 0.009 | 0.013 | 0.920 | 0.038 |
| 747 | 12(A5) | Tall | Tuvalu Tall | TUV | 0.968 | 0.032 | 0.010 | 0.008 | 0.328 | 0.644 | 0.010 |
| 748 | 12(A5) | Tall | Tuvalu Tall | TUV | 0.951 | 0.049 | 0.046 | 0.009 | 0.016 | 0.916 | 0.013 |
| 749 | 12(A5) | Tall | Tuvalu Tall | TUV | 0.991 | 0.009 | 0.021 | 0.006 | 0.016 | 0.951 | 0.006 |
| 750 | 12(A5) | Tall | Tuvalu Tall | TUV | 0.994 | 0.006 | 0.042 | 0.023 | 0.061 | 0.871 | 0.004 |
| 751 | 12(A5) | Tall | Tuvalu Tall | TUV | 0.993 | 0.007 | 0.022 | 0.012 | 0.031 | 0.930 | 0.004 |
| 752 | 12(A5) | Tall | Tuvalu Tall | TUV | 0.836 | 0.164 | 0.059 | 0.008 | 0.009 | 0.873 | 0.051 |
| 753 | 12(A5) | Tall | Kiribati Tall | KIR | 0.993 | 0.007 | 0.031 | 0.006 | 0.030 | 0.928 | 0.005 |
| 754 | 12(A5) | Tall | Kiribati Tall | KIR | 0.994 | 0.006 | 0.818 | 0.038 | 0.042 | 0.098 | 0.004 |
| 755 | 12(A5) | Tall | Kiribati Tall | KIR | 0.988 | 0.012 | 0.095 | 0.012 | 0.244 | 0.637 | 0.013 |
| 756 | 12(A5) | Tall | Kiribati Tall | KIR | 0.827 | 0.173 | 0.018 | 0.009 | 0.007 | 0.831 | 0.135 |
| 757 | 12(A5) | Tall | Kiribati Tall | KIR | 0.988 | 0.012 | 0.043 | 0.017 | 0.023 | 0.910 | 0.008 |
| 758 | 12(A5) | Tall | Marshall Island Tall | MHL | 0.803 | 0.197 | 0.019 | 0.008 | 0.083 | 0.704 | 0.187 |
| 759 | 12(A5) | Tall | Marshall Island Tall | MHL | 0.700 | 0.300 | 0.062 | 0.031 | 0.008 | 0.640 | 0.259 |
| 760 | 12(A5) | Tall | Marshall Island Tall | MHL | 0.766 | 0.234 | 0.073 | 0.007 | 0.014 | 0.712 | 0.194 |
| 761 | 12(A5) | Tall | Marshall Island Tall | MHL | 0.724 | 0.276 | 0.014 | 0.006 | 0.053 | 0.681 | 0.245 |
| 762 | 12(A5) | Tall | Marshall Island Tall | MHL | 0.981 | 0.019 | 0.011 | 0.009 | 0.011 | 0.960 | 0.009 |
| 763 | 12(A5) | Tall | Kiribati Tall | VUT | 0.611 | 0.389 | 0.099 | 0.072 | 0.022 | 0.451 | 0.356 |
| 764 | 12(A5) | Tall | Kiribati Tall | VUT | 0.459 | 0.541 | 0.016 | 0.056 | 0.022 | 0.441 | 0.465 |
| 765 | 12(A5) | Tall | Kiribati Tall | VUT | 0.900 | 0.100 | 0.048 | 0.007 | 0.013 | 0.904 | 0.028 |
| 766 | 12(A5) | Tall | Kiribati Tall | VUT | 0.966 | 0.034 | 0.048 | 0.135 | 0.018 | 0.789 | 0.010 |
| 767 | 12(A5) | Tall | Kiribati Tall | VUT | 0.893 | 0.107 | 0.593 | 0.043 | 0.031 | 0.250 | 0.085 |
| 768 | 12(A5) | Tall | Kiribati Tall | KIR | 0.832 | 0.168 | 0.140 | 0.047 | 0.026 | 0.642 | 0.145 |
| 769 | 12(A5) | Tall | Kiribati Tall | KIR | 0.813 | 0.187 | 0.492 | 0.009 | 0.090 | 0.246 | 0.162 |
| 770 | 12(A5) | Tall | Kiribati Tall | KIR | 0.803 | 0.197 | 0.041 | 0.040 | 0.028 | 0.756 | 0.135 |
| 771 | 12(A5) | Tall | Kiribati Tall | KIR | 0.986 | 0.014 | 0.171 | 0.011 | 0.025 | 0.784 | 0.008 |
| 772 | 12(A5) | Tall | Kiribati Tall | KIR | 0.778 | 0.222 | 0.141 | 0.012 | 0.016 | 0.643 | 0.189 |
| 773 | 12(A5) | Tall | Kiribati Tall | KIR | 0.793 | 0.207 | 0.043 | 0.009 | 0.028 | 0.767 | 0.154 |
| 774 | 12(A5) | Tall | Kiribati Tall | KIR | 0.949 | 0.051 | 0.010 | 0.006 | 0.014 | 0.960 | 0.010 |
| 775 | 12(A5) | Tall | Kiribati Tall | KIR | 0.947 | 0.053 | 0.022 | 0.010 | 0.006 | 0.945 | 0.018 |
| 776 | 12(A5) | Tall | Kiribati Tall | KIR | 0.972 | 0.028 | 0.011 | 0.008 | 0.008 | 0.962 | 0.010 |
| 777 | 12(A5) | Tall | Kiribati Tall | KIR | 0.944 | 0.056 | 0.020 | 0.008 | 0.031 | 0.926 | 0.014 |
| 778 | 12(A5) | Tall | Kiribati Tall | KIR | 0.945 | 0.055 | 0.162 | 0.006 | 0.008 | 0.804 | 0.020 |
| 779 | 13(A6) | Tall | Niu Leka Dwarf | FJI | 0.841 | 0.159 | 0.921 | 0.007 | 0.005 | 0.013 | 0.053 |
| 780 | 13(A6) | Tall | Niu Leka Dwarf | FJI | 0.758 | 0.242 | 0.847 | 0.010 | 0.010 | 0.010 | 0.124 |
| 781 | 13(A6) | Tall | Tahiti Tall | PYF | 0.983 | 0.017 | 0.913 | 0.025 | 0.014 | 0.037 | 0.011 |
| 782 | 13(A6) | Tall | Tahiti Tall | PYF | 0.841 | 0.159 | 0.736 | 0.006 | 0.013 | 0.145 | 0.100 |
| 783 | 13(A6) | Tall | Tahiti Tall | PYF | 0.994 | 0.006 | 0.278 | 0.012 | 0.033 | 0.672 | 0.004 |
| 784 | 13(A6) | Tall | Tahiti Tall | PYF | 0.993 | 0.007 | 0.726 | 0.009 | 0.176 | 0.084 | 0.005 |
| 785 | 13(A6) | Tall | Rotuman Tall | PYF | 0.995 | 0.005 | 0.919 | 0.007 | 0.035 | 0.035 | 0.004 |
| 786 | 13(A6) | Tall | Rotuman Tall | PYF | 0.995 | 0.005 | 0.817 | 0.012 | 0.054 | 0.114 | 0.004 |
| 787 | 13(A6) | Tall | Rotuman Tall | PYF | 0.994 | 0.006 | 0.133 | 0.012 | 0.017 | 0.833 | 0.004 |
| 788 | 13(A6) | Tall | Rotuman Tall | PYF | 0.988 | 0.012 | 0.249 | 0.012 | 0.016 | 0.715 | 0.008 |
| 789 | 13(A6) | Tall | Rotuman Tall | PYF | 0.993 | 0.007 | 0.127 | 0.006 | 0.017 | 0.846 | 0.005 |
| 790 | 13(A6) | Tall | Tonga Tall | TON | 0.977 | 0.023 | 0.963 | 0.007 | 0.007 | 0.013 | 0.010 |
| 791 | 13(A6) | Tall | Tonga Tall | TON | 0.993 | 0.007 | 0.910 | 0.008 | 0.017 | 0.060 | 0.005 |
| 792 | 13(A6) | Tall | Tonga Tall | TON | 0.992 | 0.008 | 0.830 | 0.007 | 0.008 | 0.149 | 0.006 |
| 793 | 13(A6) | Tall | Tonga Tall | TON | 0.948 | 0.052 | 0.926 | 0.005 | 0.007 | 0.045 | 0.016 |
| 794 | 13(A6) | Tall | Tonga Tall | TON | 0.987 | 0.013 | 0.762 | 0.010 | 0.032 | 0.187 | 0.008 |
| 795 | 13(A6) | ‘Compact’ Dwarf | Niu Leka Dwarf | FJI | 0.892 | 0.108 | 0.935 | 0.010 | 0.008 | 0.016 | 0.031 |
| 796 | 13(A6) | ‘Compact’ Dwarf | Niu Leka Dwarf | FJI | 0.966 | 0.034 | 0.963 | 0.007 | 0.006 | 0.014 | 0.011 |
| 797 | 13(A6) | ‘Compact’ Dwarf | Niu Leka Dwarf | FJI | 0.921 | 0.079 | 0.952 | 0.010 | 0.007 | 0.009 | 0.021 |
| 798 | 13(A6) | ‘Compact’ Dwarf | Niu Leka Dwarf | FJI | 0.758 | 0.242 | 0.809 | 0.008 | 0.014 | 0.044 | 0.125 |
| 799 | 13(A6) | ‘Compact’ Dwarf | Niu Leka Dwarf | FJI | 0.965 | 0.035 | 0.958 | 0.007 | 0.007 | 0.016 | 0.012 |
| 800 | 13(A6) | Tall | Niu Ni Magimagi | FJI | 0.983 | 0.017 | 0.877 | 0.032 | 0.028 | 0.052 | 0.011 |
| 801 | 13(A6) | Tall | Niu Ni Magimagi | FJI | 0.968 | 0.032 | 0.496 | 0.011 | 0.011 | 0.469 | 0.014 |
| 802 | 13(A6) | Tall | Niu Ni Magimagi | FJI | 0.861 | 0.139 | 0.492 | 0.024 | 0.014 | 0.401 | 0.070 |
| 803 | 13(A6) | Tall | Niu Ni Magimagi | FJI | 0.989 | 0.011 | 0.963 | 0.007 | 0.011 | 0.013 | 0.008 |
| 804 | 13(A6) | Tall | Cook Islands Tall | COK | 0.985 | 0.015 | 0.478 | 0.007 | 0.074 | 0.433 | 0.008 |
| 805 | 13(A6) | Tall | Cook Islands Tall | COK | 0.993 | 0.007 | 0.446 | 0.007 | 0.009 | 0.532 | 0.005 |
| 806 | 13(A6) | Tall | Cook Islands Tall | COK | 0.993 | 0.007 | 0.950 | 0.010 | 0.009 | 0.025 | 0.006 |
| 807 | 13(A6) | Tall | Cook Islands Tall | COK | 0.791 | 0.209 | 0.765 | 0.010 | 0.008 | 0.030 | 0.187 |
| 808 | 13(A6) | Tall | Cook Islands Tall | COK | 0.990 | 0.010 | 0.944 | 0.005 | 0.012 | 0.032 | 0.007 |
| 809 | 14(A7) | Tall | Panama Tall Aguadulce | PAN | 0.824 | 0.176 | 0.018 | 0.868 | 0.008 | 0.015 | 0.090 |
| 810 | 14(A7) | Tall | Panama Tall Aguadulce | PAN | 0.445 | 0.555 | 0.007 | 0.537 | 0.007 | 0.009 | 0.440 |
| 811 | 14(A7) | Tall | Panama Tall Aguadulce | PAN | 0.991 | 0.009 | 0.008 | 0.967 | 0.008 | 0.010 | 0.007 |
| 812 | 14(A7) | Tall | Panama Tall Aguadulce | PAN | 0.800 | 0.200 | 0.014 | 0.841 | 0.012 | 0.017 | 0.117 |
| 813 | 14(A7) | Tall | Panama Tall Aguadulce | PAN | 0.752 | 0.248 | 0.008 | 0.814 | 0.008 | 0.009 | 0.161 |
| 814 | 14(A7) | Tall | Panama Tall Aguadulce | PAN | 0.910 | 0.090 | 0.008 | 0.914 | 0.009 | 0.007 | 0.062 |
| 815 | 14(A7) | Tall | Panama Tall Monagre | PAN | 0.995 | 0.005 | 0.008 | 0.969 | 0.011 | 0.008 | 0.004 |
| 816 | 14(A7) | Tall | Panama Tall Monagre | PAN | 0.995 | 0.005 | 0.007 | 0.966 | 0.016 | 0.007 | 0.004 |
| 817 | 14(A7) | Tall | Panama Tall Monagre | PAN | 0.995 | 0.005 | 0.007 | 0.973 | 0.010 | 0.007 | 0.004 |
| 818 | 14(A7) | Tall | Panama Tall Monagre | PAN | 0.995 | 0.005 | 0.009 | 0.968 | 0.011 | 0.009 | 0.004 |
| 819 | 14(A7) | Tall | Panama Tall Monagre | PAN | 0.994 | 0.006 | 0.006 | 0.946 | 0.007 | 0.036 | 0.005 |
| 820 | 14(A7) | Tall | Panama Tall Monagre | PAN | 0.994 | 0.006 | 0.008 | 0.972 | 0.007 | 0.008 | 0.004 |
| 821 | 14(A7) | Tall | Panama Tall | PAN | 0.995 | 0.005 | 0.014 | 0.961 | 0.009 | 0.012 | 0.004 |
| 822 | 14(A7) | Tall | Panama Tall | PAN | 0.995 | 0.005 | 0.012 | 0.968 | 0.007 | 0.009 | 0.003 |
| 823 | 14(A7) | Tall | Panama Tall | PAN | 0.995 | 0.005 | 0.014 | 0.961 | 0.009 | 0.012 | 0.004 |
| 824 | 14(A7) | Tall | Panama Tall | PAN | 0.995 | 0.005 | 0.018 | 0.958 | 0.009 | 0.011 | 0.003 |
| 825 | 14(A7) | Tall | Panama Tall | PAN | 0.995 | 0.005 | 0.017 | 0.958 | 0.009 | 0.012 | 0.004 |
| 826 | 14(A7) | Tall | Panama Tall | PAN | 0.995 | 0.005 | 0.013 | 0.967 | 0.008 | 0.009 | 0.003 |
| 827 | 14(A7) | Tall | Panama Tall | PAN | 0.995 | 0.005 | 0.007 | 0.973 | 0.009 | 0.007 | 0.003 |
| 828 | 14(A7) | Tall | Panama Tall | PAN | 0.995 | 0.005 | 0.006 | 0.977 | 0.008 | 0.006 | 0.004 |
| 829 | 14(A7) | Tall | Panama Tall | PAN | 0.995 | 0.005 | 0.007 | 0.973 | 0.009 | 0.007 | 0.004 |
| 830 | 14(A7) | Tall | Panama Tall | PAN | 0.995 | 0.005 | 0.006 | 0.973 | 0.010 | 0.007 | 0.004 |
| 831 | 14(A7) | Tall | Panama Tall Aguadulce | PAN | 0.986 | 0.014 | 0.045 | 0.908 | 0.009 | 0.012 | 0.026 |
| 832 | 14(A7) | Tall | Panama Tall Aguadulce | PAN | 0.828 | 0.172 | 0.008 | 0.879 | 0.013 | 0.010 | 0.091 |
| 833 | 14(A7) | Tall | Panama Tall Aguadulce | PAN | 0.991 | 0.009 | 0.014 | 0.953 | 0.009 | 0.016 | 0.008 |
| 834 | 14(A7) | Tall | Panama Tall Aguadulce | PAN | 0.852 | 0.148 | 0.011 | 0.928 | 0.010 | 0.014 | 0.037 |
| 835 | 14(A7) | Tall | Panama Tall Monagre | PAN | 0.995 | 0.005 | 0.007 | 0.974 | 0.009 | 0.007 | 0.004 |
| 836 | 14(A7) | Tall | Panama Tall Monagre | PAN | 0.995 | 0.005 | 0.011 | 0.943 | 0.033 | 0.009 | 0.004 |
| 837 | 14(A7) | Tall | Panama Tall Monagre | PAN | 0.995 | 0.005 | 0.045 | 0.880 | 0.012 | 0.059 | 0.003 |
| 838 | 14(A7) | Tall | Panama Tall Monagre | PAN | 0.995 | 0.005 | 0.022 | 0.852 | 0.103 | 0.018 | 0.005 |
| 839 | 14(A7) | Tall | Panama Tall Monagre | PAN | 0.994 | 0.006 | 0.018 | 0.914 | 0.043 | 0.021 | 0.004 |
| 840 | 14(A7) | Tall | Panama Tall Monagre | PAN | 0.995 | 0.005 | 0.006 | 0.971 | 0.013 | 0.006 | 0.004 |
| 841 | 14(A7) | Tall | Panama Tall Monagre | PAN | 0.995 | 0.005 | 0.007 | 0.973 | 0.009 | 0.007 | 0.004 |
| 842 | 14(A7) | Tall | Panama Tall Monagre | PAN | 0.995 | 0.005 | 0.007 | 0.971 | 0.010 | 0.008 | 0.004 |
| 843 | 14(A7) | Tall | Panama Tall Monagre | PAN | 0.995 | 0.005 | 0.009 | 0.967 | 0.012 | 0.008 | 0.004 |
| 844 | 14(A7) | Tall | Panama Tall Monagre | PAN | 0.995 | 0.005 | 0.007 | 0.973 | 0.010 | 0.007 | 0.004 |
| 845 | 14(A7) | Tall | Panama Tall Monagre | PAN | 0.775 | 0.225 | 0.047 | 0.725 | 0.008 | 0.023 | 0.197 |
| 846 | 14(A7) | Tall | Panama Tall Monagre | PAN | 0.995 | 0.005 | 0.006 | 0.977 | 0.008 | 0.006 | 0.003 |
| 847 | 14(A7) | Tall | Panama Tall Monagre | PAN | 0.995 | 0.005 | 0.006 | 0.978 | 0.007 | 0.006 | 0.004 |
| 848 | 14(A7) | Tall | Panama Tall | PAN | 0.681 | 0.319 | 0.007 | 0.725 | 0.008 | 0.007 | 0.253 |
| 849 | 14(A7) | Tall | Panama Tall | PAN | 0.993 | 0.007 | 0.026 | 0.893 | 0.023 | 0.043 | 0.015 |
| 850 | 14(A7) | Tall | Panama Tall | PAN | 0.867 | 0.133 | 0.009 | 0.828 | 0.008 | 0.013 | 0.142 |
| 851 | 14(A7) | Tall | Panama Tall | PAN | 0.988 | 0.012 | 0.015 | 0.928 | 0.008 | 0.016 | 0.034 |
| 852 | 14(A7) | Tall | Panama Tall | PAN | 0.993 | 0.007 | 0.021 | 0.905 | 0.022 | 0.037 | 0.016 |
| 853 | 14(A7) | Tall | Panama tall Aguadulce | PAN | 0.689 | 0.311 | 0.016 | 0.717 | 0.010 | 0.028 | 0.228 |
| 854 | 14(A7) | Tall | Panama tall Aguadulce | PAN | 0.806 | 0.194 | 0.019 | 0.844 | 0.010 | 0.015 | 0.112 |
| 855 | 14(A7) | Tall | Panama tall Aguadulce | PAN | 0.877 | 0.123 | 0.013 | 0.875 | 0.015 | 0.025 | 0.072 |
| 856 | 14(A7) | Tall | Panama Tall Cr | PAN | 0.994 | 0.006 | 0.009 | 0.966 | 0.007 | 0.012 | 0.006 |
| 857 | 14(A7) | Tall | Panama Tall Cr | PAN | 0.995 | 0.005 | 0.009 | 0.865 | 0.108 | 0.013 | 0.005 |
| 858 | 14(A7) | Tall | Panama Tall Cr | PAN | 0.995 | 0.005 | 0.005 | 0.980 | 0.007 | 0.005 | 0.003 |
| 859 | 14(A7) | Tall | Panama Tall Cr | PAN | 0.995 | 0.005 | 0.007 | 0.944 | 0.039 | 0.007 | 0.004 |
| 860 | 14(A7) | Tall | Panama Tall Cr | PAN | 0.992 | 0.008 | 0.040 | 0.326 | 0.474 | 0.153 | 0.007 |
| 861 | 14(A7) | Tall | Panama Tall Cr | PAN | 0.994 | 0.006 | 0.010 | 0.936 | 0.033 | 0.015 | 0.006 |
| 862 | 14(A7) | Tall | Panama Tall Cr | PAN | 0.995 | 0.005 | 0.005 | 0.977 | 0.007 | 0.007 | 0.003 |
| 863 | 14(A7) | Tall | Panama Tall Cr | PAN | 0.995 | 0.005 | 0.007 | 0.974 | 0.008 | 0.007 | 0.004 |
| 864 | 14(A7) | Tall | Panama Tall Cr | PAN | 0.994 | 0.006 | 0.010 | 0.936 | 0.032 | 0.015 | 0.006 |
| 865 | 14(A7) | Tall | Panama Tall Cr | PAN | 0.993 | 0.007 | 0.018 | 0.903 | 0.033 | 0.038 | 0.009 |
| 866 | 14(A7) | Tall | Panama Tall Cr | PAN | 0.993 | 0.007 | 0.015 | 0.887 | 0.043 | 0.043 | 0.012 |
| 867 | 14(A7) | Tall | Panama Tall Cr | PAN | 0.985 | 0.015 | 0.014 | 0.907 | 0.006 | 0.022 | 0.050 |
| 868 | 14(A7) | Tall | Panama Tall Cr | PAN | 0.995 | 0.005 | 0.007 | 0.966 | 0.018 | 0.006 | 0.003 |
| 869 | 14(A7) | Tall | Panama Tall Cr | PAN | 0.992 | 0.008 | 0.007 | 0.939 | 0.036 | 0.012 | 0.006 |
| 870 | 14(A7) | Tall | Panama Tall Cr | PAN | 0.995 | 0.005 | 0.005 | 0.981 | 0.006 | 0.005 | 0.003 |
| 871 | 14(A7) | Tall | Panama Tall Cr | PAN | 0.995 | 0.005 | 0.007 | 0.932 | 0.008 | 0.048 | 0.005 |
| 872 | 14(A7) | Tall | Panama Tall Cr | PAN | 0.995 | 0.005 | 0.006 | 0.969 | 0.015 | 0.006 | 0.003 |
| 873 | 14(A7) | Tall | Panama Tall Cr | PAN | 0.995 | 0.005 | 0.009 | 0.908 | 0.067 | 0.013 | 0.004 |
| 874 | 14(A7) | Tall | Panama Tall Cr | PAN | 0.995 | 0.005 | 0.004 | 0.982 | 0.005 | 0.005 | 0.004 |
| 875 | 14(A7) | Tall | Panama Tall | PAN | 0.748 | 0.252 | 0.007 | 0.811 | 0.010 | 0.008 | 0.163 |
| 876 | 14(A7) | Tall | Panama Tall | PAN | 0.961 | 0.039 | 0.014 | 0.910 | 0.007 | 0.007 | 0.062 |
| 877 | 14(A7) | Tall | Panama Tall | PAN | 0.981 | 0.019 | 0.047 | 0.833 | 0.009 | 0.093 | 0.018 |
| 878 | 14(A7) | Tall | Panama Tall | PAN | 0.639 | 0.361 | 0.006 | 0.726 | 0.007 | 0.008 | 0.253 |
| 879 | 14(A7) | Tall | Panama Tall | PAN | 0.667 | 0.333 | 0.009 | 0.723 | 0.009 | 0.010 | 0.249 |
| 880 | 14(A7) | Tall | Panama Tall | PAN | 0.972 | 0.028 | 0.019 | 0.914 | 0.008 | 0.011 | 0.048 |
| 881 | 14(A7) | Tall | Panama Tall | PAN | 0.995 | 0.005 | 0.006 | 0.972 | 0.010 | 0.008 | 0.004 |
| 882 | 14(A7) | Tall | Panama Tall | PAN | 0.991 | 0.009 | 0.013 | 0.939 | 0.013 | 0.027 | 0.007 |
| 883 | 14(A7) | Tall | Panama Tall | PAN | 0.799 | 0.201 | 0.008 | 0.864 | 0.008 | 0.009 | 0.111 |
| 884 | 14(A7) | Tall | Panama Tall | PAN | 0.852 | 0.148 | 0.008 | 0.926 | 0.007 | 0.010 | 0.049 |
| 885 | 14(A7) | Tall | Panama Tall | PAN | 0.991 | 0.009 | 0.020 | 0.923 | 0.014 | 0.036 | 0.007 |
| 886 | 14(A7) | Tall | Panama Tall | PAN | 0.991 | 0.009 | 0.010 | 0.964 | 0.009 | 0.012 | 0.006 |
| 887 | 14(A7) | Tall | Panama Tall | PAN | 0.943 | 0.057 | 0.066 | 0.178 | 0.036 | 0.699 | 0.021 |
| 888 | 14(A7) | Tall | Panama Tall | PAN | 0.995 | 0.005 | 0.007 | 0.974 | 0.009 | 0.007 | 0.004 |
| 889 | 14(A7) | Tall | Panama Tall | PAN | 0.995 | 0.005 | 0.006 | 0.978 | 0.007 | 0.006 | 0.004 |
| 890 | 14(A7) | Tall | Panama Tall | PAN | 0.995 | 0.005 | 0.006 | 0.977 | 0.008 | 0.006 | 0.003 |
| 891 | 14(A7) | Tall | Panama Tall | PAN | 0.995 | 0.005 | 0.008 | 0.969 | 0.011 | 0.008 | 0.004 |
| 892 | 14(A7) | Tall | Panama Tall | PAN | 0.995 | 0.005 | 0.008 | 0.970 | 0.009 | 0.009 | 0.004 |
| 893 | 14(A7) | Tall | Panama Tall | PAN | 0.995 | 0.005 | 0.007 | 0.975 | 0.008 | 0.007 | 0.004 |
| 894 | 14(A7) | Tall | Panama Tall | PAN | 0.995 | 0.005 | 0.009 | 0.966 | 0.012 | 0.009 | 0.004 |
| 895 | 14(A7) | Tall | Panama Tall | PAN | 0.995 | 0.005 | 0.006 | 0.976 | 0.009 | 0.006 | 0.003 |
| 896 | 14(A7) | Tall | Panama Tall | PAN | 0.995 | 0.005 | 0.005 | 0.978 | 0.007 | 0.006 | 0.004 |
| 897 | 14(A7) | Tall | Panama Tall | PAN | 0.667 | 0.333 | 0.059 | 0.086 | 0.527 | 0.031 | 0.298 |
| 898 | 14(A7) | Tall | Panama Tall | PAN | 0.994 | 0.006 | 0.006 | 0.977 | 0.007 | 0.007 | 0.004 |
| 899 | 14(A7) | Tall | Panama Tall | PAN | 0.995 | 0.005 | 0.006 | 0.977 | 0.008 | 0.006 | 0.004 |
| 900 | 14(A7) | Tall | Panama Tall | PAN | 0.995 | 0.005 | 0.010 | 0.966 | 0.012 | 0.009 | 0.004 |
| 901 | 14(A7) | Tall | Panama Tall | PAN | 0.995 | 0.005 | 0.005 | 0.977 | 0.009 | 0.006 | 0.003 |
| 902 | 14(A7) | Tall | Panama Tall | PAN | 0.995 | 0.005 | 0.005 | 0.978 | 0.007 | 0.006 | 0.004 |
| 903 | 14(A7) | Tall | Panama Tall | PAN | 0.995 | 0.005 | 0.006 | 0.977 | 0.008 | 0.005 | 0.003 |
| 904 | 14(A7) | Tall | Panama Tall | PAN | 0.994 | 0.006 | 0.007 | 0.975 | 0.007 | 0.007 | 0.004 |
| 905 | 14(A7) | Tall | Panama Tall | PAN | 0.995 | 0.005 | 0.005 | 0.978 | 0.007 | 0.006 | 0.004 |
| 906 | 14(A7) | Tall | Panama Tall | PAN | 0.995 | 0.005 | 0.006 | 0.975 | 0.009 | 0.007 | 0.004 |
| 907 | 14(A7) | Tall | Panama Tall | PAN | 0.933 | 0.067 | 0.006 | 0.925 | 0.008 | 0.006 | 0.055 |
| 908 | 14(A7) | Tall | Panama Tall | PAN | 0.995 | 0.005 | 0.012 | 0.966 | 0.009 | 0.009 | 0.004 |
| 909 | 14(A7) | Tall | Panama Tall | PAN | 0.995 | 0.005 | 0.012 | 0.960 | 0.011 | 0.014 | 0.004 |
| 910 | 14(A7) | Tall | Panama Tall | PAN | 0.995 | 0.005 | 0.005 | 0.977 | 0.009 | 0.006 | 0.004 |
| 911 | 14(A7) | Tall | Panama Tall | PAN | 0.995 | 0.005 | 0.008 | 0.969 | 0.009 | 0.010 | 0.003 |
| 912 | 14(A7) | Tall | Panama Tall | PAN | 0.995 | 0.005 | 0.009 | 0.967 | 0.012 | 0.009 | 0.004 |
| 913 | 14(A7) | Tall | Panama Tall | PAN | 0.995 | 0.005 | 0.006 | 0.976 | 0.008 | 0.007 | 0.004 |
| 914 | 15(B1) | Tall | West African Tall Côte d'Ivoire | CIV | 0.004 | 0.996 | 0.004 | 0.003 | 0.003 | 0.004 | 0.986 |
| 915 | 15(B1) | Tall | West African Tall Côte d'Ivoire | CIV | 0.004 | 0.996 | 0.004 | 0.003 | 0.003 | 0.004 | 0.986 |
| 916 | 15(B1) | Tall | West African Tall Côte d'Ivoire | CIV | 0.004 | 0.996 | 0.004 | 0.003 | 0.003 | 0.004 | 0.986 |
| 917 | 15(B1) | Tall | West African Tall Côte d'Ivoire | CIV | 0.004 | 0.996 | 0.004 | 0.003 | 0.003 | 0.004 | 0.986 |
| 918 | 15(B1) | Tall | West African Tall Côte d'Ivoire | CIV | 0.005 | 0.995 | 0.004 | 0.004 | 0.004 | 0.004 | 0.985 |
| 919 | 15(B1) | Tall | West African Tall Côte d'Ivoire | CIV | 0.004 | 0.996 | 0.004 | 0.003 | 0.003 | 0.004 | 0.986 |
| 920 | 15(B1) | Tall | West African Tall Côte d'Ivoire | CIV | 0.004 | 0.996 | 0.004 | 0.003 | 0.003 | 0.003 | 0.986 |
| 921 | 15(B1) | Tall | West African Tall Côte d'Ivoire | CIV | 0.004 | 0.996 | 0.004 | 0.003 | 0.003 | 0.003 | 0.986 |
| 922 | 15(B1) | Tall | West African Tall Côte d'Ivoire | CIV | 0.004 | 0.996 | 0.004 | 0.003 | 0.003 | 0.004 | 0.986 |
| 923 | 15(B1) | Tall | West African Tall Côte d'Ivoire | CIV | 0.004 | 0.996 | 0.003 | 0.004 | 0.003 | 0.004 | 0.986 |
| 924 | 15(B1) | Tall | West African Tall Benin | BEN | 0.005 | 0.995 | 0.004 | 0.005 | 0.005 | 0.005 | 0.981 |
| 925 | 15(B1) | Tall | West African Tall Benin | BEN | 0.004 | 0.996 | 0.004 | 0.003 | 0.003 | 0.004 | 0.986 |
| 926 | 15(B1) | Tall | West African Tall Benin | BEN | 0.007 | 0.993 | 0.005 | 0.009 | 0.008 | 0.006 | 0.971 |
| 927 | 15(B1) | Tall | West African Tall Benin | BEN | 0.006 | 0.994 | 0.004 | 0.005 | 0.008 | 0.006 | 0.977 |
| 928 | 15(B1) | Tall | West African Tall Benin | BEN | 0.005 | 0.995 | 0.004 | 0.005 | 0.005 | 0.005 | 0.981 |
| 929 | 15(B1) | Tall | West African Tall Benin | BEN | 0.005 | 0.995 | 0.005 | 0.003 | 0.006 | 0.005 | 0.982 |
| 930 | 15(B1) | Tall | West African Tall Benin | BEN | 0.005 | 0.995 | 0.005 | 0.004 | 0.004 | 0.005 | 0.982 |
| 931 | 15(B1) | Tall | West African Tall Benin | BEN | 0.008 | 0.992 | 0.011 | 0.016 | 0.024 | 0.012 | 0.937 |
| 932 | 15(B1) | Tall | West African Tall Benin | BEN | 0.005 | 0.995 | 0.004 | 0.003 | 0.006 | 0.005 | 0.982 |
| 933 | 15(B1) | Tall | West African Tall Benin | BEN | 0.005 | 0.995 | 0.004 | 0.009 | 0.009 | 0.005 | 0.974 |
| 934 | 15(B1) | Tall | West African Tall | CIV | 0.004 | 0.996 | 0.004 | 0.003 | 0.003 | 0.004 | 0.986 |
| 935 | 15(B1) | Tall | West African Tall | CIV | 0.004 | 0.996 | 0.004 | 0.003 | 0.003 | 0.004 | 0.986 |
| 936 | 15(B1) | Tall | West African Tall | CIV | 0.005 | 0.995 | 0.004 | 0.004 | 0.005 | 0.005 | 0.982 |
| 937 | 15(B1) | Tall | West African Tall | CIV | 0.004 | 0.996 | 0.004 | 0.003 | 0.003 | 0.003 | 0.986 |
| 938 | 15(B1) | Tall | Laccadive Micro Tall | IND | 0.005 | 0.995 | 0.004 | 0.005 | 0.005 | 0.005 | 0.981 |
| 939 | 15(B1) | Tall | Laccadive Micro Tall | IND | 0.004 | 0.996 | 0.004 | 0.003 | 0.003 | 0.004 | 0.985 |
| 940 | 15(B1) | Tall | Laccadive Micro Tall | IND | 0.004 | 0.996 | 0.004 | 0.003 | 0.003 | 0.004 | 0.986 |
| 941 | 15(B1) | Tall | Laccadive Micro Tall | IND | 0.013 | 0.987 | 0.014 | 0.014 | 0.010 | 0.018 | 0.943 |
| 942 | 15(B1) | Tall | Laccadive Micro Tall | IND | 0.004 | 0.996 | 0.004 | 0.004 | 0.003 | 0.004 | 0.986 |
| 943 | 15(B1) | Tall | Sri Lanka Tall | LKA | 0.016 | 0.984 | 0.024 | 0.005 | 0.008 | 0.022 | 0.941 |
| 944 | 15(B1) | Tall | Sri Lanka Tall | LKA | 0.005 | 0.995 | 0.004 | 0.003 | 0.005 | 0.004 | 0.983 |
| 945 | 15(B1) | Tall | Sri Lanka Tall | LKA | 0.006 | 0.994 | 0.006 | 0.007 | 0.006 | 0.007 | 0.974 |
| 946 | 15(B1) | Tall | Sri Lanka Tall | LKA | 0.012 | 0.988 | 0.018 | 0.006 | 0.006 | 0.018 | 0.952 |
| 947 | 15(B1) | Tall | Cameroon Kribi Tall | CMR | 0.005 | 0.995 | 0.005 | 0.006 | 0.004 | 0.004 | 0.981 |
| 948 | 15(B1) | Tall | Cameroon Kribi Tall | CMR | 0.013 | 0.987 | 0.016 | 0.011 | 0.011 | 0.016 | 0.947 |
| 949 | 15(B1) | Tall | Cameroon Kribi Tall | CMR | 0.013 | 0.987 | 0.024 | 0.018 | 0.031 | 0.015 | 0.911 |
| 950 | 15(B1) | Tall | Cameroon Kribi Tall | CMR | 0.008 | 0.992 | 0.015 | 0.018 | 0.015 | 0.010 | 0.943 |
| 951 | 15(B1) | Tall | Cameroon Kribi Tall | CMR | 0.008 | 0.992 | 0.010 | 0.012 | 0.007 | 0.007 | 0.964 |
| 952 | 15(B1) | Tall | Jamaica Tall | JAM | 0.006 | 0.994 | 0.006 | 0.004 | 0.005 | 0.007 | 0.978 |
| 953 | 15(B1) | Tall | Jamaica Tall | JAM | 0.007 | 0.993 | 0.008 | 0.008 | 0.006 | 0.007 | 0.972 |
| 954 | 15(B1) | Tall | Jamaica Tall | JAM | 0.014 | 0.986 | 0.010 | 0.029 | 0.019 | 0.011 | 0.930 |
| 955 | 15(B1) | Tall | Jamaica Tall | JAM | 0.005 | 0.995 | 0.004 | 0.005 | 0.005 | 0.005 | 0.981 |
| 956 | 15(B1) | Tall | Mexican Atlantic Tall | MEX | 0.027 | 0.973 | 0.035 | 0.012 | 0.041 | 0.054 | 0.858 |
| 957 | 15(B1) | Tall | Mexican Atlantic Tall | MEX | 0.019 | 0.981 | 0.021 | 0.071 | 0.021 | 0.017 | 0.870 |
| 958 | 15(B1) | Tall | Mexican Atlantic Tall | MEX | 0.011 | 0.989 | 0.018 | 0.014 | 0.045 | 0.013 | 0.910 |
| 959 | 15(B1) | Tall | Mexican Atlantic Tall | MEX | 0.014 | 0.986 | 0.013 | 0.008 | 0.042 | 0.034 | 0.904 |
| 960 | 15(B1) | Tall | Mexican Atlantic Tall | MEX | 0.007 | 0.993 | 0.007 | 0.004 | 0.005 | 0.007 | 0.978 |
| 961 | 15(B1) | Tall | Sri Lanka Tall | LKA | 0.005 | 0.995 | 0.004 | 0.004 | 0.006 | 0.004 | 0.982 |
| 962 | 15(B1) | Tall | Sri Lanka Tall | LKA | 0.006 | 0.994 | 0.005 | 0.006 | 0.007 | 0.006 | 0.976 |
| 963 | 15(B1) | Tall | Sri Lanka Tall | LKA | 0.008 | 0.992 | 0.007 | 0.009 | 0.009 | 0.008 | 0.966 |
| 964 | 15(B1) | Tall | Sri Lanka Tall | LKA | 0.004 | 0.996 | 0.004 | 0.003 | 0.003 | 0.004 | 0.986 |
| 965 | 15(B1) | Tall | Calangute Tall | IND | 0.387 | 0.613 | 0.055 | 0.010 | 0.023 | 0.403 | 0.510 |
| 966 | 15(B1) | Tall | Calangute Tall | IND | 0.004 | 0.996 | 0.004 | 0.004 | 0.003 | 0.004 | 0.986 |
| 967 | 15(B1) | Tall | Calangute Tall | IND | 0.005 | 0.995 | 0.004 | 0.004 | 0.004 | 0.004 | 0.984 |
| 968 | 15(B1) | Tall | Indian West Coast Tall | IND | 0.006 | 0.994 | 0.005 | 0.004 | 0.007 | 0.006 | 0.978 |
| 969 | 15(B1) | Tall | Indian West Coast Tall | IND | 0.012 | 0.988 | 0.019 | 0.007 | 0.009 | 0.009 | 0.956 |
| 970 | 15(B1) | Tall | Indian West Coast Tall | IND | 0.006 | 0.994 | 0.006 | 0.004 | 0.009 | 0.008 | 0.974 |
| 971 | 15(B1) | Tall | Indian West Coast Tall | IND | 0.005 | 0.995 | 0.004 | 0.004 | 0.004 | 0.005 | 0.984 |
| 972 | 15(B1) | Tall | Indian West Coast Tall | IND | 0.005 | 0.995 | 0.004 | 0.004 | 0.004 | 0.005 | 0.982 |
| 973 | 15(B1) | Tall | Indian West Coast Tall | IND | 0.010 | 0.990 | 0.015 | 0.016 | 0.008 | 0.019 | 0.942 |
| 974 | 15(B1) | Tall | Sakhi Ghopal Tall | IND | 0.005 | 0.995 | 0.004 | 0.003 | 0.005 | 0.004 | 0.983 |
| 975 | 15(B1) | Tall | Sakhi Ghopal Tall | IND | 0.005 | 0.995 | 0.005 | 0.003 | 0.005 | 0.004 | 0.982 |
| 976 | 15(B1) | Tall | Sakhi Ghopal Tall | IND | 0.006 | 0.994 | 0.005 | 0.004 | 0.006 | 0.005 | 0.980 |
| 977 | 15(B1) | Tall | Sakhi Ghopal Tall | IND | 0.006 | 0.994 | 0.005 | 0.004 | 0.007 | 0.007 | 0.977 |
| 978 | 15(B1) | Tall | Sakhi Ghopal Tall | IND | 0.008 | 0.992 | 0.015 | 0.010 | 0.023 | 0.011 | 0.942 |
| 979 | 15(B1) | Tall | Mexican Atlantic Tall | MEX | 0.011 | 0.989 | 0.019 | 0.014 | 0.045 | 0.014 | 0.909 |
| 980 | 15(B1) | Tall | Mexican Atlantic Tall | MEX | 0.006 | 0.994 | 0.005 | 0.005 | 0.005 | 0.006 | 0.980 |
| 981 | 15(B1) | Tall | Mexican Atlantic Tall | MEX | 0.155 | 0.845 | 0.025 | 0.020 | 0.193 | 0.087 | 0.675 |
| 982 | 15(B1) | Tall | Mexican Atlantic Tall | MEX | 0.009 | 0.991 | 0.013 | 0.006 | 0.017 | 0.018 | 0.946 |
| 983 | 15(B1) | Tall | Sri Lanka Tall | LKA | 0.079 | 0.921 | 0.058 | 0.005 | 0.010 | 0.092 | 0.834 |
| 984 | 15(B1) | Tall | Sri Lanka Tall | LKA | 0.045 | 0.955 | 0.019 | 0.022 | 0.104 | 0.053 | 0.802 |
| 985 | 15(B1) | Tall | Sri Lanka Tall | LKA | 0.008 | 0.992 | 0.006 | 0.019 | 0.014 | 0.008 | 0.954 |
| 986 | 15(B1) | Tall | Sri Lanka Tall | LKA | 0.005 | 0.995 | 0.004 | 0.004 | 0.004 | 0.004 | 0.984 |
| 987 | 15(B1) | Tall | Sri Lanka Tall | LKA | 0.035 | 0.965 | 0.046 | 0.004 | 0.055 | 0.067 | 0.828 |
| 988 | 15(B1) | Tall | Sri Lanka Tall | LKA | 0.017 | 0.983 | 0.025 | 0.004 | 0.074 | 0.027 | 0.870 |
| 989 | 15(B1) | Tall | Sri Lanka Tall | LKA | 0.006 | 0.994 | 0.004 | 0.006 | 0.010 | 0.005 | 0.975 |
| 990 | 15(B1) | Tall | Sri Lanka Tall | LKA | 0.011 | 0.989 | 0.010 | 0.011 | 0.016 | 0.011 | 0.952 |
| 991 | 15(B1) | Tall | Sri Lanka Tall | LKA | 0.008 | 0.992 | 0.006 | 0.016 | 0.008 | 0.007 | 0.964 |
| 992 | 15(B1) | Tall | Brazilian Tall | BRA | 0.004 | 0.996 | 0.004 | 0.003 | 0.003 | 0.004 | 0.986 |
| 993 | 15(B1) | Tall | Brazilian Tall | BRA | 0.946 | 0.054 | 0.019 | 0.048 | 0.362 | 0.529 | 0.042 |
| 994 | 15(B1) | Tall | Brazilian Tall | BRA | 0.038 | 0.962 | 0.057 | 0.013 | 0.132 | 0.014 | 0.784 |
| 995 | 15(B1) | Tall | Brazilian Tall | BRA | 0.013 | 0.987 | 0.019 | 0.018 | 0.011 | 0.015 | 0.937 |
| 996 | 15(B1) | Tall | Brazilian Tall Praia Do forte | BRA | 0.010 | 0.990 | 0.013 | 0.004 | 0.011 | 0.011 | 0.960 |
| 997 | 15(B1) | Tall | Brazilian Tall Praia Do forte | BRA | 0.008 | 0.992 | 0.010 | 0.005 | 0.008 | 0.009 | 0.968 |
| 998 | 15(B1) | Tall | Brazilian Tall Praia Do forte | BRA | 0.013 | 0.987 | 0.024 | 0.014 | 0.009 | 0.025 | 0.928 |
| 999 | 15(B1) | Tall | Brazilian Tall Praia Do forte | BRA | 0.012 | 0.988 | 0.018 | 0.008 | 0.013 | 0.011 | 0.949 |
| 1000 | 15(B1) | Tall | Brazilian Tall | BRA | 0.009 | 0.991 | 0.013 | 0.006 | 0.010 | 0.008 | 0.964 |
| 1001 | 15(B1) | Tall | Brazilian Tall | BRA | 0.013 | 0.987 | 0.020 | 0.029 | 0.009 | 0.019 | 0.923 |
| 1002 | 15(B1) | Tall | Brazilian Tall | BRA | 0.027 | 0.973 | 0.061 | 0.027 | 0.013 | 0.032 | 0.868 |
| 1003 | 15(B1) | Tall | Brazilian Tall | BRA | 0.009 | 0.991 | 0.013 | 0.005 | 0.007 | 0.010 | 0.964 |
| 1004 | 15(B1) | Tall | Brazilian Tall | BRA | 0.006 | 0.994 | 0.006 | 0.006 | 0.006 | 0.005 | 0.977 |
| 1005 | 15(B1) | Tall | Brazilian Tall | BRA | 0.005 | 0.995 | 0.005 | 0.006 | 0.004 | 0.004 | 0.981 |
| 1006 | 15(B1) | Tall | Brazilian Tall | BRA | 0.032 | 0.968 | 0.035 | 0.036 | 0.057 | 0.024 | 0.847 |
| 1007 | 15(B1) | Tall | Brazilian Tall | BRA | 0.004 | 0.996 | 0.004 | 0.003 | 0.003 | 0.004 | 0.986 |
| 1008 | 15(B1) | Tall | Brazilian Tall | BRA | 0.015 | 0.985 | 0.009 | 0.006 | 0.148 | 0.013 | 0.824 |
| 1009 | 15(B1) | Tall | Brazilian Tall | BRA | 0.007 | 0.993 | 0.007 | 0.004 | 0.012 | 0.008 | 0.970 |
| 1010 | 15(B1) | Tall | Brazilian Tall | BRA | 0.005 | 0.995 | 0.004 | 0.006 | 0.007 | 0.005 | 0.978 |
| 1011 | 15(B1) | Tall | Brazilian Tall | BRA | 0.012 | 0.988 | 0.015 | 0.005 | 0.022 | 0.015 | 0.944 |
| 1012 | 15(B1) | Tall | Brazilian Tall | BRA | 0.010 | 0.990 | 0.010 | 0.004 | 0.042 | 0.010 | 0.934 |
| 1013 | 15(B1) | Tall | Brazilian Tall | BRA | 0.008 | 0.992 | 0.005 | 0.011 | 0.044 | 0.008 | 0.933 |
| 1014 | 15(B1) | Tall | Brazilian Tall | BRA | 0.007 | 0.993 | 0.005 | 0.005 | 0.008 | 0.006 | 0.974 |
| 1015 | 15(B1) | Tall | Brazilian Tall | BRA | 0.007 | 0.993 | 0.005 | 0.010 | 0.031 | 0.006 | 0.948 |
| 1016 | 15(B1) | Tall | Brazilian Tall | BRA | 0.017 | 0.983 | 0.022 | 0.013 | 0.031 | 0.032 | 0.902 |
| 1017 | 15(B1) | Tall | Brazilian Tall | BRA | 0.006 | 0.994 | 0.008 | 0.007 | 0.006 | 0.007 | 0.973 |
| 1018 | 15(B1) | Tall | Brazilian Tall | BRA | 0.020 | 0.980 | 0.024 | 0.008 | 0.021 | 0.027 | 0.920 |
| 1019 | 15(B1) | Tall | Brazilian Tall | BRA | 0.011 | 0.989 | 0.010 | 0.013 | 0.006 | 0.012 | 0.959 |
| 1020 | 15(B1) | Tall | Brazilian Tall | BRA | 0.005 | 0.995 | 0.004 | 0.005 | 0.003 | 0.004 | 0.984 |
| 1021 | 15(B1) | Tall | Brazilian Tall | BRA | 0.006 | 0.994 | 0.006 | 0.009 | 0.004 | 0.005 | 0.976 |
| 1022 | 15(B1) | Tall | Brazilian Tall | BRA | 0.011 | 0.989 | 0.006 | 0.060 | 0.005 | 0.007 | 0.921 |
| 1023 | 15(B1) | Tall | Brazilian Tall | BRA | 0.011 | 0.989 | 0.007 | 0.048 | 0.005 | 0.007 | 0.933 |
| 1024 | 15(B1) | Tall | Brazilian Tall | BRA | 0.007 | 0.993 | 0.005 | 0.020 | 0.004 | 0.006 | 0.964 |
| 1025 | 15(B1) | Tall | Brazilian Tall | BRA | 0.005 | 0.995 | 0.004 | 0.008 | 0.003 | 0.004 | 0.981 |
| 1026 | 15(B1) | Tall | Brazilian Tall | BRA | 0.005 | 0.995 | 0.004 | 0.008 | 0.003 | 0.004 | 0.981 |
| 1027 | 15(B1) | Tall | Brazilian Tall | BRA | 0.009 | 0.991 | 0.006 | 0.020 | 0.005 | 0.007 | 0.961 |
| 1028 | 15(B1) | Tall | Brazilian Tall | BRA | 0.009 | 0.991 | 0.007 | 0.012 | 0.005 | 0.009 | 0.967 |
| 1029 | 15(B1) | Tall | Brazilian Tall | BRA | 0.013 | 0.987 | 0.007 | 0.058 | 0.006 | 0.009 | 0.920 |
| 1030 | 15(B1) | Tall | Brazilian Tall | BRA | 0.009 | 0.991 | 0.007 | 0.041 | 0.004 | 0.006 | 0.942 |
| 1031 | 15(B1) | Tall | Brazilian Tall | BRA | 0.007 | 0.993 | 0.005 | 0.008 | 0.003 | 0.006 | 0.977 |
| 1032 | 15(B1) | Tall | Brazilian Tall | BRA | 0.007 | 0.993 | 0.005 | 0.008 | 0.003 | 0.006 | 0.978 |
| 1033 | 15(B1) | Tall | Brazilian Tall | BRA | 0.007 | 0.993 | 0.004 | 0.009 | 0.005 | 0.006 | 0.974 |
| 1034 | 15(B1) | Tall | Brazilian Tall | BRA | 0.005 | 0.995 | 0.005 | 0.003 | 0.007 | 0.005 | 0.980 |
| 1035 | 15(B1) | Tall | Brazilian Tall | BRA | 0.007 | 0.993 | 0.006 | 0.006 | 0.005 | 0.006 | 0.978 |
| 1036 | 15(B1) | Tall | Brazilian Tall | BRA | 0.008 | 0.992 | 0.006 | 0.010 | 0.004 | 0.008 | 0.973 |
| 1037 | 15(B1) | Tall | Brazilian Tall | BRA | 0.008 | 0.992 | 0.006 | 0.006 | 0.004 | 0.010 | 0.973 |
| 1038 | 15(B1) | Tall | Brazilian Tall | BRA | 0.007 | 0.993 | 0.006 | 0.008 | 0.005 | 0.006 | 0.975 |
| 1039 | 15(B1) | Tall | Brazilian Tall | BRA | 0.006 | 0.994 | 0.006 | 0.009 | 0.004 | 0.005 | 0.977 |
| 1040 | 15(B1) | Tall | Brazilian Tall | BRA | 0.007 | 0.993 | 0.005 | 0.006 | 0.007 | 0.007 | 0.976 |
| 1041 | 15(B1) | Tall | Brazilian Tall | BRA | 0.009 | 0.991 | 0.006 | 0.021 | 0.006 | 0.006 | 0.961 |
| 1042 | 15(B1) | Tall | Brazilian Tall | BRA | 0.013 | 0.987 | 0.008 | 0.035 | 0.007 | 0.010 | 0.939 |
| 1043 | 15(B1) | Tall | Brazilian Tall | BRA | 0.010 | 0.990 | 0.008 | 0.013 | 0.010 | 0.009 | 0.961 |
| 1044 | 15(B1) | Tall | Brazilian Tall | BRA | 0.057 | 0.943 | 0.034 | 0.022 | 0.005 | 0.015 | 0.924 |
| 1045 | 15(B1) | Tall | Brazilian Tall | BRA | 0.011 | 0.989 | 0.009 | 0.016 | 0.004 | 0.009 | 0.962 |
| 1046 | 15(B1) | Tall | Brazilian Tall | BRA | 0.006 | 0.994 | 0.004 | 0.008 | 0.004 | 0.005 | 0.979 |
| 1047 | 15(B1) | Tall | Brazilian Tall | BRA | 0.055 | 0.945 | 0.045 | 0.008 | 0.004 | 0.020 | 0.924 |
| 1048 | 15(B1) | Tall | Brazilian Tall | BRA | 0.005 | 0.995 | 0.005 | 0.003 | 0.004 | 0.004 | 0.984 |
| 1049 | 15(B1) | Tall | Brazilian Tall | BRA | 0.008 | 0.992 | 0.005 | 0.021 | 0.005 | 0.006 | 0.963 |
| 1050 | 15(B1) | Tall | Brazilian Tall | BRA | 0.009 | 0.991 | 0.006 | 0.029 | 0.005 | 0.007 | 0.954 |
| 1051 | 15(B1) | Tall | Brazilian Tall | BRA | 0.010 | 0.990 | 0.012 | 0.012 | 0.009 | 0.009 | 0.959 |
| 1052 | 15(B1) | Tall | Brazilian Tall | BRA | 0.009 | 0.991 | 0.011 | 0.007 | 0.026 | 0.011 | 0.946 |
| 1053 | 15(B1) | Tall | Brazilian Tall | BRA | 0.011 | 0.989 | 0.009 | 0.028 | 0.008 | 0.008 | 0.947 |
| 1054 | 15(B1) | Tall | Brazilian Tall | BRA | 0.008 | 0.992 | 0.008 | 0.011 | 0.005 | 0.006 | 0.969 |
| 1055 | 15(B1) | Tall | Brazilian Tall | BRA | 0.011 | 0.989 | 0.012 | 0.016 | 0.009 | 0.017 | 0.945 |
| 1056 | 15(B1) | Tall | Brazilian Tall | BRA | 0.012 | 0.988 | 0.016 | 0.010 | 0.014 | 0.020 | 0.940 |
| 1057 | 15(B1) | Tall | Brazilian Tall | BRA | 0.341 | 0.659 | 0.024 | 0.016 | 0.358 | 0.058 | 0.545 |
| 1058 | 15(B1) | Tall | Brazilian Tall | BRA | 0.639 | 0.361 | 0.028 | 0.045 | 0.495 | 0.085 | 0.347 |
| 1059 | 15(B1) | Tall | Brazilian Tall | BRA | 0.057 | 0.943 | 0.026 | 0.146 | 0.036 | 0.027 | 0.764 |
| 1060 | 15(B1) | Tall | Brazilian Tall | BRA | 0.006 | 0.994 | 0.007 | 0.004 | 0.009 | 0.006 | 0.975 |
| 1061 | 15(B1) | Tall | Brazilian Tall | BRA | 0.447 | 0.553 | 0.032 | 0.017 | 0.310 | 0.169 | 0.472 |
| 1062 | 15(B1) | Tall | Brazilian Tall | BRA | 0.008 | 0.992 | 0.009 | 0.007 | 0.005 | 0.007 | 0.972 |
| 1063 | 15(B1) | Tall | Brazilian Tall | BRA | 0.075 | 0.925 | 0.055 | 0.046 | 0.035 | 0.127 | 0.737 |
| 1064 | 16(B2) | Tall | Comoro Moheli Tall | COM | 0.533 | 0.467 | 0.025 | 0.030 | 0.482 | 0.049 | 0.414 |
| 1065 | 16(B2) | Tall | Comoro Moheli Tall | COM | 0.421 | 0.579 | 0.021 | 0.043 | 0.271 | 0.170 | 0.494 |
| 1066 | 16(B2) | Tall | Comoro Moheli Tall | COM | 0.182 | 0.818 | 0.025 | 0.015 | 0.250 | 0.056 | 0.655 |
| 1067 | 16(B2) | Tall | Comoro Moheli Tall | COM | 0.493 | 0.507 | 0.016 | 0.010 | 0.521 | 0.076 | 0.378 |
| 1068 | 16(B2) | Tall | Comoro Moheli Tall | COM | 0.451 | 0.549 | 0.016 | 0.008 | 0.519 | 0.040 | 0.417 |
| 1069 | 16(B2) | Tall | Mozambique Tall | MOZ | 0.047 | 0.953 | 0.013 | 0.138 | 0.084 | 0.022 | 0.743 |
| 1070 | 16(B2) | Tall | Mozambique Tall | MOZ | 0.019 | 0.981 | 0.010 | 0.137 | 0.021 | 0.009 | 0.822 |
| 1071 | 16(B2) | Tall | Mozambique Tall | MOZ | 0.059 | 0.941 | 0.043 | 0.055 | 0.107 | 0.044 | 0.750 |
| 1072 | 16(B2) | Tall | Mozambique Tall | MOZ | 0.028 | 0.972 | 0.029 | 0.080 | 0.028 | 0.016 | 0.847 |
| 1073 | 16(B2) | Tall | Mozambique Tall | MOZ | 0.070 | 0.930 | 0.055 | 0.008 | 0.039 | 0.154 | 0.744 |
| 1074 | 16(B2) | Tall | Mozambique Tall | MOZ | 0.182 | 0.818 | 0.033 | 0.006 | 0.012 | 0.246 | 0.703 |
| 1075 | 16(B2) | Tall | Andaman Ordinary Tall | IND | 0.381 | 0.619 | 0.077 | 0.010 | 0.034 | 0.309 | 0.569 |
| 1076 | 16(B2) | Tall | Andaman Ordinary Tall | IND | 0.475 | 0.525 | 0.025 | 0.010 | 0.034 | 0.475 | 0.456 |
| 1077 | 16(B2) | Tall | Andaman Ordinary Tall | IND | 0.082 | 0.918 | 0.016 | 0.016 | 0.268 | 0.035 | 0.665 |
| 1078 | 16(B2) | Tall | Andaman Ordinary Tall | IND | 0.326 | 0.674 | 0.226 | 0.006 | 0.027 | 0.090 | 0.651 |
| 1079 | 16(B2) | Tall | Andaman Ordinary Tall | IND | 0.127 | 0.873 | 0.023 | 0.008 | 0.200 | 0.080 | 0.690 |
| 1080 | 16(B2) | Tall | Kappadam Tall | IND | 0.015 | 0.985 | 0.014 | 0.040 | 0.015 | 0.015 | 0.917 |
| 1081 | 16(B2) | Tall | Kappadam Tall | IND | 0.047 | 0.953 | 0.020 | 0.019 | 0.160 | 0.052 | 0.749 |
| 1082 | 16(B2) | Tall | Kappadam Tall | IND | 0.016 | 0.984 | 0.017 | 0.010 | 0.043 | 0.035 | 0.895 |
| 1083 | 16(B2) | Tall | Kappadam Tall | IND | 0.009 | 0.991 | 0.009 | 0.048 | 0.019 | 0.010 | 0.914 |
| 1084 | 16(B2) | Tall | Kappadam Tall | IND | 0.018 | 0.982 | 0.010 | 0.007 | 0.187 | 0.016 | 0.780 |
| 1085 | 16(B2) | Tall | Sri Lanka Tall Margaret | LKA | 0.080 | 0.920 | 0.025 | 0.108 | 0.029 | 0.088 | 0.751 |
| 1086 | 16(B2) | Tall | Sri Lanka Tall Margaret | LKA | 0.435 | 0.565 | 0.018 | 0.079 | 0.067 | 0.336 | 0.500 |
| 1087 | 16(B2) | Tall | East African Tall Kenya | TZA | 0.022 | 0.978 | 0.017 | 0.021 | 0.072 | 0.033 | 0.857 |
| 1088 | 16(B2) | Tall | East African Tall Kenya | TZA | 0.008 | 0.992 | 0.012 | 0.021 | 0.015 | 0.013 | 0.939 |
| 1089 | 16(B2) | Tall | East African Tall Kenya | TZA | 0.039 | 0.961 | 0.017 | 0.021 | 0.156 | 0.035 | 0.771 |
| 1090 | 16(B2) | Tall | East African Tall Kenya | TZA | 0.027 | 0.973 | 0.022 | 0.018 | 0.145 | 0.018 | 0.797 |
| 1091 | 16(B2) | Tall | Kappadam Tall | IND | 0.004 | 0.996 | 0.004 | 0.003 | 0.003 | 0.004 | 0.986 |
| 1092 | 16(B2) | Tall | Indian East Coast Tall | IND | 0.044 | 0.956 | 0.037 | 0.010 | 0.080 | 0.080 | 0.793 |
| 1093 | 16(B2) | Tall | Indian East Coast Tall | IND | 0.010 | 0.990 | 0.015 | 0.017 | 0.007 | 0.018 | 0.943 |
| 1094 | 16(B2) | Tall | Indian East Coast Tall | IND | 0.364 | 0.636 | 0.016 | 0.009 | 0.141 | 0.265 | 0.569 |
| 1095 | 16(B2) | Tall | Indian East Coast Tall | IND | 0.010 | 0.990 | 0.011 | 0.008 | 0.005 | 0.012 | 0.965 |
| 1096 | 16(B2) | Tall | Indian East Coast Tall | IND | 0.044 | 0.956 | 0.037 | 0.010 | 0.077 | 0.081 | 0.794 |
| 1097 | 16(B2) | Tall | Indian East Coast Tall | IND | 0.061 | 0.939 | 0.055 | 0.006 | 0.006 | 0.058 | 0.875 |
| 1098 | 16(B2) | Tall | Indian East Coast Tall | IND | 0.640 | 0.360 | 0.027 | 0.078 | 0.237 | 0.340 | 0.318 |
| 1099 | 16(B2) | Tall | East African Tall | TZA | 0.025 | 0.975 | 0.021 | 0.014 | 0.047 | 0.064 | 0.855 |
| 1100 | 16(B2) | Tall | East African Tall | TZA | 0.110 | 0.890 | 0.058 | 0.044 | 0.109 | 0.068 | 0.721 |
| 1101 | 16(B2) | Tall | East African Tall | TZA | 0.523 | 0.477 | 0.040 | 0.016 | 0.472 | 0.067 | 0.405 |
| 1102 | 16(B2) | Tall | East African Tall | TZA | 0.062 | 0.938 | 0.048 | 0.010 | 0.133 | 0.051 | 0.758 |
| 1103 | 16(B2) | Tall | Comoro Moheli Tall | COM | 0.460 | 0.540 | 0.236 | 0.025 | 0.121 | 0.134 | 0.485 |
| 1104 | 16(B2) | Tall | Comoro Moheli Tall | COM | 0.085 | 0.915 | 0.024 | 0.009 | 0.227 | 0.139 | 0.601 |
| 1105 | 16(B2) | Tall | Mozambique Tall | MOZ | 0.055 | 0.945 | 0.010 | 0.020 | 0.229 | 0.031 | 0.710 |
| 1106 | 16(B2) | Tall | Mozambique Tall | MOZ | 0.112 | 0.888 | 0.057 | 0.058 | 0.133 | 0.063 | 0.688 |
| 1107 | 16(B2) | Tall | Laccadives Ordinary Tall | IND | 0.006 | 0.994 | 0.006 | 0.004 | 0.005 | 0.006 | 0.979 |
| 1108 | 16(B2) | Tall | Laccadives Ordinary Tall | IND | 0.016 | 0.984 | 0.009 | 0.009 | 0.142 | 0.015 | 0.824 |
| 1109 | 16(B2) | Tall | Laccadives Ordinary Tall | IND | 0.079 | 0.921 | 0.013 | 0.034 | 0.246 | 0.012 | 0.694 |
| 1110 | 16(B2) | Tall | East African Tall LBS | TZA | 0.081 | 0.919 | 0.020 | 0.010 | 0.194 | 0.072 | 0.705 |
| 1111 | 16(B2) | Tall | East African Tall LBS | TZA | 0.032 | 0.968 | 0.024 | 0.008 | 0.144 | 0.033 | 0.790 |
| 1112 | 16(B2) | Tall | East African Tall LBS | TZA | 0.011 | 0.989 | 0.011 | 0.034 | 0.036 | 0.010 | 0.909 |
| 1113 | 16(B2) | Tall | East African Tall LBS | TZA | 0.120 | 0.880 | 0.079 | 0.045 | 0.032 | 0.087 | 0.756 |
| 1114 | 16(B2) | Tall | East African Tall LBS | TZA | 0.212 | 0.788 | 0.034 | 0.042 | 0.111 | 0.184 | 0.629 |
| 1115 | 16(B2) | Tall | East African Tall Kimanga | TZA | 0.006 | 0.994 | 0.005 | 0.004 | 0.005 | 0.006 | 0.980 |
| 1116 | 16(B2) | Tall | East African Tall Kimanga | TZA | 0.026 | 0.974 | 0.035 | 0.013 | 0.066 | 0.068 | 0.818 |
| 1117 | 16(B2) | Tall | East African Tall Kimanga | TZA | 0.136 | 0.864 | 0.034 | 0.032 | 0.091 | 0.167 | 0.677 |
| 1118 | 16(B2) | Tall | East African Tall Kimanga | TZA | 0.074 | 0.926 | 0.011 | 0.012 | 0.298 | 0.013 | 0.666 |
| 1119 | 16(B2) | Tall | East African Tall Kimanga | TZA | 0.189 | 0.811 | 0.052 | 0.021 | 0.151 | 0.117 | 0.659 |
| 1120 | 16(B2) | Tall | Mozambique Tall | MOZ | 0.100 | 0.900 | 0.037 | 0.013 | 0.074 | 0.183 | 0.693 |
| 1121 | 16(B2) | Tall | Mozambique Tall | MOZ | 0.025 | 0.975 | 0.036 | 0.015 | 0.034 | 0.035 | 0.879 |
| 1122 | 16(B2) | Tall | Mozambique Tall | MOZ | 0.015 | 0.985 | 0.009 | 0.012 | 0.136 | 0.010 | 0.833 |
| 1123 | 16(B2) | Tall | Mozambique Tall | MOZ | 0.340 | 0.660 | 0.055 | 0.100 | 0.193 | 0.079 | 0.572 |
| 1124 | 16(B2) | Tall | Mozambique Tall | MOZ | 0.034 | 0.966 | 0.020 | 0.089 | 0.054 | 0.023 | 0.814 |
| 1125 | 16(B2) | Tall | Mozambique Tall | MOZ | 0.397 | 0.603 | 0.261 | 0.063 | 0.064 | 0.059 | 0.554 |
| 1126 | 16(B2) | Tall | Mozambique Tall | MOZ | 0.090 | 0.910 | 0.029 | 0.050 | 0.035 | 0.141 | 0.745 |
| 1127 | 16(B2) | Tall | Mozambique Tall | MOZ | 0.129 | 0.871 | 0.054 | 0.019 | 0.017 | 0.209 | 0.701 |
| 1128 | 16(B2) | Tall | Mozambique Tall | MOZ | 0.073 | 0.927 | 0.101 | 0.037 | 0.050 | 0.060 | 0.752 |
| 1129 | 16(B2) | Tall | Mozambique Tall | MOZ | 0.052 | 0.948 | 0.082 | 0.070 | 0.017 | 0.027 | 0.804 |
| 1130 | 16(B2) | Tall | Mozambique Tall | MOZ | 0.045 | 0.955 | 0.048 | 0.066 | 0.026 | 0.043 | 0.817 |
| 1131 | 16(B2) | Tall | Mozambique Tall | MOZ | 0.170 | 0.830 | 0.008 | 0.058 | 0.345 | 0.022 | 0.567 |
| 1132 | 16(B2) | Tall | Mozambique Tall | MOZ | 0.016 | 0.984 | 0.022 | 0.015 | 0.057 | 0.022 | 0.885 |
| 1133 | 16(B2) | Tall | Mozambique Tall | MOZ | 0.163 | 0.837 | 0.038 | 0.025 | 0.130 | 0.173 | 0.634 |
| 1134 | 16(B2) | Tall | Mozambique Tall | MOZ | 0.413 | 0.587 | 0.012 | 0.026 | 0.411 | 0.049 | 0.502 |
| 1135 | 16(B2) | Tall | Mozambique Tall | MOZ | 0.022 | 0.978 | 0.028 | 0.032 | 0.044 | 0.032 | 0.864 |
| 1136 | 16(B2) | Tall | Mozambique Tall | MOZ | 0.125 | 0.875 | 0.013 | 0.240 | 0.015 | 0.024 | 0.708 |
| 1137 | 16(B2) | Tall | Mozambique Tall | MOZ | 0.041 | 0.959 | 0.030 | 0.045 | 0.081 | 0.043 | 0.801 |
| 1138 | 16(B2) | Tall | Mozambique Tall | MOZ | 0.287 | 0.713 | 0.019 | 0.161 | 0.183 | 0.061 | 0.576 |
| 1139 | 16(B2) | Tall | Mozambique Tall | MOZ | 0.178 | 0.822 | 0.045 | 0.010 | 0.201 | 0.078 | 0.666 |
| 1140 | 16(B2) | Tall | Mozambique Tall | MOZ | 0.071 | 0.929 | 0.009 | 0.104 | 0.129 | 0.011 | 0.746 |
| 1141 | 16(B2) | Tall | Mozambique Tall | MOZ | 0.013 | 0.987 | 0.009 | 0.038 | 0.032 | 0.009 | 0.911 |
| 1142 | 16(B2) | Tall | Mozambique Tall | MOZ | 0.062 | 0.938 | 0.024 | 0.027 | 0.173 | 0.039 | 0.737 |
| 1143 | 16(B2) | Tall | Mozambique Tall | MOZ | 0.009 | 0.991 | 0.009 | 0.004 | 0.016 | 0.010 | 0.960 |
| 1144 | 16(B2) | Tall | Mozambique Tall | MOZ | 0.045 | 0.955 | 0.052 | 0.041 | 0.010 | 0.085 | 0.812 |
| 1145 | 16(B2) | Tall | Mozambique Tall | MOZ | 0.128 | 0.872 | 0.016 | 0.020 | 0.258 | 0.054 | 0.653 |
| 1146 | 16(B2) | Tall | Mozambique Tall | MOZ | 0.125 | 0.875 | 0.053 | 0.092 | 0.066 | 0.092 | 0.697 |
| 1147 | 16(B2) | Tall | Mozambique Tall | MOZ | 0.030 | 0.970 | 0.016 | 0.015 | 0.106 | 0.045 | 0.817 |
| 1148 | 16(B2) | Tall | Mozambique Tall | MOZ | 0.161 | 0.839 | 0.021 | 0.061 | 0.237 | 0.023 | 0.658 |
| 1149 | 16(B2) | Tall | Mozambique Tall | MOZ | 0.018 | 0.982 | 0.023 | 0.034 | 0.014 | 0.020 | 0.910 |
| 1150 | 16(B2) | Tall | Mozambique Tall | MOZ | 0.389 | 0.611 | 0.012 | 0.039 | 0.427 | 0.023 | 0.499 |
| 1151 | 16(B2) | Tall | Mozambique Tall | MOZ | 0.060 | 0.940 | 0.053 | 0.037 | 0.049 | 0.096 | 0.765 |
| 1152 | 16(B2) | Tall | Mozambique Tall | MOZ | 0.041 | 0.959 | 0.006 | 0.126 | 0.111 | 0.015 | 0.741 |
| 1153 | 16(B2) | Tall | Mozambique Tall | MOZ | 0.051 | 0.949 | 0.006 | 0.188 | 0.050 | 0.009 | 0.748 |
| 1154 | 16(B2) | Tall | Mozambique Tall | MOZ | 0.007 | 0.993 | 0.007 | 0.005 | 0.005 | 0.007 | 0.976 |
| 1155 | 16(B2) | Tall | Mozambique Tall | MOZ | 0.137 | 0.863 | 0.080 | 0.033 | 0.032 | 0.161 | 0.695 |
| 1156 | 16(B2) | Tall | Mozambique Tall | MOZ | 0.031 | 0.969 | 0.030 | 0.019 | 0.044 | 0.062 | 0.845 |
| 1157 | 16(B2) | Tall | Mozambique Tall | MOZ | 0.255 | 0.745 | 0.007 | 0.031 | 0.414 | 0.026 | 0.521 |
| 1158 | 16(B2) | Tall | Mozambique Tall | MOZ | 0.064 | 0.936 | 0.009 | 0.153 | 0.104 | 0.025 | 0.709 |
| 1159 | 16(B2) | Tall | Mozambique Tall | MOZ | 0.049 | 0.951 | 0.025 | 0.135 | 0.056 | 0.025 | 0.758 |
| 1160 | 16(B2) | Tall | Mozambique Tall | MOZ | 0.015 | 0.985 | 0.020 | 0.019 | 0.025 | 0.026 | 0.909 |
| 1161 | 16(B2) | Tall | Mozambique Tall | MOZ | 0.084 | 0.916 | 0.005 | 0.255 | 0.050 | 0.009 | 0.680 |
| 1162 | 16(B2) | Tall | Mozambique Tall | MOZ | 0.017 | 0.983 | 0.024 | 0.038 | 0.014 | 0.015 | 0.909 |
| 1163 | 16(B2) | Tall | Mozambique Tall | MOZ | 0.396 | 0.604 | 0.051 | 0.334 | 0.050 | 0.037 | 0.529 |
| 1164 | 16(B2) | Tall | Mozambique Tall | MOZ | 0.115 | 0.885 | 0.053 | 0.011 | 0.171 | 0.081 | 0.683 |
| 1165 | 16(B2) | Tall | Mozambique Tall | MOZ | 0.073 | 0.927 | 0.030 | 0.130 | 0.032 | 0.089 | 0.719 |
| 1166 | 16(B2) | Tall | Mozambique Tall | MOZ | 0.030 | 0.970 | 0.006 | 0.210 | 0.012 | 0.009 | 0.763 |
| 1167 | 16(B2) | Tall | Mozambique Tall | MOZ | 0.036 | 0.964 | 0.033 | 0.080 | 0.075 | 0.016 | 0.796 |
| 1168 | 16(B2) | Tall | Mozambique Tall | MOZ | 0.007 | 0.993 | 0.006 | 0.011 | 0.007 | 0.006 | 0.971 |
| 1169 | 16(B2) | Tall | Mozambique Tall | MOZ | 0.072 | 0.928 | 0.010 | 0.058 | 0.194 | 0.018 | 0.720 |
| 1170 | 16(B2) | Tall | Mozambique Tall | MOZ | 0.026 | 0.974 | 0.015 | 0.007 | 0.124 | 0.050 | 0.805 |
| 1171 | 16(B2) | Tall | Mozambique Tall | MOZ | 0.255 | 0.745 | 0.018 | 0.045 | 0.281 | 0.062 | 0.595 |
| 1172 | 16(B2) | Tall | Mozambique Tall | MOZ | 0.350 | 0.650 | 0.044 | 0.008 | 0.372 | 0.059 | 0.517 |
| 1173 | 16(B2) | Tall | Mozambique Tall | MOZ | 0.556 | 0.444 | 0.016 | 0.011 | 0.574 | 0.017 | 0.383 |
| 1174 | 16(B2) | Tall | Mozambique Tall | MOZ | 0.142 | 0.858 | 0.041 | 0.078 | 0.116 | 0.087 | 0.678 |
| 1175 | 16(B2) | Tall | Mozambique Tall | MOZ | 0.365 | 0.635 | 0.216 | 0.012 | 0.106 | 0.089 | 0.578 |
| 1176 | 16(B2) | Tall | Mozambique Tall | MOZ | 0.049 | 0.951 | 0.053 | 0.056 | 0.059 | 0.045 | 0.787 |
| 1177 | 16(B2) | Tall | Mozambique Tall | MOZ | 0.303 | 0.697 | 0.008 | 0.010 | 0.482 | 0.011 | 0.488 |
| 1178 | 16(B2) | Tall | Mozambique Tall | MOZ | 0.322 | 0.678 | 0.148 | 0.093 | 0.025 | 0.165 | 0.569 |
| 1179 | 16(B2) | Tall | Mozambique Tall | MOZ | 0.860 | 0.140 | 0.036 | 0.020 | 0.599 | 0.230 | 0.116 |
| 1180 | 16(B2) | Tall | Mozambique Tall | MOZ | 0.093 | 0.907 | 0.018 | 0.027 | 0.173 | 0.061 | 0.721 |
| 1181 | 16(B2) | Tall | Mozambique Tall | MOZ | 0.331 | 0.669 | 0.019 | 0.012 | 0.386 | 0.071 | 0.511 |
| 1182 | 16(B2) | Tall | Mozambique Tall | MOZ | 0.122 | 0.878 | 0.107 | 0.019 | 0.013 | 0.122 | 0.740 |
| 1183 | 16(B2) | Tall | Mozambique Tall | MOZ | 0.196 | 0.804 | 0.022 | 0.007 | 0.374 | 0.020 | 0.578 |
| 1184 | 16(B2) | Tall | Mozambique Tall | MOZ | 0.093 | 0.907 | 0.029 | 0.036 | 0.142 | 0.079 | 0.715 |
| 1185 | 16(B2) | Tall | Mozambique Tall | MOZ | 0.009 | 0.991 | 0.009 | 0.005 | 0.032 | 0.021 | 0.933 |
| 1186 | 16(B2) | Tall | Mozambique Tall | MOZ | 0.153 | 0.847 | 0.092 | 0.094 | 0.093 | 0.042 | 0.678 |
| 1187 | 16(B2) | Tall | Mozambique Tall | MOZ | 0.074 | 0.926 | 0.067 | 0.069 | 0.104 | 0.030 | 0.730 |
| 1188 | 16(B2) | Tall | Mozambique Tall | MOZ | 0.035 | 0.965 | 0.010 | 0.074 | 0.102 | 0.019 | 0.794 |
| 1189 | 16(B2) | Tall | Mozambique Tall | MOZ | 0.171 | 0.829 | 0.062 | 0.008 | 0.248 | 0.036 | 0.646 |
| 1190 | 16(B2) | Tall | Mozambique Tall | MOZ | 0.010 | 0.990 | 0.015 | 0.021 | 0.022 | 0.016 | 0.926 |
| 1191 | 16(B2) | Tall | Mozambique Tall | MOZ | 0.424 | 0.576 | 0.008 | 0.017 | 0.505 | 0.012 | 0.459 |
| 1192 | 16(B2) | Tall | Mozambique Tall | MOZ | 0.056 | 0.944 | 0.019 | 0.025 | 0.133 | 0.077 | 0.746 |
| 1193 | 16(B2) | Tall | Mozambique Tall | MOZ | 0.090 | 0.910 | 0.058 | 0.016 | 0.032 | 0.185 | 0.709 |
| 1194 | 16(B2) | Tall | Mozambique Tall | MOZ | 0.015 | 0.985 | 0.016 | 0.035 | 0.011 | 0.016 | 0.923 |
| 1195 | 16(B2) | Tall | Mozambique Tall | MOZ | 0.189 | 0.811 | 0.013 | 0.020 | 0.360 | 0.038 | 0.569 |
| 1196 | 16(B2) | Tall | Mozambique Tall | MOZ | 0.425 | 0.575 | 0.008 | 0.016 | 0.506 | 0.012 | 0.458 |
| 1197 | 16(B2) | Tall | Mozambique Tall | MOZ | 0.151 | 0.849 | 0.042 | 0.162 | 0.089 | 0.057 | 0.650 |
| 1198 | 16(B2) | Tall | Mozambique Tall | MOZ | 0.107 | 0.893 | 0.031 | 0.119 | 0.025 | 0.101 | 0.723 |
| 1199 | 16(B2) | Tall | Mozambique Tall | MOZ | 0.474 | 0.526 | 0.007 | 0.535 | 0.009 | 0.008 | 0.440 |
| 1200 | 16(B2) | Tall | Mozambique Tall | MOZ | 0.502 | 0.498 | 0.008 | 0.562 | 0.009 | 0.007 | 0.415 |
| 1201 | 16(B2) | Tall | Mozambique Tall | MOZ | 0.475 | 0.525 | 0.007 | 0.536 | 0.009 | 0.007 | 0.440 |
| 1202 | 16(B2) | Tall | Mozambique Tall | MOZ | 0.551 | 0.449 | 0.007 | 0.595 | 0.009 | 0.007 | 0.382 |
| 1203 | 16(B2) | Tall | Mozambique Tall | MOZ | 0.277 | 0.723 | 0.093 | 0.039 | 0.077 | 0.159 | 0.632 |
| 1204 | 16(B2) | Tall | Mozambique Tall | MOZ | 0.046 | 0.954 | 0.019 | 0.161 | 0.038 | 0.019 | 0.764 |
| 1205 | 16(B2) | Tall | Mozambique Tall | MOZ | 0.011 | 0.989 | 0.010 | 0.012 | 0.018 | 0.014 | 0.946 |
| 1206 | 16(B2) | Tall | Mozambique Tall | MOZ | 0.032 | 0.968 | 0.016 | 0.064 | 0.045 | 0.044 | 0.832 |
| 1207 | 16(B2) | Tall | Mozambique Tall | MOZ | 0.021 | 0.979 | 0.018 | 0.036 | 0.032 | 0.032 | 0.882 |
| 1208 | 16(B2) | Tall | Mozambique Tall | MOZ | 0.156 | 0.844 | 0.018 | 0.187 | 0.090 | 0.033 | 0.672 |
| 1209 | 16(B2) | Tall | Mozambique Tall | MOZ | 0.016 | 0.984 | 0.016 | 0.029 | 0.109 | 0.016 | 0.830 |
| 1210 | 16(B2) | Tall | Mozambique Tall | MOZ | 0.014 | 0.986 | 0.018 | 0.030 | 0.050 | 0.017 | 0.885 |
| 1211 | 17— | Tall | Comoros | COM | 0.496 | 0.504 | 0.016 | 0.138 | 0.400 | 0.040 | 0.406 |
| 1212 | 17— | Tall | Comoros | COM | 0.476 | 0.524 | 0.025 | 0.017 | 0.474 | 0.056 | 0.428 |
| 1213 | 17— | Tall | Comoros | COM | 0.633 | 0.367 | 0.048 | 0.011 | 0.398 | 0.253 | 0.290 |
| 1214 | 17— | Tall | Comoros | COM | 0.041 | 0.959 | 0.045 | 0.019 | 0.074 | 0.036 | 0.825 |
| 1215 | 17— | Tall | Comoros | COM | 0.294 | 0.706 | 0.048 | 0.039 | 0.287 | 0.038 | 0.587 |
| 1216 | 17— | Tall | Comoros | COM | 0.155 | 0.845 | 0.048 | 0.066 | 0.211 | 0.063 | 0.613 |
| 1217 | 17— | Tall | Comoros | COM | 0.790 | 0.210 | 0.016 | 0.014 | 0.797 | 0.023 | 0.150 |
| 1218 | 17— | Tall | Comoros | COM | 0.255 | 0.745 | 0.080 | 0.017 | 0.179 | 0.142 | 0.583 |
| 1219 | 17— | Tall | Comoros | COM | 0.551 | 0.449 | 0.039 | 0.192 | 0.262 | 0.088 | 0.419 |
| 1220 | 17— | Tall | Comoros | COM | 0.144 | 0.856 | 0.100 | 0.017 | 0.062 | 0.167 | 0.654 |
| 1221 | 17— | Tall | Comoros | COM | 0.519 | 0.481 | 0.281 | 0.032 | 0.022 | 0.209 | 0.456 |
| 1222 | 17— | Tall | Comoros | COM | 0.780 | 0.220 | 0.033 | 0.026 | 0.613 | 0.131 | 0.197 |
| 1223 | 17— | Tall | Comoros | COM | 0.397 | 0.603 | 0.015 | 0.043 | 0.337 | 0.137 | 0.468 |
| 1224 | 18— | Tall | Madagascar | MAD | 0.006 | 0.994 | 0.007 | 0.004 | 0.021 | 0.006 | 0.961 |
| 1225 | 18— | Tall | Madagascar | MAD | 0.031 | 0.969 | 0.036 | 0.051 | 0.067 | 0.127 | 0.720 |
| 1226 | 18— | Tall | Madagascar | MAD | 0.075 | 0.925 | 0.036 | 0.010 | 0.275 | 0.040 | 0.638 |
| 1227 | 18— | Tall | Madagascar | MAD | 0.616 | 0.384 | 0.168 | 0.038 | 0.066 | 0.388 | 0.340 |
| 1228 | 18— | Tall | Madagascar | MAD | 0.009 | 0.991 | 0.007 | 0.007 | 0.007 | 0.008 | 0.971 |
| 1229 | 18— | Tall | Madagascar | MAD | 0.164 | 0.836 | 0.016 | 0.010 | 0.247 | 0.091 | 0.636 |
| 1230 | 18— | Tall | Madagascar | MAD | 0.064 | 0.936 | 0.108 | 0.017 | 0.036 | 0.068 | 0.772 |
| 1231 | 18— | Tall | Madagascar | MAD | 0.033 | 0.967 | 0.015 | 0.101 | 0.055 | 0.036 | 0.792 |
| 1232 | 18— | Tall | Madagascar | MAD | 0.043 | 0.957 | 0.029 | 0.026 | 0.030 | 0.100 | 0.815 |
| 1233 | 18— | Tall | Madagascar | MAD | 0.007 | 0.993 | 0.006 | 0.007 | 0.006 | 0.007 | 0.974 |
| 1234 | 18— | Tall | Madagascar | MAD | 0.492 | 0.508 | 0.046 | 0.329 | 0.070 | 0.109 | 0.446 |
| 1235 | 18— | Tall | Madagascar | MAD | 0.490 | 0.510 | 0.039 | 0.105 | 0.148 | 0.262 | 0.446 |
| 1236 | 18— | Tall | Madagascar | MAD | 0.286 | 0.714 | 0.019 | 0.015 | 0.226 | 0.205 | 0.536 |
| 1237 | 18— | Tall | Madagascar | MAD | 0.548 | 0.452 | 0.019 | 0.018 | 0.424 | 0.163 | 0.376 |
| 1238 | 18— | Tall | Madagascar | MAD | 0.484 | 0.516 | 0.036 | 0.022 | 0.048 | 0.474 | 0.420 |
| 1239 | 18— | Tall | Madagascar | MAD | 0.190 | 0.810 | 0.013 | 0.079 | 0.186 | 0.144 | 0.578 |
| 1240 | 18— | Tall | Madagascar | MAD | 0.338 | 0.662 | 0.031 | 0.093 | 0.278 | 0.040 | 0.557 |
| 1241 | 18— | Tall | Madagascar | MAD | 0.462 | 0.538 | 0.022 | 0.047 | 0.431 | 0.082 | 0.418 |
| 1242 | 18— | Tall | Madagascar | MAD | 0.390 | 0.610 | 0.027 | 0.018 | 0.449 | 0.048 | 0.459 |
| 1243 | 18— | Tall | Madagascar | MAD | 0.639 | 0.361 | 0.108 | 0.244 | 0.125 | 0.186 | 0.337 |
| 1244 | 18— | Tall | Madagascar | MAD | 0.752 | 0.248 | 0.016 | 0.043 | 0.068 | 0.694 | 0.181 |
| 1245 | 18— | Tall | Madagascar | MAD | 0.347 | 0.653 | 0.048 | 0.067 | 0.109 | 0.207 | 0.569 |
| 1246 | 18— | Tall | Madagascar | MAD | 0.241 | 0.759 | 0.034 | 0.205 | 0.042 | 0.077 | 0.642 |
| 1247 | 18— | Tall | Madagascar | MAD | 0.334 | 0.666 | 0.008 | 0.016 | 0.395 | 0.029 | 0.552 |
| 1248 | 18— | Tall | Madagascar | MAD | 0.440 | 0.560 | 0.019 | 0.015 | 0.479 | 0.013 | 0.474 |
| 1249 | 18— | Tall | Madagascar | MAD | 0.269 | 0.731 | 0.017 | 0.023 | 0.400 | 0.041 | 0.519 |
| 1250 | 18— | Tall | Madagascar | MAD | 0.017 | 0.983 | 0.020 | 0.044 | 0.014 | 0.051 | 0.871 |
| 1251 | 18— | Tall | Madagascar | MAD | 0.483 | 0.517 | 0.021 | 0.031 | 0.445 | 0.049 | 0.454 |
| 1252 | 18— | Tall | Madagascar | MAD | 0.095 | 0.905 | 0.016 | 0.027 | 0.268 | 0.038 | 0.651 |
| 1253 | 18— | Tall | Madagascar | MAD | 0.235 | 0.765 | 0.030 | 0.058 | 0.317 | 0.054 | 0.541 |
| 1254 | 18— | Tall | Madagascar | MAD | 0.392 | 0.608 | 0.327 | 0.050 | 0.031 | 0.059 | 0.532 |
| 1255 | 18— | Tall | Madagascar | MAD | 0.388 | 0.612 | 0.098 | 0.052 | 0.270 | 0.141 | 0.439 |
| 1256 | 18— | Tall | Madagascar | MAD | 0.342 | 0.658 | 0.241 | 0.012 | 0.088 | 0.109 | 0.550 |
| 1257 | 18— | Tall | Madagascar | MAD | 0.283 | 0.717 | 0.022 | 0.051 | 0.232 | 0.082 | 0.613 |
| 1258 | 18— | Tall | Madagascar | MAD | 0.124 | 0.876 | 0.034 | 0.110 | 0.188 | 0.033 | 0.635 |
| 1259 | 18— | Tall | Madagascar | MAD | 0.796 | 0.204 | 0.065 | 0.008 | 0.018 | 0.746 | 0.164 |
| 1260 | 18— | Tall | Madagascar | MAD | 0.503 | 0.497 | 0.179 | 0.007 | 0.112 | 0.274 | 0.429 |
| 1261 | 18— | Tall | Madagascar | MAD | 0.285 | 0.715 | 0.033 | 0.007 | 0.233 | 0.164 | 0.562 |
| 1262 | 18— | Tall | Madagascar | MAD | 0.509 | 0.491 | 0.029 | 0.130 | 0.260 | 0.147 | 0.433 |
| 1263 | 18— | Tall | Madagascar | MAD | 0.639 | 0.361 | 0.044 | 0.022 | 0.591 | 0.032 | 0.312 |
| 1264 | 18— | Dwarf | Madagascar | MAD | 0.807 | 0.193 | 0.007 | 0.005 | 0.847 | 0.017 | 0.124 |
| 1265 | 18— | Tall | Madagascar | MAD | 0.025 | 0.975 | 0.008 | 0.013 | 0.215 | 0.015 | 0.749 |
| 1266 | 18— | Tall | Madagascar | MAD | 0.168 | 0.832 | 0.045 | 0.020 | 0.214 | 0.091 | 0.630 |
| 1267 | 18— | Tall | Madagascar | MAD | 0.788 | 0.212 | 0.046 | 0.347 | 0.301 | 0.039 | 0.267 |
| 1268 | 19— | Tall | Seychelles | SEY | 0.008 | 0.992 | 0.006 | 0.010 | 0.012 | 0.007 | 0.965 |
| 1269 | 19— | Tall | Seychelles | SEY | 0.014 | 0.986 | 0.006 | 0.008 | 0.010 | 0.048 | 0.928 |
| 1270 | 19— | Tall | Seychelles | SEY | 0.006 | 0.994 | 0.007 | 0.006 | 0.011 | 0.008 | 0.967 |
| 1271 | 19— | Tall | Seychelles | SEY | 0.006 | 0.994 | 0.005 | 0.004 | 0.006 | 0.005 | 0.980 |
| 1272 | 19— | Tall | Seychelles | SEY | 0.008 | 0.992 | 0.007 | 0.007 | 0.010 | 0.009 | 0.968 |
| 1273 | 19— | Tall | Seychelles | SEY | 0.010 | 0.990 | 0.013 | 0.010 | 0.011 | 0.013 | 0.954 |
| 1274 | 19— | Tall | Seychelles | SEY | 0.005 | 0.995 | 0.004 | 0.004 | 0.005 | 0.004 | 0.983 |
| 1275 | 19— | Tall | Seychelles | SEY | 0.008 | 0.992 | 0.005 | 0.005 | 0.006 | 0.028 | 0.957 |
| 1276 | 19— | Tall | Seychelles | SEY | 0.009 | 0.991 | 0.007 | 0.007 | 0.011 | 0.008 | 0.968 |
| 1277 | 19— | Tall | Seychelles | SEY | 0.008 | 0.992 | 0.007 | 0.008 | 0.008 | 0.007 | 0.970 |
| 1278 | 19— | Tall | Seychelles | SEY | 0.005 | 0.995 | 0.004 | 0.004 | 0.004 | 0.004 | 0.985 |
| 1279 | 19— | Tall | Seychelles | SEY | 0.012 | 0.988 | 0.009 | 0.018 | 0.014 | 0.010 | 0.949 |
| 1280 | 19— | Tall | Seychelles | SEY | 0.064 | 0.936 | 0.032 | 0.005 | 0.022 | 0.025 | 0.916 |
| 1281 | 19— | Tall | Seychelles | SEY | 0.013 | 0.987 | 0.015 | 0.014 | 0.010 | 0.012 | 0.949 |
| 1282 | 19— | Tall | Seychelles | SEY | 0.009 | 0.991 | 0.007 | 0.007 | 0.006 | 0.007 | 0.972 |
| 1283 | 19— | Tall | Seychelles | SEY | 0.006 | 0.994 | 0.005 | 0.006 | 0.005 | 0.005 | 0.978 |
| 1284 | 19— | Tall | Seychelles | SEY | 0.009 | 0.991 | 0.009 | 0.006 | 0.006 | 0.008 | 0.972 |
| 1285 | 19— | Tall | Seychelles | SEY | 0.006 | 0.994 | 0.005 | 0.006 | 0.005 | 0.005 | 0.979 |
| 1286 | 19— | Tall | Seychelles | SEY | 0.004 | 0.996 | 0.004 | 0.003 | 0.003 | 0.004 | 0.986 |
| 1287 | 19— | Tall | Seychelles | SEY | 0.006 | 0.994 | 0.005 | 0.007 | 0.005 | 0.005 | 0.977 |
| 1288 | 19— | Tall | Seychelles | SEY | 0.014 | 0.986 | 0.012 | 0.011 | 0.011 | 0.012 | 0.955 |
| 1289 | 19— | Tall | Seychelles | SEY | 0.009 | 0.991 | 0.007 | 0.015 | 0.010 | 0.009 | 0.958 |
| 1290 | 19— | Tall | Seychelles | SEY | 0.005 | 0.995 | 0.004 | 0.003 | 0.004 | 0.004 | 0.984 |
| 1291 | 19— | Tall | Seychelles | SEY | 0.005 | 0.995 | 0.005 | 0.004 | 0.004 | 0.005 | 0.983 |
| 1292 | 19— | Tall | Seychelles | SEY | 0.005 | 0.995 | 0.004 | 0.003 | 0.004 | 0.004 | 0.984 |
| 1293 | 19— | Tall | Seychelles | SEY | 0.005 | 0.995 | 0.004 | 0.004 | 0.004 | 0.005 | 0.983 |
| 1294 | 19— | Tall | Seychelles | SEY | 0.006 | 0.994 | 0.006 | 0.005 | 0.005 | 0.005 | 0.979 |
| 1295 | 19— | Tall | Seychelles | SEY | 0.006 | 0.994 | 0.005 | 0.006 | 0.004 | 0.005 | 0.979 |
| 1296 | 19— | Tall | Seychelles | SEY | 0.005 | 0.995 | 0.004 | 0.003 | 0.004 | 0.005 | 0.983 |
| 1297 | 19— | Tall | Seychelles | SEY | 0.008 | 0.992 | 0.006 | 0.009 | 0.008 | 0.007 | 0.970 |
| 1298 | 19— | Tall | Seychelles | SEY | 0.006 | 0.994 | 0.005 | 0.005 | 0.005 | 0.005 | 0.981 |
| 1299 | 19— | Tall | Seychelles | SEY | 0.020 | 0.980 | 0.028 | 0.008 | 0.038 | 0.035 | 0.891 |
| 1300 | 19— | Tall | Seychelles | SEY | 0.013 | 0.987 | 0.011 | 0.011 | 0.011 | 0.011 | 0.956 |
| 1301 | 19— | Tall | Seychelles | SEY | 0.296 | 0.704 | 0.027 | 0.010 | 0.452 | 0.029 | 0.481 |
| 1302 | 19— | Tall | Seychelles | SEY | 0.009 | 0.991 | 0.009 | 0.010 | 0.007 | 0.008 | 0.966 |
| 1303 | 19— | Tall | Seychelles | SEY | 0.044 | 0.956 | 0.038 | 0.004 | 0.006 | 0.025 | 0.928 |
| 1304 | 19— | Tall | Seychelles | SEY | 0.084 | 0.916 | 0.051 | 0.014 | 0.007 | 0.038 | 0.891 |
| 1305 | 19— | Tall | Seychelles | SEY | 0.009 | 0.991 | 0.007 | 0.007 | 0.007 | 0.008 | 0.971 |
| 1306 | 19— | Tall | Seychelles | SEY | 0.004 | 0.996 | 0.004 | 0.003 | 0.004 | 0.004 | 0.985 |
| 1307 | 19— | Tall | Seychelles | SEY | 0.004 | 0.996 | 0.004 | 0.003 | 0.003 | 0.004 | 0.986 |
| 1308 | 19— | Tall | Seychelles | SEY | 0.005 | 0.995 | 0.004 | 0.004 | 0.004 | 0.004 | 0.985 |
| 1309 | 19— | Tall | Seychelles | SEY | 0.009 | 0.991 | 0.005 | 0.005 | 0.005 | 0.026 | 0.959 |
| 1310 | 19— | Tall | Seychelles | SEY | 0.005 | 0.995 | 0.004 | 0.004 | 0.005 | 0.004 | 0.983 |
| 1311 | 19— | Tall | Seychelles | SEY | 0.005 | 0.995 | 0.004 | 0.004 | 0.004 | 0.004 | 0.985 |
| 1312 | 19— | Tall | Seychelles | SEY | 0.007 | 0.993 | 0.007 | 0.005 | 0.005 | 0.007 | 0.975 |
| 1313 | 19— | Tall | Seychelles | SEY | 0.005 | 0.995 | 0.004 | 0.004 | 0.004 | 0.005 | 0.982 |
| 1314 | 19— | Tall | Seychelles | SEY | 0.007 | 0.993 | 0.008 | 0.008 | 0.007 | 0.007 | 0.970 |
| 1315 | 19— | Tall | Seychelles | SEY | 0.005 | 0.995 | 0.004 | 0.004 | 0.004 | 0.004 | 0.984 |
| 1316 | 19— | Tall | Seychelles | SEY | 0.102 | 0.898 | 0.014 | 0.012 | 0.391 | 0.011 | 0.573 |
| 1317 | 19— | Tall | Seychelles | SEY | 0.014 | 0.986 | 0.011 | 0.008 | 0.031 | 0.011 | 0.939 |
| 1318 | 19— | Tall | Seychelles | SEY | 0.013 | 0.987 | 0.011 | 0.011 | 0.011 | 0.011 | 0.956 |
| 1319 | 19— | Tall | Seychelles | SEY | 0.008 | 0.992 | 0.007 | 0.007 | 0.007 | 0.007 | 0.972 |
| 1320 | 19— | Tall | Seychelles | SEY | 0.008 | 0.992 | 0.007 | 0.007 | 0.006 | 0.007 | 0.972 |
| 1321 | 19— | Tall | Seychelles | SEY | 0.021 | 0.979 | 0.018 | 0.016 | 0.016 | 0.016 | 0.933 |
| 1322 | 19— | Tall | Seychelles | SEY | 0.010 | 0.990 | 0.008 | 0.008 | 0.008 | 0.008 | 0.967 |
