# Supplementary material for: Independent Origins of Cultivated Coconut (Cocos nucifera L.) in the Old World Tropics
Source: PLoS One. 2011 Jun 22;6(6):e21143. doi: 10.1371/journal.pone.0021143 (PMC3120816; doi:10.1371/journal.pone.0021143)
Supplement: Table S2 — Allele frequencies for each locus for Pacific and western Indian Ocean populations. (DOC) [file pone.0021143.s004.doc]

**Supporting information**

**Table S2. Analysis of Molecular Variance (AMOVA) for all coconut accessions (1322 individuals).** ‘Populations’ refer to the 19 GCP/CIRAD groups and additional samples identified in Table 1.

| **Source** | **df** | **SS** | **MS.** | **Est. Var** | **%** |
| --- | --- | --- | --- | --- | --- |
| Among regions | 1 | 2648.54 | 2648.5 | 4.225 | 33 |
| Among populations | 17 | 1999.72 | 117.63 | 1.845 | 15 |
| Within populations | 1303 | 8567.66 | 6.57 | 6.575 | 52 |
|  |  |  |  |  |  |
| **Total** | **1321** | **13215.93** | **0.872** | **12.64** | **100** |
