# Supplementary material for: Independent Origins of Cultivated Coconut (Cocos nucifera L.) in the Old World Tropics
Source: PLoS One. 2011 Jun 22;6(6):e21143. doi: 10.1371/journal.pone.0021143 (PMC3120816; doi:10.1371/journal.pone.0021143)
Supplement: Table S3 — Analysis of Molecular Variance (AMOVA) for all coconut accessions (1322 individuals). (DOC) [file pone.0021143.s005.doc]

**Supporting information**

**Table S3. Allele frequencies for each locus for Pacific and western Indian Ocean populations.** Shannon’s mutual information is calculated for A3 vs. B1. Alleles selected as diagnostic for Pacific (A3) or Indian Ocean (B1) subpopulations are highlighted in yellow.

| **Locus** | **Allele/n** | **A3** | **B1** | **B2** | **COM** | **MAD** | **SEY** | **Mutual information** |
| --- | --- | --- | --- | --- | --- | --- | --- | --- |
| **F2** | **N** | 180 | 150 | 144 | 13 | 44 | 50 |  |
|  | **189** | 0.000 | 0.000 | 0.000 | 0.038 | 0.000 | 0.010 | 0.000 |
|  | **193** | 0.025 | 0.670 | 0.674 | 0.654 | 0.625 | 0.950 | 0.390 |
|  | **195** | 0.139 | 0.000 | 0.000 | 0.000 | 0.000 | 0.010 | 0.073 |
|  | **197** | 0.006 | 0.007 | 0.003 | 0.000 | 0.000 | 0.000 | 0.000 |
|  | **199** | 0.161 | 0.000 | 0.000 | 0.000 | 0.000 | 0.000 | 0.086 |
|  | **201** | 0.031 | 0.000 | 0.000 | 0.000 | 0.000 | 0.000 | 0.016 |
|  | **203** | 0.236 | 0.190 | 0.115 | 0.077 | 0.136 | 0.000 | 0.002 |
|  | **205** | 0.236 | 0.130 | 0.198 | 0.192 | 0.193 | 0.030 | 0.014 |
|  | **207** | 0.019 | 0.003 | 0.003 | 0.038 | 0.023 | 0.000 | 0.005 |
|  | **209** | 0.017 | 0.000 | 0.000 | 0.000 | 0.000 | 0.000 | 0.009 |
|  | **211** | 0.131 | 0.000 | 0.007 | 0.000 | 0.000 | 0.000 | 0.069 |
|  | **237** | 0.000 | 0.000 | 0.000 | 0.000 | 0.023 | 0.000 | 0.000 |
| **C12** | **N** | 175 | 145 | 145 | 12 | 43 | 48 |  |
|  | **153** | 0.000 | 0.000 | 0.000 | 0.000 | 0.000 | 0.010 | 0.000 |
|  | **161** | 0.003 | 0.000 | 0.000 | 0.000 | 0.000 | 0.000 | 0.002 |
|  | **163** | 0.283 | 0.021 | 0.066 | 0.208 | 0.070 | 0.000 | 0.112 |
|  | **167** | 0.006 | 0.834 | 0.614 | 0.375 | 0.465 | 0.771 | 0.631 |
|  | **169** | 0.003 | 0.000 | 0.000 | 0.000 | 0.000 | 0.000 | 0.002 |
|  | **171** | 0.051 | 0.003 | 0.000 | 0.000 | 0.012 | 0.000 | 0.019 |
|  | **173** | 0.431 | 0.083 | 0.131 | 0.250 | 0.186 | 0.052 | 0.123 |
|  | **175** | 0.034 | 0.034 | 0.014 | 0.000 | 0.000 | 0.000 | 0.000 |
|  | **177** | 0.014 | 0.000 | 0.003 | 0.000 | 0.000 | 0.000 | 0.007 |
|  | **181** | 0.000 | 0.000 | 0.000 | 0.083 | 0.012 | 0.021 | 0.000 |
|  | **183** | 0.174 | 0.024 | 0.172 | 0.083 | 0.233 | 0.115 | 0.051 |
|  | **193** | 0.000 | 0.000 | 0.000 | 0.000 | 0.023 | 0.031 | 0.000 |
| **E10** | **N** | 180 | 150 | 145 | 12 | 43 | 51 |  |
|  | **224** | 0.003 | 0.000 | 0.000 | 0.000 | 0.000 | 0.000 | 0.002 |
|  | **226** | 0.064 | 0.000 | 0.007 | 0.000 | 0.000 | 0.000 | 0.033 |
|  | **232** | 0.036 | 0.083 | 0.097 | 0.125 | 0.035 | 0.020 | 0.007 |
|  | **234** | 0.000 | 0.000 | 0.003 | 0.042 | 0.023 | 0.020 | 0.000 |
|  | **236** | 0.039 | 0.010 | 0.069 | 0.042 | 0.140 | 0.010 | 0.007 |
|  | **238** | 0.753 | 0.083 | 0.290 | 0.333 | 0.267 | 0.029 | 0.371 |
|  | **240** | 0.017 | 0.000 | 0.003 | 0.083 | 0.012 | 0.000 | 0.009 |
|  | **242** | 0.003 | 0.000 | 0.000 | 0.000 | 0.000 | 0.000 | 0.002 |
|  | **244** | 0.081 | 0.767 | 0.514 | 0.375 | 0.512 | 0.922 | 0.389 |
|  | **246** | 0.006 | 0.057 | 0.017 | 0.000 | 0.012 | 0.000 | 0.018 |
| **A9** | **N** | 180 | 150 | 145 | 9 | 37 | 36 |  |
|  | **89** | 0.486 | 0.037 | 0.117 | 0.333 | 0.189 | 0.000 | 0.215 |
|  | **91** | 0.003 | 0.000 | 0.007 | 0.000 | 0.000 | 0.000 | 0.002 |
|  | **93** | 0.028 | 0.000 | 0.003 | 0.000 | 0.000 | 0.000 | 0.014 |
|  | **95** | 0.022 | 0.000 | 0.000 | 0.000 | 0.000 | 0.083 | 0.011 |
|  | **97** | 0.100 | 0.613 | 0.559 | 0.500 | 0.541 | 0.792 | 0.224 |
|  | **99** | 0.003 | 0.247 | 0.066 | 0.111 | 0.122 | 0.125 | 0.126 |
|  | **101** | 0.000 | 0.003 | 0.041 | 0.000 | 0.000 | 0.000 | 0.002 |
|  | **103** | 0.347 | 0.100 | 0.197 | 0.056 | 0.149 | 0.000 | 0.066 |
|  | **105** | 0.008 | 0.000 | 0.010 | 0.000 | 0.000 | 0.000 | 0.004 |
|  | **107** | 0.003 | 0.000 | 0.000 | 0.000 | 0.000 | 0.000 | 0.002 |
| **C7** | **N** | 179 | 147 | 145 | 8 | 34 | 34 |  |
|  | **157** | 0.662 | 0.027 | 0.155 | 0.563 | 0.279 | 0.000 | 0.378 |
|  | **159** | 0.017 | 0.354 | 0.086 | 0.000 | 0.118 | 0.132 | 0.161 |
|  | **161** | 0.142 | 0.014 | 0.090 | 0.125 | 0.103 | 0.015 | 0.047 |
|  | **163** | 0.126 | 0.020 | 0.000 | 0.000 | 0.000 | 0.029 | 0.033 |
|  | **165** | 0.034 | 0.197 | 0.531 | 0.188 | 0.324 | 0.691 | 0.051 |
|  | **167** | 0.008 | 0.381 | 0.138 | 0.125 | 0.176 | 0.132 | 0.198 |
|  | **169** | 0.000 | 0.003 | 0.000 | 0.000 | 0.000 | 0.000 | 0.002 |
|  | **175** | 0.000 | 0.003 | 0.000 | 0.000 | 0.000 | 0.000 | 0.002 |
|  | **181** | 0.011 | 0.000 | 0.000 | 0.000 | 0.000 | 0.000 | 0.006 |
| **B6** | **N** | 177 | 150 | 146 | 10 | 38 | 42 |  |
|  | **196** | 0.003 | 0.383 | 0.127 | 0.000 | 0.013 | 0.000 | 0.213 |
|  | **198** | 0.000 | 0.027 | 0.140 | 0.000 | 0.039 | 0.107 | 0.014 |
|  | **200** | 0.023 | 0.003 | 0.003 | 0.000 | 0.013 | 0.000 | 0.006 |
|  | **202** | 0.483 | 0.047 | 0.140 | 0.500 | 0.316 | 0.024 | 0.198 |
|  | **204** | 0.034 | 0.243 | 0.106 | 0.000 | 0.092 | 0.202 | 0.073 |
|  | **206** | 0.008 | 0.240 | 0.144 | 0.050 | 0.105 | 0.548 | 0.110 |
|  | **208** | 0.401 | 0.057 | 0.322 | 0.300 | 0.237 | 0.119 | 0.133 |
|  | **210** | 0.037 | 0.000 | 0.007 | 0.150 | 0.184 | 0.000 | 0.019 |
|  | **226** | 0.011 | 0.000 | 0.010 | 0.000 | 0.000 | 0.000 | 0.006 |
| **E12** | **N** | 177 | 150 | 146 | 11 | 33 | 45 |  |
|  | **162** | 0.045 | 0.000 | 0.007 | 0.000 | 0.000 | 0.000 | 0.023 |
|  | **164** | 0.653 | 0.150 | 0.353 | 0.409 | 0.409 | 0.067 | 0.201 |
|  | **166** | 0.280 | 0.000 | 0.099 | 0.045 | 0.167 | 0.000 | 0.157 |
|  | **168** | 0.000 | 0.000 | 0.000 | 0.000 | 0.015 | 0.000 | 0.000 |
|  | **174** | 0.023 | 0.850 | 0.541 | 0.545 | 0.394 | 0.856 | 0.604 |
|  | **178** | 0.000 | 0.000 | 0.000 | 0.000 | 0.015 | 0.000 | 0.000 |
|  | **180** | 0.000 | 0.000 | 0.000 | 0.000 | 0.000 | 0.078 | 0.000 |
| **A3** | **N** | 180 | 150 | 147 | 10 | 33 | 38 |  |
|  | **218** | 0.000 | 0.000 | 0.000 | 0.000 | 0.000 | 0.132 | 0.000 |
|  | **224** | 0.000 | 0.000 | 0.000 | 0.000 | 0.000 | 0.039 | 0.000 |
|  | **226** | 0.000 | 0.000 | 0.000 | 0.000 | 0.000 | 0.039 | 0.000 |
|  | **228** | 0.072 | 0.970 | 0.680 | 0.350 | 0.424 | 0.750 | 0.715 |
|  | **230** | 0.067 | 0.000 | 0.000 | 0.000 | 0.000 | 0.013 | 0.034 |
|  | **232** | 0.047 | 0.000 | 0.000 | 0.000 | 0.000 | 0.000 | 0.024 |
|  | **234** | 0.422 | 0.017 | 0.221 | 0.400 | 0.121 | 0.000 | 0.206 |
|  | **238** | 0.017 | 0.000 | 0.000 | 0.000 | 0.000 | 0.000 | 0.009 |
|  | **240** | 0.372 | 0.013 | 0.099 | 0.250 | 0.379 | 0.026 | 0.181 |
|  | **242** | 0.003 | 0.000 | 0.000 | 0.000 | 0.045 | 0.000 | 0.002 |
|  | **248** | 0.000 | 0.000 | 0.000 | 0.000 | 0.015 | 0.000 | 0.000 |
|  | **254** | 0.000 | 0.000 | 0.000 | 0.000 | 0.015 | 0.000 | 0.000 |
| **G11** | **N** | 179 | 147 | 134 | 11 | 37 | 38 |  |
|  | **178** | 0.000 | 0.000 | 0.000 | 0.000 | 0.027 | 0.000 | 0.000 |
|  | **186** | 0.003 | 0.000 | 0.000 | 0.000 | 0.000 | 0.026 | 0.002 |
|  | **188** | 0.360 | 0.041 | 0.138 | 0.273 | 0.257 | 0.184 | 0.128 |
|  | **190** | 0.031 | 0.003 | 0.000 | 0.000 | 0.000 | 0.000 | 0.010 |
|  | **194** | 0.581 | 0.211 | 0.332 | 0.409 | 0.365 | 0.039 | 0.106 |
|  | **195** | 0.011 | 0.000 | 0.000 | 0.000 | 0.000 | 0.000 | 0.006 |
|  | **196** | 0.000 | 0.000 | 0.011 | 0.000 | 0.000 | 0.000 | 0.000 |
|  | **198** | 0.000 | 0.000 | 0.007 | 0.000 | 0.000 | 0.066 | 0.000 |
|  | **200** | 0.003 | 0.017 | 0.093 | 0.000 | 0.000 | 0.066 | 0.004 |
|  | **202** | 0.000 | 0.000 | 0.000 | 0.000 | 0.014 | 0.000 | 0.000 |
|  | **204** | 0.003 | 0.293 | 0.142 | 0.182 | 0.243 | 0.237 | 0.154 |
|  | **205** | 0.000 | 0.003 | 0.000 | 0.000 | 0.000 | 0.000 | 0.002 |
|  | **206** | 0.000 | 0.003 | 0.037 | 0.000 | 0.000 | 0.039 | 0.002 |
|  | **208** | 0.008 | 0.405 | 0.190 | 0.136 | 0.068 | 0.079 | 0.214 |
|  | **210** | 0.000 | 0.020 | 0.049 | 0.000 | 0.000 | 0.263 | 0.010 |
|  | **212** | 0.000 | 0.003 | 0.000 | 0.000 | 0.027 | 0.000 | 0.002 |
| **H7** | **N** | 179 | 149 | 145 | 13 | 37 | 47 |  |
|  | **127** | 0.011 | 0.000 | 0.000 | 0.000 | 0.000 | 0.000 | 0.006 |
|  | **131** | 0.000 | 0.000 | 0.003 | 0.000 | 0.000 | 0.000 | 0.000 |
|  | **133** | 0.028 | 0.235 | 0.390 | 0.154 | 0.176 | 0.638 | 0.076 |
|  | **135** | 0.137 | 0.523 | 0.197 | 0.038 | 0.122 | 0.330 | 0.128 |
|  | **137** | 0.050 | 0.000 | 0.007 | 0.000 | 0.014 | 0.011 | 0.025 |
|  | **139** | 0.528 | 0.094 | 0.262 | 0.423 | 0.432 | 0.021 | 0.171 |
|  | **141** | 0.218 | 0.148 | 0.141 | 0.385 | 0.257 | 0.000 | 0.006 |
|  | **143** | 0.028 | 0.000 | 0.000 | 0.000 | 0.000 | 0.000 | 0.014 |
