# Supplementary material for: Independent Origins of Cultivated Coconut (Cocos nucifera L.) in the Old World Tropics
Source: PLoS One. 2011 Jun 22;6(6):e21143. doi: 10.1371/journal.pone.0021143 (PMC3120816; doi:10.1371/journal.pone.0021143)
Supplement: Table S4 — Language roots associated with the coconut in proto-South-Dravidian and proto-Telugu. (DOC) [file pone.0021143.s006.doc]

**Supporting information**

**Table S4. Language roots associated with the coconut in proto-South-Dravidian and proto-Telugu.** Roots in bold are common to both families with identical or very similar meanings. Source: Tower of Babel Project <http://starling.rinet.ru/main.html>

| **Root** | **Meaning** |
| --- | --- |
| **Proto-South Dravidian** | |
| ***teŋ-** | **Coconut** |
| ***kobbar-ai** | **dried coconut kernel, copra** |
| *ǵiraṭ-ai (?) | coconut shell, begging bowl |
| *toṭ-ai | basket made of coconut leaves |
| *pānd- | fibres of coconut branch (petiole?) |
| *vaẓ(u)k-ai | pulp of a tender coconut |
| *vIḍal-ai | coconut |
| **Proto-Telugu** | |
| ***teŋ-** | **Coconut** |
| ***kobbar-** | **kernel of the coconut; pertaining to the coconut** |
| *agapa | ladle, spoon, scoop made of coconut shell |
| *kuriḍ- | dried whole kernel of coconut |
| *tur_- | to scrape with a toothed instrument as the kernel of a coconut |
| *pāḷ-a | husk of a coconut |
