# Supplementary material for: Independent Origins of Cultivated Coconut (Cocos nucifera L.) in the Old World Tropics
Source: PLoS One. 2011 Jun 22;6(6):e21143. doi: 10.1371/journal.pone.0021143 (PMC3120816; doi:10.1371/journal.pone.0021143)
Supplement: Text S1 — Early evidence of coconut use in the southern Indian subcontinent and neighboring islands. (DOC) [file pone.0021143.s007.doc]

**Supporting information**

**Text S1. Early evidence of coconut use in the southern Indian subcontinent and neighboring islands**

Coconut is known in Tamil as *tenkay*, with many cognates (*ten, tenka tennai*) in other South-Dravidian languages. *Ten-kay* is a proto-South-Dravidian root, which can be translated literally as “southern fruit.” It is reasonable to propose that coconut cultivation began after the southern branch of the Dravidian family split from the northern and central branches, around the mid-second millennium BCE . It was not later than (and possibly long before) the second century BCE, when legends involving coconut were written . Coconut is said to have been introduced to Malabar by insular people, presumably from Sri Lanka. There were political relationships between Sri Lanka and the Maldives, suggesting a potential role for the more southerly islands as well. In Persian and Arabic travel accounts, these islands and the Laccadives were collectively referred to as the Dibadjat . Besides *tenkay,* additional proto-South-Dravidian and proto-Telugu roots include names for a coconut shell, a basket made of coconut leaf, fiber from a coconut “branch” (i.e. petiole), a tender coconut, and a dry coconut kernel (see Table S4). The latter, **kobbari*, (*kobbara* in Malayalam) was transmitted as *kopra* to modern Hindi and to English as *copra.* This diversity of coconut-related roots within the proto-languages attests to the early importance of coconut cultivation and utilization in the southern Indian subcontinent and neighboring islands.

Within the Indo-European language family, a variant of the Sanskrit word *narikela*, found in the Ramayana (first century BCE) was transmitted as *nargil* to the Arabs, who, in turn, introduced the word to East Africa (*nazi*, *mnazi* or *munazi* in Bantu languages. The absence of the widely used Austronesian word (*Nyiur* in Indonesian, *niu*, *nu* etc. in Polynesian languages, *voanio* in Malagasy) in the Indian terminology provides a further argument in favour of an independent South Asian domestication. The historic cultural importance of coconuts in South Asia is also evident in the Ayurveda, Indian mythology, and cuisine .

Archaeological evidence of sennit (twisted coconut fiber) ropes and coconut shells from Arikamedu (Pondicherry), along the Coromandel Coast, dating to first century BCE , suggests that coconut husks were being used for twine production in the south of India over two millennia ago. The impetus for rope making from coir was likely propelled by navigational advances, which required salt-water resistant cordage for rigging and for construction of sailing ships made of hewn coconut wood “sewn” together with sennit (see . The earliest known record of coconuts in nearby Sri Lanka was likely in the “Mahawansa” (historic chronicle of Sri Lanka) during the reign of Dutthagamani, 101-77 BCE ; ancient accounts from pre-Portuguese travelers during the 6^th^ Century CE further support its antiquity in Sri Lanka.

To the south, Maldivians were renowned for their boat building (*dhoni banum* [tying]) and their knowledge of the monsoons, enabling them to become seafaring navigators and early Indian Ocean exporters of coconuts, coir, cowries and ambergris. The nearby Laccadives were known as *Diva-kanbar* (Coir Islands) by Arab geographers and writers from the 10th Century CE . These islands served as ports for foreign trading vessels from China, Southeast Asia, Arabia, East Africa and Rome, providing shelter from monsoon storms and stopping points for ship repairs and ropes. By 1-200 CE, India and Sri Lanka were already established trading centers between the Mediterranean and Southwest Asia. Roman emporia like Berenike (1-600 CE), situated on the Red Sea coast in the Eastern Desert of Egypt, are known to have imported coconuts, black pepper and rice from these regions of South Asia .

Given the coconut’s key role as a source of construction materials in southern coastal India and neighboring archipelagos, coconut domestication in this region would likely have involved selection for varieties with long trunks for lumber, and abundant fruits with high husk-to-nut ratios for coir production. These traits stand in contrast to those hypothesized by Harries for domestication in the ‘Malesian’ region. As a result, indigenous South Asian coconuts are composed nearly exclusively of varieties with the Tall growth habit and elongated angular fruits with thick husks . The West Coast Tall from Kerala and the Laccadive Ordinary Tall are good examples. The fruit of another variety of the same island (the Laccadive Micro Tall) is 20 cm long and is only a little larger than the 13 cm fossil fruit found in Tertiary sediments of Amarkantak .

**References**

1. Fuller D (2007) Non-human genetics, agricultural origins and historical linguistics in South Asia. In: Petraglia MD, Allchin B, editors. The evolution and history of human populations in South Asia. Dordrecht, The Netherlands: Springer. pp. 393-443.

2. Mahdi W (1998) Linguistic data on transmission of Southeast Asian cultigens to India and Sri Lanka. In: Blench R, Spriggs M, eds. Archaeology and language II: Correlating archaeological and linguistic hypotheses. New York, NY: Routledge. pp. 390-415.

3. Ferrand G (1913) . Relations de voyages et textes géographiques arabes, persans et turks relatifs à l’extrême-Orient du 8^e^ au 18^e^ siècles (Travel accounts and geographical texts of Arabs, Persians and Turks in the Far East from the 8^th^ to 18^th^ Centuries). Paris, France: Ernest Leroux. 743 pp.

4. Menon KP, Pandalai KM (1958) The coconut palm, a monograph. Kerala, South India: Indian Central Coconut Committee. 384 p.

5. Wheeler REM, Ghosh A, Deva K (1946) Arikamedu: an Indo-Roman trading station on east coast of India. Ancient India: Bulletin of the Archaeological Survey of India 2: 17-124.

6. Maniku M (1998) Evolution of the maritime craft and boatbuilding technology in the Maldives. AIMA Bulletin 22: 9-18.

7. Geiger W (1912) The Mahavamsa; trans Geiger W, Bode MH. Oxford, UK: Oxford University Press. 367 p.

8. Yule H (1873) Cathay and the way thither, 2nd Ed. London, UK: Hakluyt Society. 1338 p.

9. Mohamed N (2005) Maldivian seafaring in the pre-Portuguese period. 10th International Conference on Sri Lanka Studies: Sri Lanka after 500 Years of Western Colonization and Future Perspectives. Kelaniya, Sri Lanka: University of Kelaniya Press.

10. Cappers R (2006) Roman foodprints at Berenike: Archaeobotanical evidence of subsistence and trade in the Eastern Desert of Egypt. Los Angeles, CA: UCLA Costen Institute of Archaeology. 229 p.

11. Harries H (1978) The evolution, dissemination and classification of *Cocos nucifera*. L. Bot Rev 44:265-319.

12. Anonymous. 1995. Coconut descriptors. Central Plantation Crop Research Institute. Kasaragod, India: Indian Council of Agricultural Research (ICAR). 197 p.

13. Tripathi RP, Mishra SN, & Sharma BD (1999) *Cocos nucifera*-like petrified fruit from the Tertiary of Amarkantak, M.P., India. Paleobotanist 48: 251-255.
